# Nickel-Catalyzed Reductive Cyanation of Aryl Halides and Epoxides with Cyanogen Bromide

**DOI:** 10.3390/molecules29246016

**Published:** 2024-12-20

**Authors:** Yu-Juan Wu, Chen Ma, Muhammad Bilal, Yu-Feng Liang

**Affiliations:** School of Chemistry and Chemical Engineering, Shandong University, Jinan 250100, China; 17865916836@163.com (Y.-J.W.); chenma@sdu.edu.cn (C.M.); muhammadbilalgcuf@gmail.com (M.B.)

**Keywords:** nickel catalysis, nitriles, cyanogen bromide, reductive coupling, cyanation

## Abstract

Nitriles are valuable compounds because they have widespread applications in organic chemistry. This report details the nickel-catalyzed reductive cyanation of aryl halides and epoxides with cyanogen bromide for the synthesis of nitriles. This robust protocol underscores the practicality of using a commercially available and cost-effective cyanation reagent. A variety of aryl halides and epoxides featuring diverse functional groups, such as -TMS, -Bpin, -OH, -NH_2_, -CN, and -CHO, were successfully converted into nitriles in moderate-to-good yields. Moreover, the syntheses at gram-scale and application in late-stage cyanation of natural products and drugs reinforces its potentiality.

## 1. Introduction

**Scheme 1:**
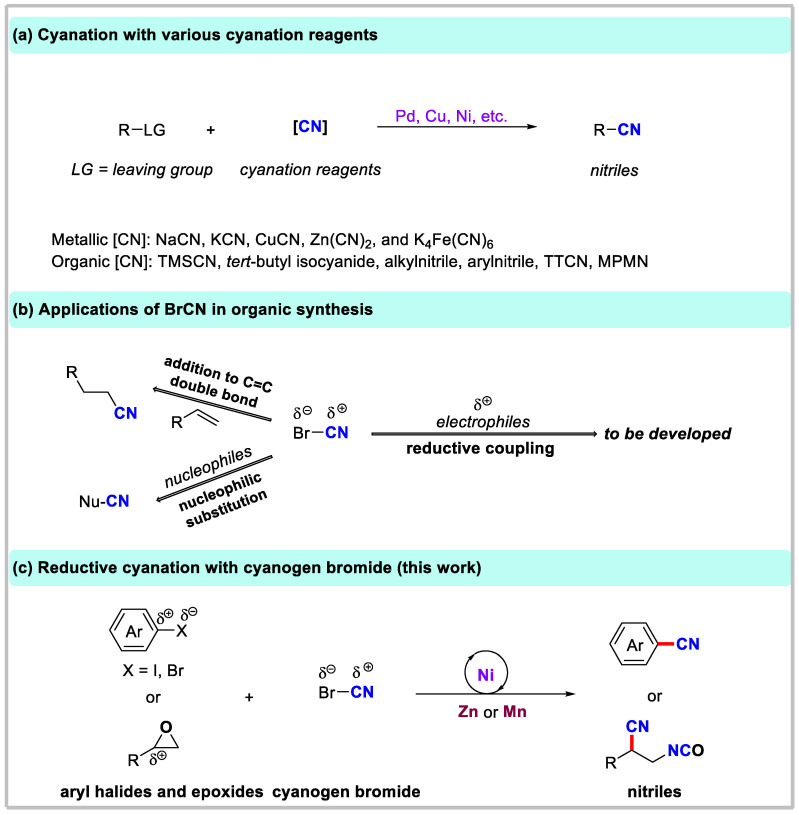
Preparation of nitriles via cyanation.

Nitriles are among the most important organic molecules, playing crucial roles in pharmaceuticals, chemical industries, and electronic materials [1,2,3,4,5,6,7]. Additionally, the CN group serves as a vital intermediate in producing amides, amines, carboxyl groups, and tetrazole derivatives [8]. Transition metal catalyzed cyanation is a widely utilized approach for the preparation of aromatic nitriles [9,10,11]. Metal cyanide precursors such as NaCN, KCN, CuCN, Zn (CN)_2_, and K_4_Fe (CN)_6_ have been explored [12,13,14,15]. However, their application is limited due to the stringent reaction conditions required [16,17] (Scheme 1a).

These limitations have prompted the search for more efficient and environmentally benign methods for cyanation. The development of new catalytic systems and the utilization of alternative cyanating reagents could significantly advance the field of nitrile synthesis. In this regard, scientists made efforts to develop environmentally friendly and readily available organic nitriles. Unlike metal cyanide precursors, TMSCN [18,19,20,21], *tert*-butyl isocyanide [22], alkylnitrile [23,24,25,26,27,28], arylnitrile [29,30,31], TTCN [32], NCTS [33,34,35], and MPMN [36] could avoid the deactivation of transition metal catalysts that can occur with a high concentration of cyanide ions (Scheme 1b). While the utilization of these organic cyanating sources offers improved methods for introducing cyano motifs, challenges persist in the form of pre-synthesized cyanating reagents, strong bases, high temperature, and operational complexities. Generally, a pseudohalogen is defined as a polyatomic compound that exhibits chemical properties analogous to those of the halogens X_2_ or interhalogens XY (such as Cl_2_, Br_2_, BrCl) [37]. The non-symmetric pseudohalogen cyanogen bromide (BrCN) was utilized in earlier years as a selective reagent for cleaving peptide bonds at methionine amino acid residues [38,39]. Over the years, electrophilic cyanogen bromide was developed as a cyanation reagent [40,41]. Although BrCN has found applications in organic synthesis, its potential in the field of reductive coupling remains to be explored (Scheme 1b).

Thus, although significant progress has been made towards nitrile synthesis, an efficient and robust catalytic system is still highly desirable. With our continuous interest in transition metal promoted cross-electrophile coupling under reductive conditions [42,43,44,45,46,47,48], herein we report a nickel-catalyzed reductive cyanation that enables the synthesis of nitriles from aryl halides and epoxides using BrCN, with excellent functional group tolerance and good atomic economy under simple and mild reaction conditions. It is worth noting that both Csp^2^−CN and Csp^3^−CN bonds can be constructed by this reaction strategy (Scheme 1c).

## 2. Results and Discussion

In our preliminary investigation, we used the coupling of ethyl 4-iodobenzoate (**1a**) with BrCN as a model reaction (Table 1). To illustrate the impact of different reaction parameters on the results, we performed a series of experiments where the conditions varied from the optimized ones (entry 1). Among all the catalysts tested for this cyanation reaction, NiCl_2_·1,10-phen emerged as the most effective option (entries 2–6). While using other nitrogen ligands (**L1**−**L2**), compound **2a** was obtained in comparatively lower yields, and phosphine or carbene ligand (**L3**−**L4**) failed to promote the transformation (entries 7−10). Solvent screening suggested that dioxane is the optimal solvent (entries 11−12). Moreover, lowering the reaction temperature or Mn powder used as the reducing agent was found to be detrimental to the reaction (entries 13−14). The control reactions conducted without Zn did not produce any desired product, indicating that Zn plays a crucial role in this transformation, and the reaction will not occur without the nickel catalyst (entry 15).

**Scheme 2:**
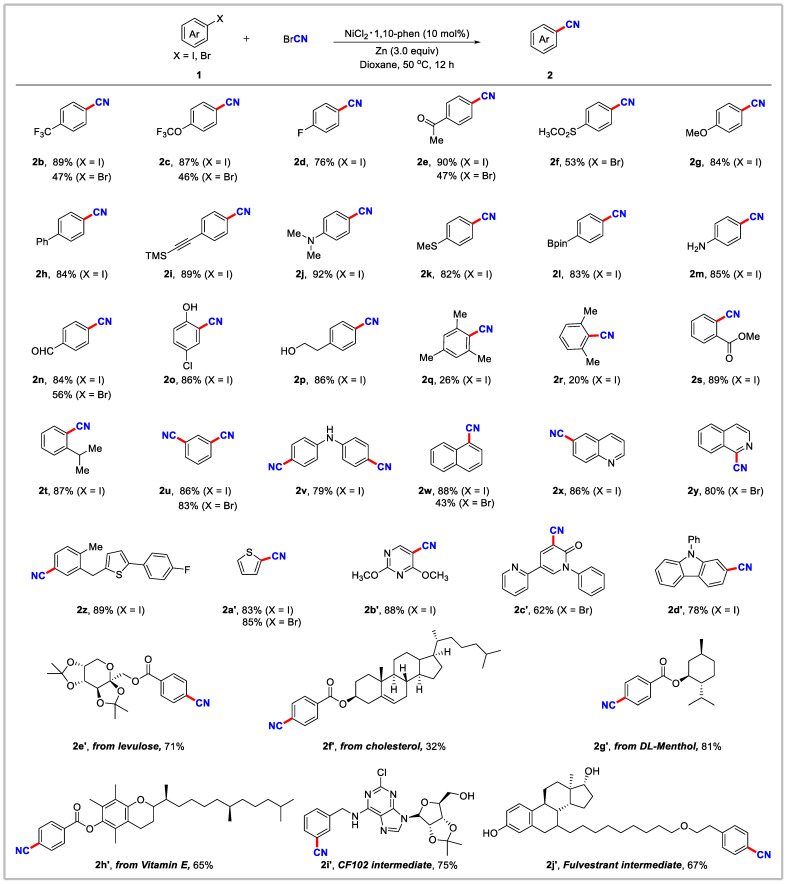
Substrate scope for aromatic nitriles. Reaction conditions: **1** (0.2 mmol), BrCN (0.4 mmol), NiCl_2_·1,10-phen (10 mol%), Zn (3.0 equiv), and dioxane (0.5 mL) at 50 °C for 12 h. Isolated yields.

Having established the optimized reaction conditions, we proceeded to assess the substrate scope of this reaction. A series of aryl iodides and aryl bromides were tested to evaluate their effects on reactivity (Scheme 2). Electron-deficient aryl iodides bearing various functional groups (e.g., trifluoromethyl and ketone) readily participated in this reaction (**2b**–**2e**). Moreover, a diverse array of electron-rich iodides with various substituents at the *para*-position of the benzene ring (e.g., methoxy, dimethylamino, and thiomethyl) were also readily accommodated (**2g**–**2k**). Notably, the valuable alkyne (**2i**), -Bpin (**2l**), and -CHO (**2n**) groups remained intact. Significantly, the reaction also afforded the functional group containing active hydrogen including -NH_2_ (**2m**) and -OH (**2o**, **2p**). Of particular note, highly hindered aryl iodides, such as 2-iodo-1,3-dimethylbenzene and 2,4,6-trimethyliodobenzene, also reacted, albeit in low yields (**2q**–**2t**). In addition to the above-mentioned monocyano products, diiodo- and dibromo-benzonitriles were competent substrates and provided the corresponding dicyano products **2u** and **2v** with high reaction efficiencies. Moreover, heteroatom-containing aryl iodides and aryl bromides, such as those with pyrimidine, carbazole, quinoline, and thiophene groups, were productive in the transformation (**2w**–**2d’**). Furthermore, some complex aryl halides derived from natural products (levulose, cholesterol, menthol, and vitamin E) and drugs (CF102 intermediate and Fulvestrant intermediate) exhibited favorable reactivity and afforded the corresponding aryl nitriles in significant yields, demonstrating good functional group tolerance and practicability of the transformation.

**Scheme 3:**
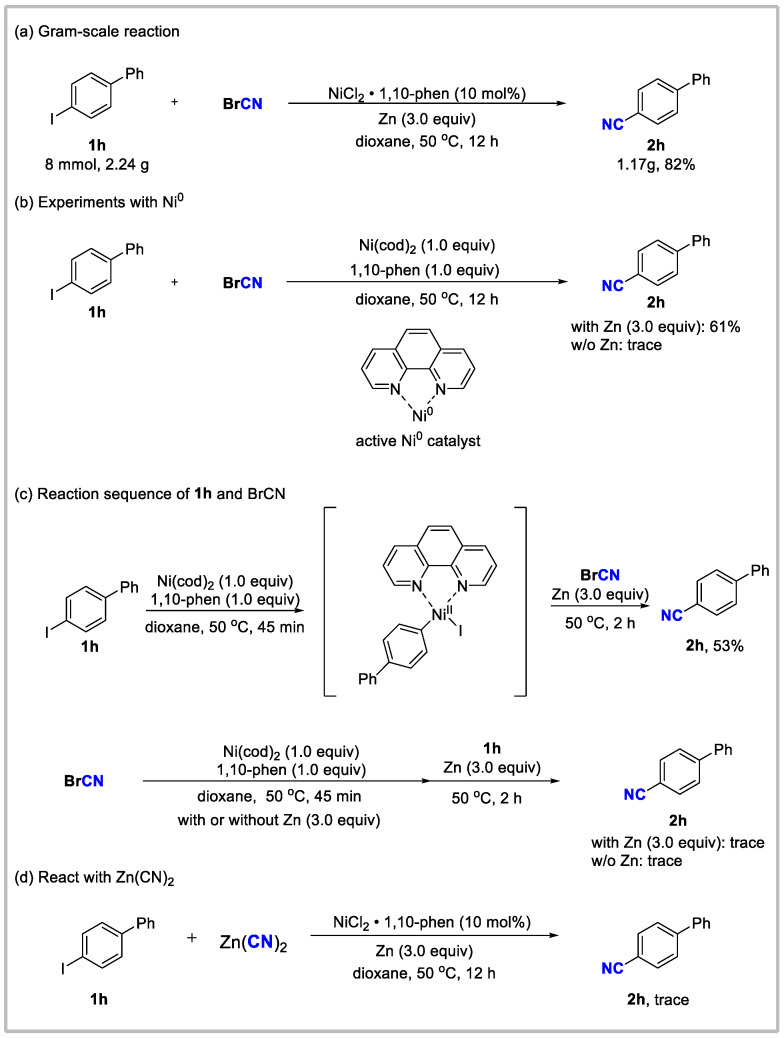
Mechanistic studies for cyanation of aryl halides.

A gram-scale reaction of **1h** with BrCN was performed under the standard conditions to result in an 82% yield, demonstrating the practical applicability of this method (Scheme 3a). To shed light on the mechanism of this reaction, we conducted the following experiments. First, we performed the reaction between 4-iodo-1,1′-biphenyl (**1h**) and BrCN using a stoichiometric amount of Ni^0^ catalyst, specifically Ni(cod)_2_, in the presence of Zn powder. This reaction yielded the cross-coupling product **2h** in 61%. However, only trace amounts of the desired product were observed in the absence of Zn (Scheme 3b). These results suggest that the following: (a) the in situ formation of the active Ni^0^ catalyst is more effective than the direct use of a Ni^0^ catalyst; and (b) the Ni^0^ to Ni^II^ and Ni^II^ to Ni^0^ cycle is not favored, indicating that the one-electron reduction in the Ni^II^ intermediate by Zn to form Ni^I^ intermediate is involved in the catalytic process. In addition, the sequential substrate addition experiments were performed, suggesting that Ni^0^ species underwent oxidative addition with aryl iodides firstly [49,50], then reacted with BrCN to create the cyanation product (Scheme 3c). The reductive coupling with Zn(CN)_2_ under the standard conditions generated the product in trace amounts, indicating that the involvement of the Zn(CN)_2_ intermediate could be excluded in this transformation [51,52,53] (Scheme 3d).

Encouraged by the above results, we then examined the reaction of epoxides with BrCN. To our delight, epoxides could be successfully converted into the isocyanate and nitrile dual functionalized products. In our preliminary investigation, we used styrene oxide (**3a**) with BrCN as a model reaction (Table 2). Of all the catalysts evaluated for the cyanation reaction, NiBr_2_ proved to be the most effective choice (entries 2–4). The efficiency of the reaction with triazole, phosphine, carbene, or phenanthroline ligand was very low (entry 5). The addition of LiI or NaI decreased the yield (entry 6). Moreover, the addition of water as an additive into the reaction would lead to significant decrease in the reaction yield (entry 7). While Et_3_N or DIPEA can promote this transformation, their efficiency was significantly lower than that of DBU (entry 8). The employment of Zn as the reducing agent led to trace amounts of the product (entry 9). Further experimentation on the solvent and temperature effect suggested that dioxane and 50 °C is the optimal choice (entries 10–11). The control experiments indicated the importance of each reaction component except the ligand (entries 12–13). Specifically, in the absence of DBU, **3a** could not be consumed (entry 14).

**Scheme 4:**
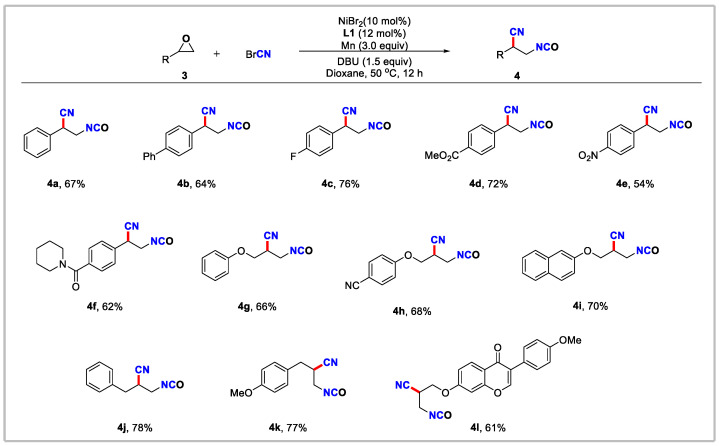
Substrate scope of epoxides. Reaction conditions: **3** (0.2 mmol), BrCN (0.6 mmol), NiBr_2_ (10 mol%), **L1** (12 mol%), Mn (3.0 equiv), DBU (1.5 equiv), and dioxane (0.5 mL) at 50 °C for 12 h. Isolated yields.

Under the standard reaction conditions using styrene oxides, both electron-neutral and electron-deficient oxides were well tolerated, affording products **4a**–**4f** in moderate yields (Scheme 4). The scope of terminal aliphatic epoxides was also examined. The reaction of electronic-rich, -neutral, and -deficient oxides provided linear products (**4g**–**4k**) in good yields. Notably, this protocol was amenable to late-stage coupling as showcased in Formononetin (**4l**). Notably, many functional groups such as ester, nitro, amide, and cyano were well tolerated in this transformation.

**Scheme 5:**
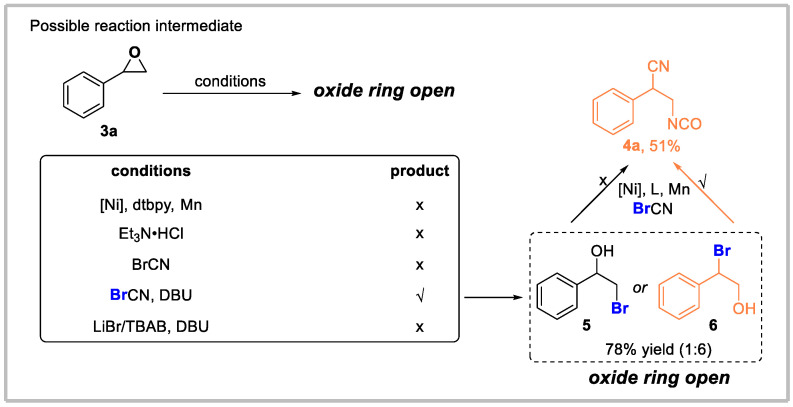
Reaction intermediate experiments.

Subsequent control experiments showed that when compound **3a** was subjected to these altered conditions, a mixture of **5** and **6** were obtained, indicating the possible involvement of a bromohydrin intermediate (Scheme 5). Moreover, when the independently synthesized bromohydrin **5** or **6** was exposed to the standard reaction conditions, only bromohydrin **6** created **4a** with a 51% yield, indicating that **6** was the key intermediate in the reaction.

**Figure 1:**
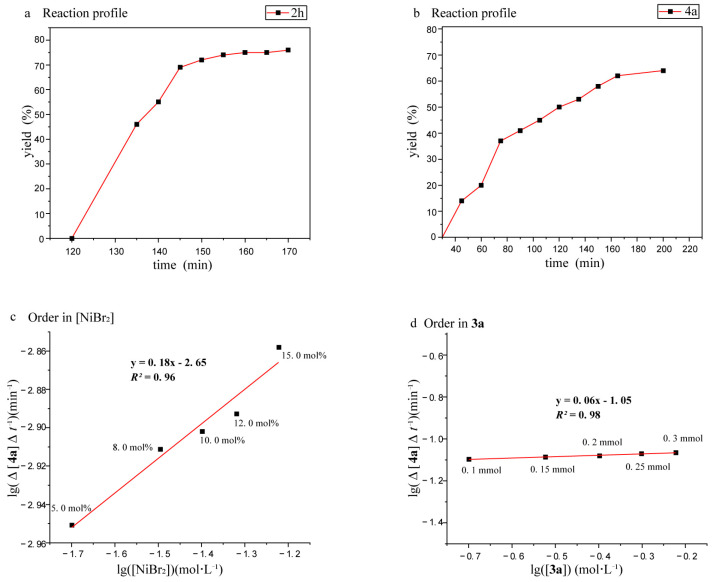
Reaction kinetics. (**a**) Reaction profile of cyanation of aryl halide **1h**; (**b**) reaction profile of cyanation of epoxide **3a**; (**c**) order in [NiBr_2_] of cyanation of epoxide **3a**; (**d**) order in [**3a**] of cyanation of epoxide.

To further clarify the reaction mechanism, we performed kinetic studies (Figure 1). The time–yield curve showed that both reactions of the aryl halides and epoxides were almost finished within three hours (Figure 1a,b). For the first two hours, no product was observed, indicating that a certain induction period is required for the generation of active species (Figure 1a). It exhibited first-order kinetics concerning the nickel catalyst (Figure 1c), and was independent of the concentrations of epoxide **3a** (Figure 1d).

**Scheme 6:**
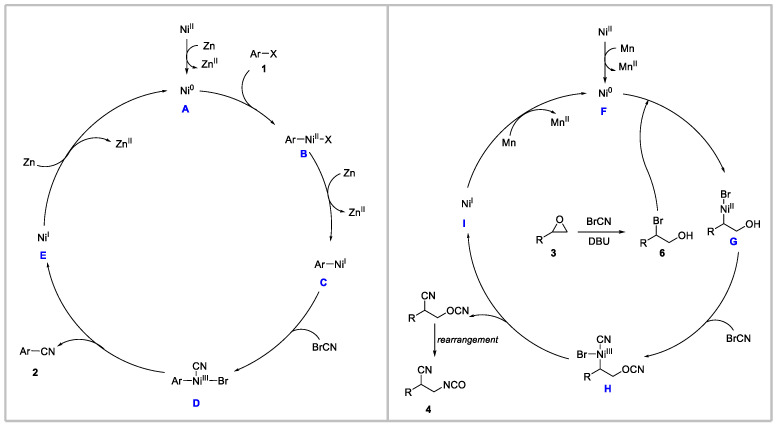
Proposed reaction mechanism.

Based on the above experiments and literature reports [54,55,56,57,58,59,60,61,62,63,64,65], a plausible reaction mechanism is proposed (Scheme 6). For the generation of aryl nitrile, initially, the active Ni^0^ species (**A**) is generated under Zn reductive conditions, followed by oxidative addition with aryl halide to form the Ni^II^ species (**B**). Subsequently, the Ni^I^ species (**C**) is regenerated by the reduction in Zn. Then, species (**C**) reacted with BrCN to afford intermediate (**D**), which would undergo reductive elimination to yield the aryl nitrile product, as well as the Ni^I^ species (**E**). Ultimately, the Ni^0^ species (**A**) is regenerated by the reduction in Zn and used in the subsequent catalytic cycle (Scheme 6, left). Concerning the reaction mechanism of the ring-opening cyanation of the epoxide, first, the active Ni^0^ species (**F**) is generated under Mn reductive conditions. Meanwhile, the ring-opening of epoxide **3** by nucleophilic DBU and BrCN forms the halohydrin intermediate **6**, which could be captured by Ni^0^ species (**F**) to form Ni^II^ species (**G**). Then, species (**G**) reacted with BrCN to afford intermediate (**H**), followed by reductive elimination to afford Ni(I) species **I** and the cross-coupled compound, which could undergo rearrangement to afford product **4** [66,67,68]. Ultimately, the Ni^0^ species (**F**) is regenerated by the reduction in Mn (Scheme 6, right). Alternatively, the process initiated from the Ni^I^ complex was also possible [69].

## 3. Materials and Methods

### 3.1. General Procedure for the Reductive Cyanation of Aryl Halides

In a 10 mL Schlenk tube, NiCl_2_·1,10-phen (7.0 mg, 0.02 mmol) and Zn power (39.2 mg, 0.6 mmol) were added sequentially. Then, aryl iodides or aryl bromides **1** (0.20 mmol) were added in dioxane (0.50 mL) under N_2_. BrCN (0.40 mmol, 2.0 M in dioxane) was added via a syringe. The resulting solution was stirred at 50 °C for 12 h. Then, the crude reaction mixture was diluted with ethyl acetate (10 mL), washed with water (5 mL), and then extracted with EtOAc (5 mL × 3). The organic layer was dried over Na_2_SO_4_, filtered, and concentrated. The residue was purified by flash chromatography to create product **2**. The general experimental details and NMR spectra of the compounds can be found in the Supplementary Materials.

### 3.2. General Procedure for the Reductive Cyanation of Epoxides

In a 10 mL Schlenk tube, NiBr_2_ (4.7 mg, 0.02 mmol), dtbpy (6.2 mg, 0.024 mmol), and Mn power (33.3 mg, 0.6 mmol) were added sequentially. Then, DBU (0.30 mmol, 45 μL) and epoxide **3** (0.20 mmol) were added in dioxane (0.50 mL) under N_2_. BrCN (0.60 mmol, 2.0 M in dioxane) was added via a syringe. The resulting solution was stirred at 50 °C for 12 h. Then, the crude reaction mixture was diluted with ethyl acetate (10 mL), washed with water (5 mL), and then extracted with EtOAc (5 mL × 3). The organic layer was dried over Na_2_SO_4_, filtered, and concentrated. The residue was purified by flash chromatography to create product **4**. The general experimental details and NMR spectra of the compounds can be found in the Supplementary Materials.

## 4. Conclusions

In summary, we have developed a nickel-catalyzed reductive cyanation of aryl halides and epoxides with cyanogen bromide. This strategy is operationally simple and furnishes practical access to nitriles with broad substrates scope under mild reaction conditions. Moreover, this efficient method could be applied to the late-stage cyanation of various complex compounds. We anticipate that this approach will serve as a useful complementary to known cyanation transformation. Further mechanistic studies and the extension of reductive cyanation to other electrophiles will be continuously explored in our laboratory.

## Figures and Tables

**Table 1:** Optimization of reaction conditions ^a^.

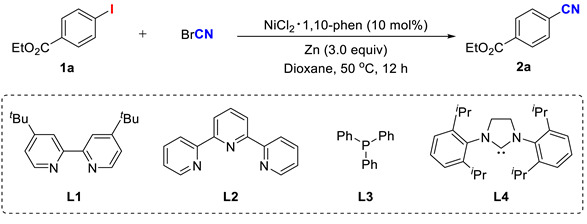		
**Entry**	**Variation in Optimized Conditions**	**Yield % ^b^**
1	none	92% (90%)
2	NiCl_2_ instead of NiCl_2_·1,10-phen	nr
3	Ni(acac)_2_ instead of NiCl_2_·1,10-phen	nr
4	NiBr_2_·DME instead of NiCl_2_·1,10-phen	nr
5	NiBr_2_·bpy instead of NiCl_2_·1,10-phen	45%
6	FeCl_3_, CoCl_2_, CrCl_2_ with 1,10-phen instead of NiCl_2_·1,10-phen	nr
7	**L1** as ligand with NiCl_2_ as catalyst	77%
8	**L2** as ligand with NiCl_2_ as catalyst	66%
9	**L3** as ligand with NiCl_2_ as catalyst	nr
10	**L4** as ligand with NiCl_2_ as catalyst	nr
11	THF instead of dioxane	88%
12	DMA instead of dioxane	84%
13	RT instead of 50 °C	trace
14	Mn instead of Zn	trace
15	w/o Zn, or w/o [Ni]	0

^a^ Reaction conditions: **1a** (0.2 mmol), BrCN (0.4 mmol), NiCl_2_·1,10-phen (10 mol%), Zn (3.0 equiv), and dioxane (0.5 mL) at 50 °C for 12 h. ^b^ GC yields (isolated yield in parentheses).

**Table 2:** Optimization of reaction conditions of epoxide **3a** with BrCN ^a^.

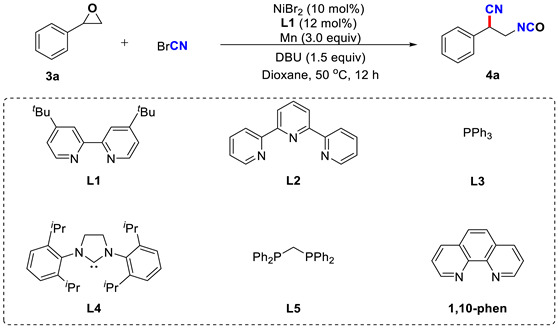		
**Entry**	**Variation in Optimized Conditions**	**Yield % ^b^**
1	none	68%
2	NiBr_2_·bpy instead of NiBr_2_	63%
3	NiBr_2_·dtbpy instead of NiBr_2_	20%
4	NiI_2_ instead of NiBr_2_	66%
5	L2, L3, L4, L5 or 1,10-phen	<20%
6	LiI or NaI as extra additive	14%, 16%
7	H_2_O (1.0 equiv) as extra additive	trace
8	Et_3_N or DIPEA instead of DBU	32%, 5%
9	Zn instead of Mn	trace
10	THF, DMA	65%, trace
11	40 °C, 60 °C	49%, 47%
12	w/o Ni, L1 or w/o Mn or w/o Ni, L1, Mn	NR
13	w/o **L1**	50%
14	w/o DBU	NR

^a^ Reaction conditions: **3** (0.2 mmol), BrCN (0.6 mmol), NiBr_2_ (10 mol%), **L1** (12 mol%), Mn (3.0 equiv), DBU (1.5 equiv), and dioxane (0.5 mL) at 50 °C for 12 h. ^b^ GC yields.

## Data Availability

The data underlying this study are available in the published article and its Supplementary Materials.

## Supplementary Materials

The following supporting information can be downloaded at: https://www.mdpi.com/article/10.3390/molecules29246016/s1. Tables S1–S3: Optimization of reaction conditions of **1a**; Tables S4–S8: Optimization of reaction conditions of **3a**, ^1^H NMR, ^13^C NMR, and ^19^F NMR spectra of all reported products. References [70,71,72,73,74,75,76,77,78,79,80,81,82,83] are cited in the Supplementary Materials.
