# Supplementary material for: Nickel-Catalyzed Reductive Cyanation of Aryl Halides and Epoxides with Cyanogen Bromide
Source: Molecules. 2024 Dec 20;29(24):6016. doi: 10.3390/molecules29246016 (PMC11678332; doi:10.3390/molecules29246016)

Supporting Information

Nickel-Catalyzed Reductive Cyanation of Aryl Halides and Epoxides with Cyanogen Bromide

Yu-Juan Wu, Chen Ma, Muhammad Bilal, and Yu-Feng Liang*

School of Chemistry and Chemical Engineering, Shandong University, Jinan 250100, China

Email: yfliang@sdu.edu.cn

Table of Contents

1. General remarks S2

2. Optimization of the reaction conditions of 1a S2

3. Optimization of the reaction conditions of 3a S4

4. Characterization data for products.. S7

5. References... S22

6. NMR spectra for products S23

1. General remarks

Analytical methods: All the reactions were monitored by thin-layer chromatography. ^{1}H NMR, ^{13}C NMR, ^{19}F NMR data were obtained on AVANCE III Bruker 500 MHz or 400 MHz nuclear resonance spectrometers unless otherwise noted. Chemical shifts (in ppm) were referenced to tetramethylsilane (TMS) (δ = 0.00 ppm) in $CDCl_3$. The data of ^{1}H NMR was reported as follows: chemical shift, multiplicity (s = singlet, d = doublet, t = triplet, q = quartet, m = multiplet), coupling constant (J values) in Hz and integration. ^{13}C NMR spectra were obtained by the same NMR spectrometers and were calibrated with $CDCl_3$ (δ = 77.00 ppm), d_6-DMSO (δ = 39.60 ppm). Flash chromatography was performed using 300-400 mesh silica gel with the indicated eluent according to standard techniques. Analysis of crude reaction mixture was done on an Agilent 7890 GC System with an Agilent 5975 Mass Selective Detector. High-resolution mass spectral (HRMS) data were recorded on Bruker APEX IV Fourier transform ion cyclotron resonance mass spectrometer using electrospray ionization (ESI) mode. Caution: Although we have encountered no dangerous in working with the cyanogen bromide, the proper precautions, such as the use of shields, fume hoods, and the avoidance of acid solvent to suppress the formation of highly toxic hydrocyanic acid, should be taken whenever possible.

Reagents: Unless otherwise noted, materials were obtained from commercial suppliers and used without further purification. Compounds **1e'-1h', 1j', 3b-3k** were prepared according to literature procedures. The starting materials are listed below:

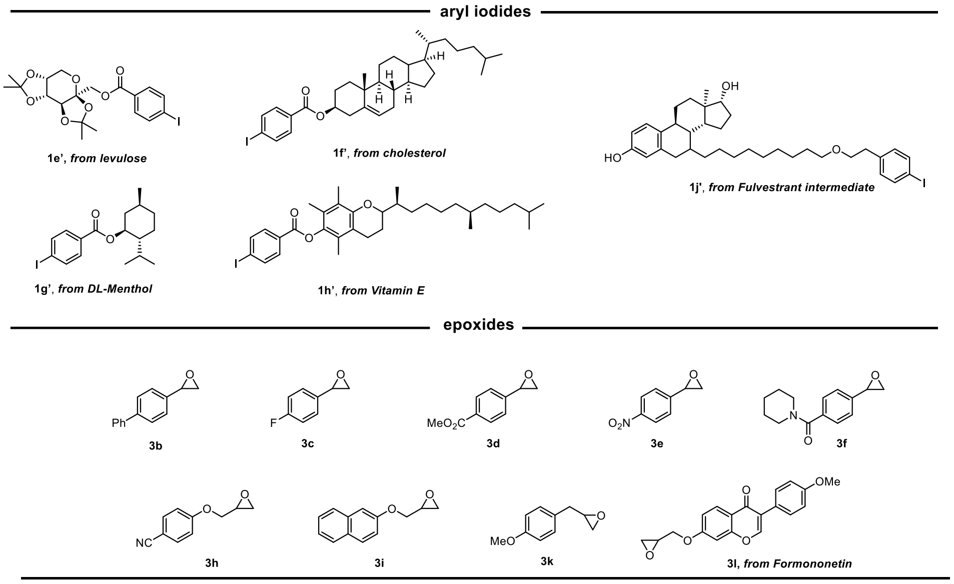

Figure S1. Starting Materials

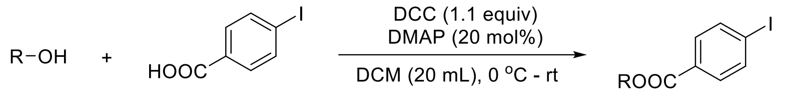

S1-S4 (10 mmol) (11 mmol) 1e'-1h'

General procedure for the preparation of 1e'-1h': to a round-bottom flask equipped with a magnetic stir bar of alcohol **S1-S4** (10 mmol), 4-dimethyl aminopyridine (DMAP, 2 mmol, 20 mol%) and *p*-iodobenzoic acid (11 mol, 1.1 equiv) in DCM (20 mL), dicyclohexylcarbodiimide (DCC, 11 mmol, 1.1 equiv) was added with an ice bath. The reaction mixture was stirred at room temperature for 24 h. Then it was filtered through a plug of silica gel and washed with ethyl acetate. The filtrate was concentrated under vacuum and the residue was purified by chromatography on silica gel (EtOAc/Hexane=10%~20%) to afford the corresponding product **1e'-1h'**.

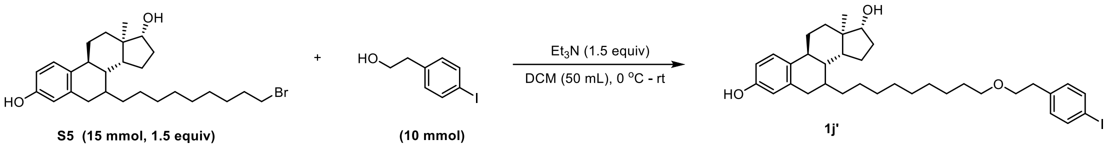

S5 (15 mmol, 1.5 equiv) (10 mmol) 1j'

Procedure for the preparation of 1j': to a round-bottom flask equipped with a magnetic stir bar was added alcohol (10 mmol, 1.0 equiv), Et_3N (15 mmol, 1.5 equiv) and DCM (50 mL). The reaction mixture was cooled to 0 °C and a solution of **S5** (15 mmol, 1.5 equiv) in DCM (5 mL) was added dropwise. The reaction mixture was then allowed to warm up to room temperature and stirred overnight. The reaction mixture was quenched with H_2O (30 mL) and extracted 3 times with DCM. The combined organic layers were dried over Na_2SO_4, filtered, and concentrated under vacuum. The crude mixture was purified by chromatography on silica gel (Hexane: EtOAc = 10:1) to afford the product **1j'**.

Procedure for the preparation of 3b:

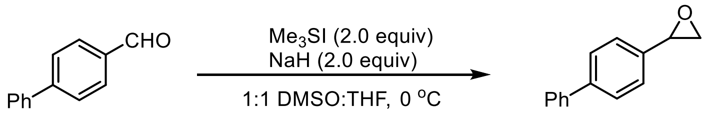

(10 mmol) 3b

To a flame-dried 200 mL round bottom flask equipped with a stir bar was charged with trimethyl sulfonium iodide (20 mmol, 2 equiv) and sodium hydride (60%, 33.3 mmol, 2 equiv). The flask was evacuated and refilled with N_2, and dry DMSO (33 mL) and THF (20 mL) were added. The reaction was stirred for 20 minutes at ambient temperature, and then cooled to 0 °C. The aldehyde (10 mmol,

1 equiv) in THF (13 mL) was added at 0 °C and the reaction was allowed to stir until complete consumption of aldehyde by TLC. The reaction was diluted with water and extracted three times with Et_2O. The combined organic layers were washed with water, then brine, dried over $MgSO_4$, filtered, and concentrated under reduced pressure. The crude mixture was purified by chromatography on silica gel (Hexane: EtOAc = 10:1) to afford the product **3b** as a white solid (523 mg, 27% yield).

General procedure for the preparation of 3c-3e, 3k:

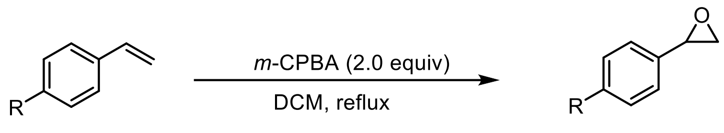

To a flame-dried 100 mL round bottom flask equipped with a stir bar was charged with styrene (10 mmol, 1 equiv) in DCM (50 mL). *m*-Chloroperbenzoic acid (20 mmol, 2 equiv) was added and the mixture was refluxed for 2 hours until the starting materiel was fully consumed by TLC. The reaction was quenched with saturated aqueous $NaHCO_3$ and extracted three times with DCM. The combined organic layers were washed with water, saturated aqueous $NaHCO_3$, then brine, dried with $MgSO_4$, filtered, and concentrated under reduced pressure. The crude mixture was purified by column chromatography (10%-20% EtOAc in hexanes)

Procedure for the preparation of 3f:

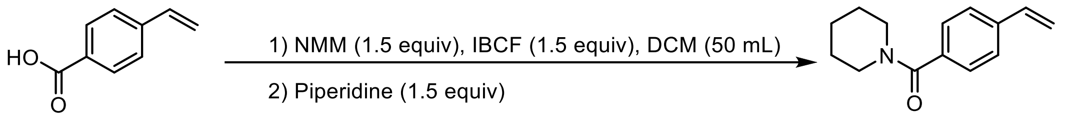

4-Vinylbenzoic acid (6.8 mmol) and NMM (1.5 mL, 1.5 equiv) were dissolved in DCM (50 mL) and stirred under nitrogen at 0 °C. Isobutylchloroformate (IBCF) (1.5 mL, 1.5 equiv) in DCM (50 mL) was added and the solution was stirred for another 30 min. Piperidine (1.0 mL, 1.5 equiv) was added and the solution was stirred overnight at room temperature. The reaction was quenched with saturated aqueous $NaHCO_3$ and extracted three times with DCM. The combined organic layers were washed with water, saturated aqueous $NaHCO_3$, then brine, dried with $MgSO_4$, filtered, and concentrated under reduced pressure. The crude mixture was purified by column chromatography (30% EtOAc in hexanes) to afford the product (1.1g, 75% yield).

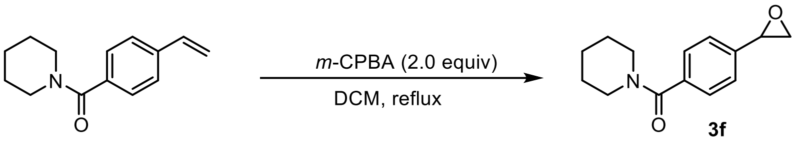

To a flame-dried 50 mL round bottom flask equipped with a stir bar was charged with styrene (1.1 g, 1 equiv) in DCM (20 mL). *m*-Chloroperbenzoic acid (2 equiv) was added and the mixture was refluxed for 2 hours until the starting materiel was fully consumed by TLC. The reaction was quenched with saturated aqueous $NaHCO_3$ and extracted three times with DCM. The combined organic layers were washed with water, saturated aqueous $NaHCO_3$, then brine, dried with $MgSO_4$, filtered, and concentrated under reduced pressure. The crude mixture was purified by column chromatography (30% EtOAc in hexanes) to afford **3f** as a white solid (0.9 g, 82% yield).

Procedure for the preparation of 3h:

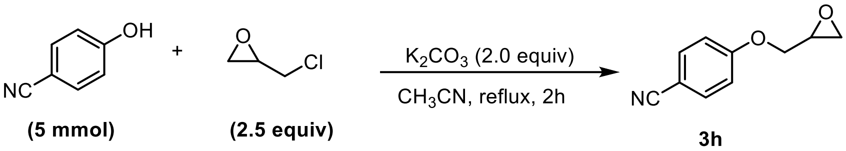

To a flame-dried 50 mL round bottom flask equipped with a stir bar was charged with 4-hydroxybenzonitrile (5.0 mmol), K_2CO_3 (10.0 mmol, 2 equiv) in CH_3CN (20 mL), epichlorohydrin (12.5 mmol, 2.5 equiv) was added and the mixture was refluxed for 2 hours until the starting materiel was fully consumed by TLC. The reaction was quenched with saturated aqueous $NaHCO_3$ and extracted three times with DCM. The combined organic layers were washed with water, saturated aqueous $NaHCO_3$, then brine, dried with $MgSO_4$, filtered, and concentrated under reduced pressure. The crude mixture was purified by column chromatography (30% EtOAc in hexanes) to afford **3h** as a white solid (0.7 g, 80% yield).

Procedure for the preparation of 3i:

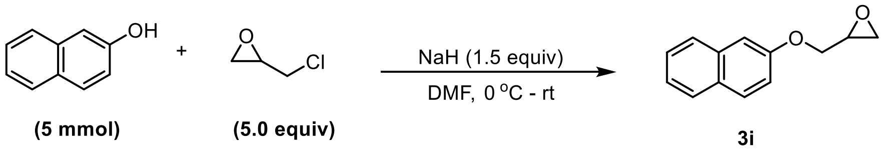

To a flame-dried 5 mL round bottom flask equipped with a stir bar was charged with NaH (60%, 12.5 mmol, 1.5 equiv) and naphthalen-2-ol (5 mmol, 1 equiv). The flask was evacuated and refilled with N_2, and dry DMF (20 mL) were added. Then the epichlorohydrin (25 mmol, 5 equiv) was added at 0 °C and the reaction was allowed to room temperature until complete consumption of

naphthalen-2-ol by TLC. The reaction was diluted with water and extracted three times with Et_2O. The combined organic layers were washed with water, then brine, dried over $MgSO_4$, filtered, and concentrated under reduced pressure. The crude mixture was purified by chromatography on silica gel (Hexane: EtOAc = 10:1) to afford the product **3i** as a white solid (760.9 mg, 76% yield).

Procedure for the preparation of 3k:

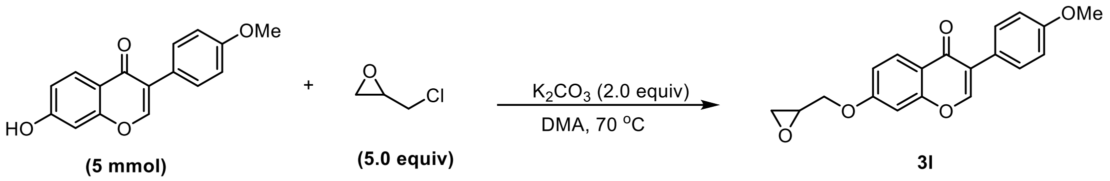

To a flame-dried 50 mL round bottom flask equipped with a stir bar was charged with Formononetin (5.0 mmol), K_2CO_3 (10.0 mmol, 2 equiv) in DMA (20 mL), epichlorohydrin (25 mmol, 5.0 equiv) was added and the mixture was stirred overnight at 70 °C. until the starting materiel was fully consumed by TLC. The reaction was quenched with saturated aqueous $NaHCO_3$ and extracted three times with DCM. The combined organic layers were washed with water, saturated aqueous $NaHCO_3$, then brine, dried with $MgSO_4$, filtered, and concentrated under reduced pressure. The crude mixture was purified by column chromatography (Hexane: EtOAc = 2:1) to afford **3l** as a white solid (1.2 g, 74% yield).

2. Optimization of the reaction conditions of 1a

2.1. Table S1. Optimization of the temperature

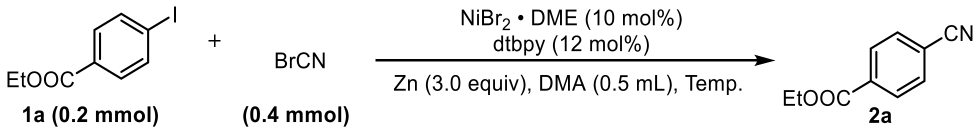

Entry	Temp.	Yield
1	RT	trace
2	40 °C	9%
3	50 °C	34%

Reaction conditions: **1a** (0.2 mmol), BrCN (0.40 mmol, 2.0 M in DMA), $NiBr_2$·DME (10 mol%), dtbpy (12 mol %), Zn (3.0 equiv), DMA (0.5 mL) for 12 h under N_2.

2.2 Table S2. Optimization of the catalyst

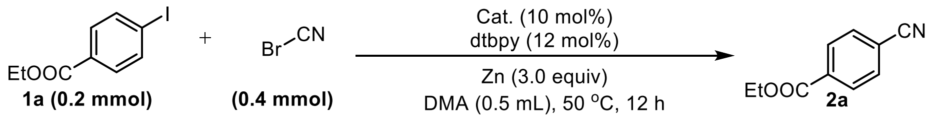

Entry	Cat.	Yield
1	$NiCl_2$	trace
2	NiI_2	31%
3	$NiCl_2$ •1,10-phen	56%
4	$NiCl_2$•$6H_2O$	trace
5	$Ni(acac)_2$	trace
6	$NiBr_2$•DME	34%
7	$NiBr_2$•bipy	38%
8	$NiBr_2$•diglyme	trace
9	$NiCl_2$•DME	trace
10	$NiCl_2$•dppp	55%
11	$NiCl_2$•dppf	27%
12	$Ni(OTf)_2$	trace
13	$FeCl_3$	trace
14	$CoCl_2$	trace
15	$CrCl_2$	trace

Reaction conditions: **1a** (0.2 mmol), BrCN (0.40 mmol, 2.0 M in DMA), Cat. (10 mol%), dtbpy (12 mol%), Zn (3.0 equiv), DMA (0.5 mL), at 50 °C for 12 h under N_2.

2.3 Table S3. Optimization of the solvent

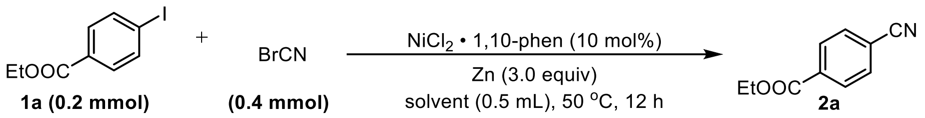

Entry	Solvent	Yield
1	DMF	trace
2	DMSO	trace
3	NMP	trace
4	THF	88%
5	Dioxane	92%
6	DCE	trace
7	CH_3CN	68%

Reaction conditions: **1a** (0.2 mmol), BrCN (0.40 mmol, 2.0 M in solvent), $NiCl_2$·1,10-phen (10 mol%), Zn (3.0 equiv), solvent (0.5 mL), at 50 °C for 12 h under N_2.

3. Optimization of the reaction conditions of 3a

3.1 Table S4. Optimization of the catalyst

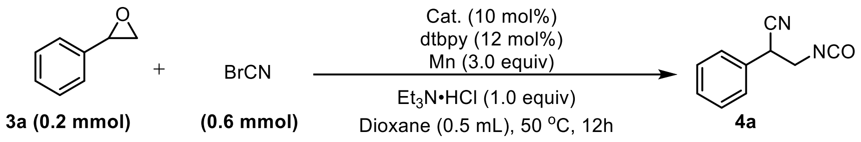

Entry	Cat.	Yield
1	$NiCl_2$	15%
2	$NiBr_2$	18%
3	NiI_2	16%
4	$NiCl_2$•DME	15%
5	$NiCl_2$ •phen	10%
6	$NiCl_2$•dtbpy	10%
7	$NiCl_2$•dppp	12%
8	$Ni(acac)_2$	15%
9	$NiCl_2$•PPh_3	19%
10	$NiCl_2$•dppf	15%
11	$NiBr_2$•bipy	15%
12	$NiBr_2$•diglyme	14%
13	$NiBr_2$•dtbpy	15%
14	$Ni(SO_3CF_3)_2$	12%
15	$CrCl_2$	trace
16	$FeCl_3$	trace
17	$CoCl_2$	trace
18	$CoBr_2$	12%

Reaction conditions: **3a** (0.2 mmol), BrCN (0.6 mmol), Cat. (10 mol%), L1 (12 mol%), Mn (3.0 equiv), $Et_3N{\cdot}HCl$ (1.0 equiv), dioxane (0.5 mL) at 50 °C for 12 h.

3.2 Table S5. Optimization of the ligand

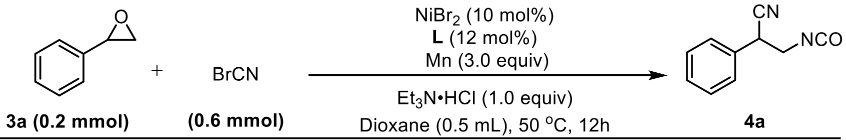

Entry	L	Yield
1	**L1**	<10%
2	**L2**	<10%
3	**L3**	15%
4	**L4**	trace
5	**L5**	11%
6	**L6**	trace
7	**L7**	12%
8	**L8**	trace
9	**L9**	trace
10	**L10**	trace
11	**L11**	18%
12	dppp	12%
13	PPh_3	16%

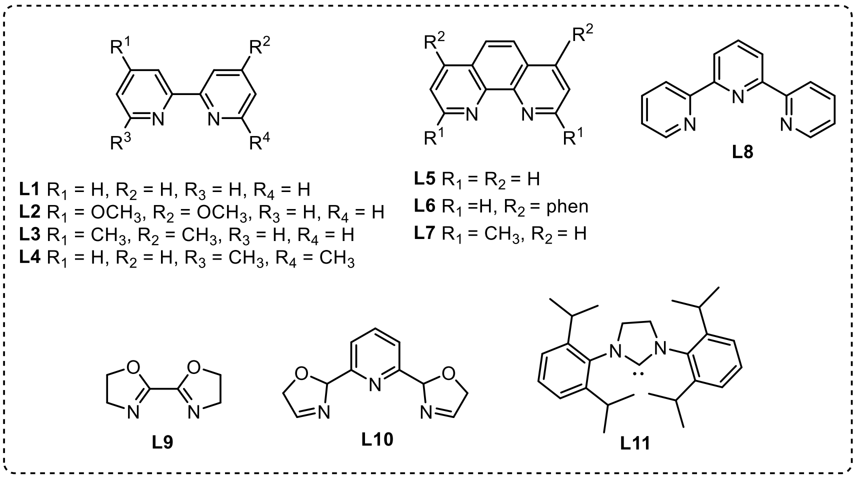

Reaction conditions: **3a** (0.2 mmol), BrCN (0.2 mmol), $NiBr_2$ (10 mol%), L (12 mol%), Mn (3.0 equiv), $Et_3N{\cdot}HCl$ (1.0 equiv), dioxane (0.5 mL) at 50 °C for 12 h.

3.3 Table S6. Optimization of the solvent

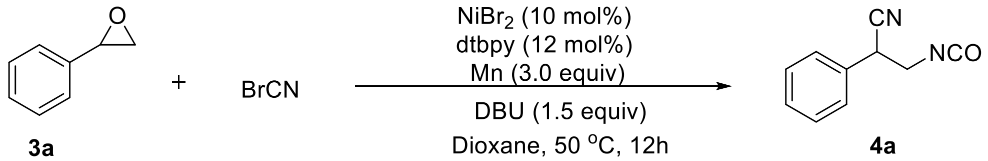

3a **4a**

entry	variation of optimized conditions	yield %
**1**	**none**	**68%**
2	THF	65%
3	DMA	trace
4	DMF	trace
5	DMSO	trace
6	NMP	trace
7	DCE	trace
8	CH_3CN	trace
9[a]	dioxane	trace

[a] with the addition of 1.0 equiv of H_2O.

Reaction conditions: **3a** (0.2 mmol), BrCN (0.2 mmol), $NiBr_2$ (10 mol%), dtbpy (12 mol%), Mn (3.0 equiv), $Et_3N{\cdot}HCl$ (1.0 equiv), solvent (0.5 mL) at 50 °C for 12 h.

3.4 Table S7. Optimization of the additive

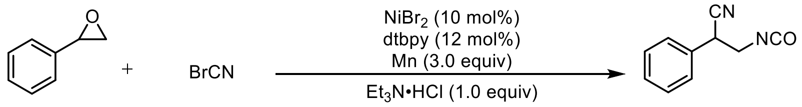

3a (0.2 mmol) **(0.2 mmol)** **4a**

Entry	Add.	Yield
1	LiBr	trace
2	LiI	20%
3	NaI	18%
4	KI	<10%
5	TBAI	22%
6	$MgBr_2$	trace
7	py	<10%
8	TMEDA	trace
9	DABCO	trace
10	DBU	29%
11	Et_3N	22%
12	DIPEA	22%

Reaction conditions: **3a** (0.2 mmol), BrCN (0.2 mmol), $NiBr_2$ (10 mol%), dtbpy (12 mol%), Mn (3.0 equiv), $Et_3N{\cdot}HCl$ (1.0 equiv), additive (1.0 equiv), dioxane (0.5 mL) at 50 °C for 12 h.

3.5 Table S8. Optimization of the equivalent of BrCN

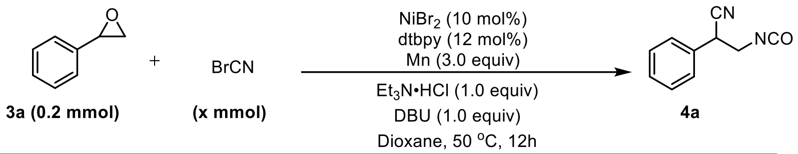

Entry	x	Yield
1	0.4 mmol	35%
2	0.5 mmol	40%
3	0.6 mmol	51%

Reaction conditions: **3a** (0.2 mmol), BrCN (0.2 mmol), $NiBr_2$ (10 mol%), dtbpy (12 mol%), Mn (3.0 equiv), $Et_3N{\cdot}HCl$ (1.0 equiv), DBU (1.0 equiv), dioxane (0.5 mL) at 50 °C for 12 h.

4. Characterization data for products

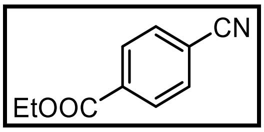

Ethyl 4-cyanobenzoate (2a)[1] Prepared according to the general procedure using ethyl 4-iodobenzoate (52.4 mg, 0.2 mmol) and BrCN (0.4 mmol, 1M in dioxane) at 50 °C. The product was purified by flash column chromatography using 10% EtOAc/hexane to afford nitrile **2a** (32.2 mg, 92%) as white crystal. **^{1}H NMR** (500 MHz, $CDCl_3$) δ 8.14 (d, J = 9.0 Hz, 2H), 7.74 (d, J = 9.0 Hz, 2H), 4.42 (q, J = 7.0 Hz, 2H), 1.41 (t, J = 7.0 Hz, 3H). **^{13}C NMR** (125 MHz, $CDCl_3$) δ 164.96, 134.29, 132.20, 130.07, 118.04, 116.27, 61.83, 14.24. **MS** (EI) *m/z* (relative intensity): 175 (M^+, 20), 130 (100), 102 (50).

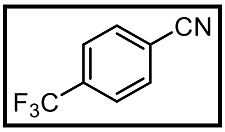

4-(Trifluoromethyl)benzonitrile (2b)[2] Prepared according to the general procedure using 1-iodo-4-(trifluoromethyl)benzene (29 µL, 0.2 mmol) and BrCN (0.4 mmol, 1M in dioxane) at 50 °C. The product was purified by flash column chromatography using 10% EtOAc/hexanes to afford nitrile **2b** (30.4 mg, 89%) as colorless liquid. **^{1}H NMR** (500 MHz, $CDCl_3$) δ 7.82-7.80 (d, J = 8.5 Hz, 2H), 7.77-7.75 (d, J = 8.5 Hz, 2H). **^{13}C NMR** (125 MHz, $CDCl_3$) δ 134.53 (^2J = 32.88 Hz), 132.66, 126.16 (^3J = 3.63 Hz), 123.01 (^1J = 273.42 Hz), 117.41, 116.04. **^{19}F**

NMR (471 MHz, $CDCl_3$) δ -63.54. **MS** (EI) *m/z* (relative intensity): 171 (M^+, 100), 152 (30), 121 (60).

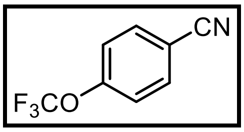

4-(Trifluoromethoxy)benzonitrile (2c)[3] Prepared according to the general procedure using 1-iado-4-(trifluoromethoxy)benzene (31 µL, 0.2 mmol) and BrCN (0.4 mmol, 1M in dioxane) at 50 °C. The product was purified by flash column chromatography using 10% EtOAc/hexanes to afford nitrile **2c** (32.5 mg, 87%) as colorless liquid. ^{1}**H NMR** (500 MHz, $CDCl_3$) δ 7.73 (d, J = 9.0 Hz, 2H), 7.33 (d, J = 9.0 Hz, 2H). ^{13}**C NMR** (125 MHz, $CDCl_3$) δ 152.20, 134.20, 121.22, 120.18 (^{1}J = 260.88 Hz), 117.66, 110.83. ^{19}**F NMR** (471 MHz, $CDCl_3$) δ -57.79. **MS** (EI) *m/z* (relative intensity): 187 (M^+, 100), 90 (25), 69 (75).

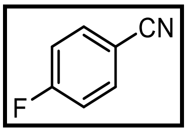

4-Fluorobenzonitrile (2d)[2] Prepared according to the general procedure using 1-fluoro-4-iodobenzene (23 µL, 0.2 mmol) and BrCN (0.4 mmol, 1M in dioxane) at 50 °C. The product was purified by flash column chromatography using 10% EtOAc/hexanes to afford nitrile **2d** (18.4 mg, 76%) as colorless liquid. ^{1}**H NMR** (500 MHz, $CDCl_3$) δ 7.69-7.66 (m, 2H), 7.19-7.15 (m, 2H). ^{13}**C NMR** (125 MHz, $CDCl_3$) δ 165.05 (^{1}J = 255 Hz), 134.71 (^{3}J = 8.8 Hz), 118.04, 116.88 (^{2}J = 23.8 Hz), 108.59 (^{4}J = 3.8 Hz). ^{19}**F NMR** (471 MHz, $CDCl_3$) δ -63.54. **MS** (EI) *m/z* (relative intensity): 121 (M^+, 100), 94 (40), 75 (10).

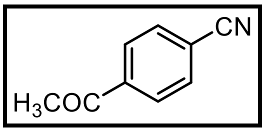

4-Acetylbenzonitrile (2e)[1] Prepared according to the general procedure using 4'-iodoacetophenone (49.2 mg, 0.2 mmol) and BrCN (0.4 mmol, 1M in dioxane) at 50 °C. The product was purified by flash column chromatography using 20% EtOAc/hexanes to afford nitrile **2e** (26.1 mg, 90%) as pure yellow liquid. ^{1}**H NMR** (500 MHz, $CDCl_3$) δ 8.01 (d, J = 9.0 Hz, 2H), 7.74 (d, J = 9.0 Hz, 2H), 2.60

(s, 3H). **^{13}C NMR** (125 MHz, $CDCl_3$) δ 196.44, 139.73, 132.34, 128.54, 117.77, 116.14, 26.61. **MS** (EI) *m/z* (relative intensity): 145 (M^+, 20), 103 (100), 102 (55), 75 (20).

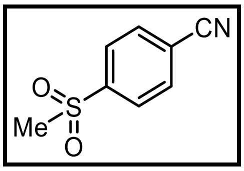

4-(Methylsulfonyl)benzonitrile (2f)[11] Prepared according to the general procedure using 1-bromo-4-(methylsulfonyl)benzene (46.8 mg, 0.2 mmol) and BrCN (0.4 mmol, 1M in dioxane) at 50 °C. The product was purified by flash column chromatography using 30% EtOAc/hexanes to afford nitrile **2f** (19.2 mg, 53%) as white solid. **^{1}H NMR** (400 MHz, $CDCl_3$) δ 8.08 (d, *J* = 8.8 Hz, 2H), 7.89 (d, *J* = 8.8 Hz, 2H), 3.09 (s, 3H). **^{13}C NMR** (100 MHz, $CDCl_3$) δ 144.43, 133.16, 128.15, 117.55, 116.99, 44.16. **HRMS (ESI)** m/z: calcd for $[M+H^+]$ $C_8H_8NO_2S$: 182.0270, found: 182.0273.

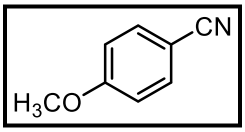

4-Methoxybenzonitrile (2g)[1] Prepared according to the general procedure using 1-iodo-4-(trifluoromethyl)benzene (46.8 mg, 0.2 mmol) and BrCN (0.4 mmol, 1M in dioxane) at 50 °C. The product was purified by flash column chromatography using 20% EtOAc/hexanes to afford nitrile **2g** (22.3 mg, 84%) as white solid. **^{1}H NMR** (500 MHz, $CDCl_3$) δ 7.59 (d, *J* = 9.0 Hz, 2H), 6.95 (d, *J* = 9.0 Hz, 2H), 3.85 (s, 3H). **^{13}C NMR** (125 MHz, $CDCl_3$) δ 162.78, 133.92, 119.18, 114.69, 103.88, 55.49. **MS** (EI) *m/z* (relative intensity): 133 (M^+, 100), 103 (50), 90 (50), 63 (20).

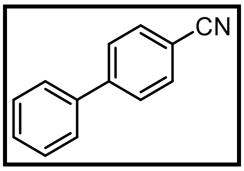

[1,1'-Biphenyl]-4-carbonitrile (2h)[5] Prepared according to the general procedure using 4-iodo-1,1'-biphenyl (56 mg, 0.2 mmol) and BrCN (0.4 mmol, 1M in dioxane) at 50 °C. The product was purified by flash column chromatography using 20% EtOAc/hexanes to afford nitrile **2h** (35.6 mg, 84%) as white solid. **^{1}H NMR** (500 MHz, $CDCl_3$) δ 7.71-7.65 (m, 4H), 7.59-7.57 (m, 2H), 7.50-7.47 (m, 2H), 7.44-7.41 (m, 1H). **^{13}C NMR** (125 MHz, $CDCl_3$) δ 145.37, 138.87, 132.37, 128.92, 128.48,

127.48, 127.00, 118.76, 110.63. **MS** (EI) *m/z* (relative intensity): 179 (M^+, 100), 151 (15), 105 (15), 76 (15).

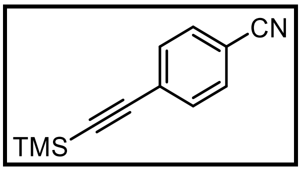

4-[(Trimethylsilyl)ethynyl]benzonitrile (2i)[6] Prepared according to the general procedure using ((4-iodophenyl)ethynyl)trimethylsilane (60 mg, 0.2 mmol) and BrCN (0.4 mmol, 1M in dioxane) at 50 °C. The product was purified by flash column chromatography using 20% EtOAc/hexanes to afford nitrile **2i** (35.5 mg, 89%) as colorless liquid.

^{1}H NMR (500 MHz, $CDCl_3$) δ 7.65-7.63 (m, 2H), 7.19-7.17 (m, 2H), 0.25 (s, 9H). ^{13}C NMR (125 MHz, $CDCl_3$) δ 137.33, 133.39, 122.58, 103.95, 95.85, 94.45, -0.13. **HRMS (ESI)** m/z: calcd for [$M+H^+$] $C_{12}H_{14}NSi$: 200.2890, found: 200.2891.

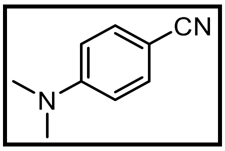

4-(Dimethylamino)benzonitrile (2j)[2] Prepared according to the general procedure using 4-iodo-*N*,*N*-dimethylaniline (49.4 mg, 0.2 mmol) and BrCN (0.4 mmol, 1M in dioxane) at 50 °C. The product was purified by flash column chromatography using 30% EtOAc/hexanes to afford nitrile **2j** (26.9 mg, 92%) as brown solid. **^{1}H NMR** (500 MHz, $CDCl_3$) δ 7.46-7.44 (m, 2H), 6.63-6.62 (m, 2H), 3.03 (s, 6H). **^{13}C NMR** (125 MHz, $CDCl_3$) δ 152.38, 133.29, 120.69, 111.31, 97.17, 39.85. **MS** (EI) *m/z* (relative intensity): 146 (M^+, 100), 129 (20), 102 (20).

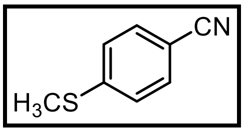

4-(Methylthio)benzonitrile (2k)[4] Prepared according to the general procedure using (4-iodophenyl)(methyl)sulfane (50.0 mg, 0.2 mmol) and BrCN (0.4 mmol, 1M in dioxane) at 50 °C. The product was purified by flash column chromatography using 10% EtOAc/hexanes to afford nitrile **2k** (24.5 mg, 82%) as white solid.

^{1}H NMR (500 MHz, $CDCl_3$) δ 7.58 (d, J = 10.5 Hz, 2H), 6.99 (d, J = 10.5 Hz, 2H), 2.45 (s, 3H). **^{13}C NMR** (125 MHz, $CDCl_3$) δ 138.57, 137.59, 137.57, 128.16, 89.16, 15.61. **MS** (EI) *m/z* (relative intensity): 149 (M^+, 100), 134 (30), 116 (50), 104 (20).

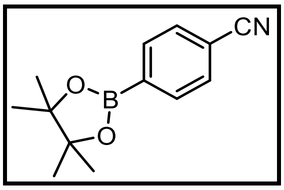

4-(4,4,5,5-Tetramethyl-1,3-dioxolan-2-yl)benzonitrile (2l)[1] repared according to the general procedure using 2-(4-iodophenyl)-4,4,5,5-tetramethyl-1,3,2-dioxaborolane (66.0 mg, 0.2 mmol) and BrCN (0.4 mmol, 1M in dioxane) at 50 °C. The product was purified by flash column chromatography using 20% EtOAc/hexanes to afford nitrile **2l** (38.0 mg, 83%) as white solid. **^{1}H NMR** (500 MHz, $CDCl_3$) δ 7.91-7.64 (m, 2H), 7.62-7.26 (m, 2H), 1.34 (s, 12H). **^{13}C NMR** (125 MHz, $CDCl_3$) δ 135.04, 131.06, 118.80, 114.45, 84.43, 24.80. **HRMS (ESI)** m/z: calcd for $[M+H^+]$ $C_{13}H_{17}NO_2B$ 230.1347, found: 230.1344.

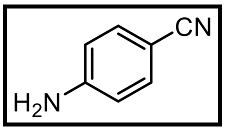

4-Aminobenzonitrile (2m)[2] Prepared according to the general procedure using 4-iodoaniline (43.8 mg, 0.2 mmol) and BrCN (0.4 mmol, 1M in dioxane) at 50 °C. The product was purified by flash column chromatography using 30% EtOAc/hexanes to afford nitrile **2m** (23.6 mg, 85%) as white solid. **^{1}H NMR** (500 MHz, $CDCl_3$) δ 7.41-7.39 (m, 2H), 6.65-6.63 (m, 2H), 4.18 (s, 2H). **^{13}C NMR** (125 MHz, $CDCl_3$) δ 150.37, 133.75, 120.11, 114.37, 100.06. **MS** (EI) *m/z* (relative intensity): 118 (M^+, 100), 91 (45), 64 (15).

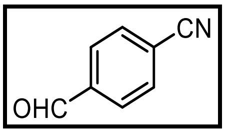

4-Formylbenzonitrile (2n)[1] Prepared according to the general procedure using 4-iodobenzaldehyde (46.4 mg, 0.2 mmol) and BrCN (0.4 mmol, 1M in dioxane) at 50 °C. The product was purified by flash column chromatography using 20% EtOAc/hexanes to afford nitrile **2n** (22 mg, 84%) as white solid. **^{1}H NMR** (500 MHz, $CDCl_3$) δ 10.10 (s, 1H), 8.01-7.99 (m, 2H), 7.86-7.84 (m, 2H). **^{13}C NMR**

(125 MHz, $CDCl_3$) δ 190.58, 138.69, 132.88, 129.87, 117.69, 117.59. **HRMS (ESI)** m/z: calcd for [M+H$^+$] C_8H_6NO 132.0444, found: 132.0443.

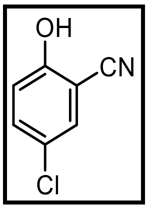

5-Chloro-2-hydroxybenzonitrile (2o)[12] Prepared according to the general procedure using 4-chloro-2-iodophenol (50.9 mg, 0.2 mmol) and BrCN (0.4 mmol, 1M in dioxane) at 50 °C. The product was purified by flash column chromatography using 25% EtOAc/hexanes to afford nitrile **2o** (26.4 mg, 86%) as white solid. **^{1}H NMR** (500 MHz, *d*$_6$-DMSO) δ 11.37 (s, 1H), 7.72-7.71 (m, 1H), 7.52 (dd, *J* = 9.0, 2.5 Hz, 1H), 7.02 (d, *J* = 9.0 Hz, 1H). **^{13}C NMR** (125 MHz, *d*$_6$-DMSO) δ 159.25, 134.63, 132.14, 122.65, 117.90, 115.68, 100.35. **HRMS (ESI)** m/z: calcd for [M+H$^+$] C_7H_5NOCl 154.0054, found: 154.0055.

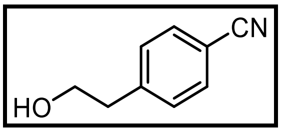

4-(2-Hydroxyethyl)benzonitrile (2p)[7] Prepared according to the general procedure using 4-Iodophenethyl alcohol (49.6 mg, 0.2 mmol) and BrCN (0.4 mmol, 1M in dioxane) at 50 °C. The product was purified by flash column chromatography using 50% EtOAc/hexanes to afford nitrile **2p** (25.3 mg, 86%) as white solid. **^{1}H NMR** (500 MHz, $CDCl_3$) δ 7.59-7.58 (m, 2H), 7.35-7.34 (m, 2H), 3.89 (t, *J* = 6.5 Hz, 2H), 2.93 (t, *J* = 6.5 Hz, 2H), 1.76 (s, 1H). **^{13}C NMR** (125 MHz, $CDCl_3$) δ 144.59, 132.19, 129.79, 118.89, 110.16, 62.80, 39.08. **HRMS (ESI)** m/z: calcd for [M+H$^+$] C_9H_9NO 148.0757, found: 148.0757.

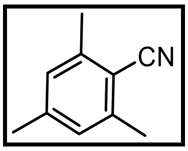

2,4,6-Trimethylbenzonitrile (2q)[5] Prepared according to the general procedure using 2-iodo-1,3,5-trimethylbenzene (49.2 mg, 0.2 mmol) and BrCN (0.4 mmol, 1M in dioxane) at 50 °C. The product was purified by flash column chromatography using 10% EtOAc/hexanes to afford nitrile **2q** (7.6

mg, 26%) as white solid. **^{1}H NMR** (500 MHz, $CDCl_3$) δ 6.93 (s, 2H), 2.48 (s, 6H), 2.32 (s, 3H). **^{13}C NMR** (125 MHz, $CDCl_3$) δ 142.75, 141.92, 128.15, 117.60, 110.27, 21.53, 20.59. **MS** (EI) *m/z* (relative intensity): 145 (M^+, 55), 130 (100), 103 (15), 77 (15).

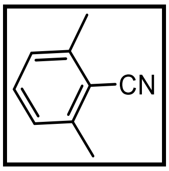

2,4 -Trimethylbenzonitrile (2r)[5] Prepared according to the general procedure using 2-iodo-1,3-dimethylbenzene (46.4 mg, 0.2 mmol) and BrCN (0.4 mmol, 1M in dioxane) at 50 °C. The product was purified by flash column chromatography using 10% EtOAc/hexanes to afford nitrile **2r** (5.2 mg, 20%) as yellow solid. **^{1}H NMR** (500 MHz, $CDCl_3$) δ 7.36-7.32 (m, 1H), 7.13-7.11 (m, 2H), 2.53 (m, 6H). **^{13}C NMR** (125 MHz, $CDCl_3$) δ 142.10, 132.03, 127.26, 117.23, 113.30, 20.73. **MS** (EI) *m/z* (relative intensity): 131 (M^+, 75), 116 (100), 103 (20), 77 (20).

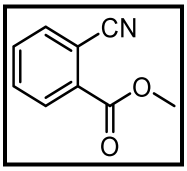

Methyl 2-cyanobenzoate (2s)[13] Prepared according to the general procedure using methyl 2-iodobenzoate (52.4 mg, 0.2 mmol) and BrCN (0.4 mmol, 1M in dioxane) at 50 °C. The product was purified by flash column chromatography using 10% EtOAc/hexanes to afford nitrile **2s** (28.7 mg, 89%) as white solid. **^{1}H NMR** (400 MHz, $CDCl_3$) δ 8.14-8.12 (m, 1H), 7.81-7.78 (m, 1H), 7.69-7.63 (m, 2H), 3.99 (s, 3H). **^{13}C NMR** (100 MHz, $CDCl_3$) δ 164.43, 134.74, 132.64, 132.43, 132.34, 131.11, 117.45, 112.86, 52.78. **HRMS (ESI)** m/z: calcd for [M+H^+] $C_9H_8NO_2$ 162.0550, found: 162.0549.

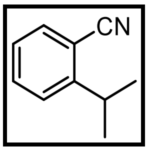

2-Isopropylbenzonitrile (2t)[14] Prepared according to the general procedure using 1-iodo-2-isopropylbenzene (49.3 mg, 0.2 mmol) and BrCN (0.4 mmol, 1M in dioxane) at 50 °C. The product was purified by flash column chromatography using 10% EtOAc/hexanes to afford nitrile **2t** (25.2

mg, 87%) as white solid. ^{1}**H NMR** (400 MHz, $CDCl_3$) δ 7.53-7.51 (m, 1H), 7.49-7.45 (m, 1H), 7.33-7.31 (m, 1H), 7.21-7.17 (m, 1H), 3.36-3.26 (m, 1H), 1.24 (d, *J* = 8.5 Hz, 6H). ^{13}**C NMR** (100 MHz, $CDCl_3$) δ 152.38, 132.94, 132.82, 126.27, 125.90, 118.12, 111.63, 32.38, 23.16. **HRMS (ESI)** m/z: calcd for [M+H$^+$] $C_{10}H_{12}N$ 146.0964, found: 146.0963.

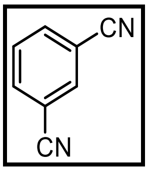

Isophthalonitrile (2u)[1] Prepared according to the general procedure using 3-iodobenzonitrile (45.8 mg, 0.2 mmol) and BrCN (0.4 mmol, 1M in dioxane) at 50 °C. The product was purified by flash column chromatography using 20% EtOAc/hexanes to afford nitrile **2u** (22 mg, 86%) as white solid. ^{1}**H NMR** (500 MHz, $CDCl_3$) δ 7.96 (s, 1H), 7.92-7.90 (m, 2H), 7.68-7.65 (m, 1H). ^{13}**C NMR** (125 MHz, $CDCl_3$) δ 135.96, 135.38, 130.30, 116.56, 114.12. **MS** (EI) *m/z* (relative intensity): 128 (M$^+$, 100), 101 (25), 75 (10), 50 (10).

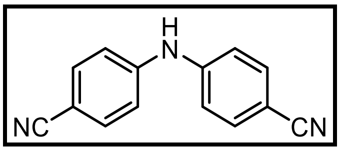

4,4'-Azanediyldibenzonitrile (2v)[10] Prepared according to the general procedure using bis(4-iodophenyl)amine (84.2 mg, 0.2 mmol) and BrCN (0.4 mmol, 1M in dioxane) at 50 °C. The product was purified by flash column chromatography using 20% EtOAc/hexanes to afford nitrile **2v** (34.6 mg, 79%) as white solid. ^{1}**H NMR** (400 MHz, d_6-DMSO) δ 8.70 (s, 1H), 6.96 (m, 4H), 6.51 (m, 4H). ^{13}**C NMR** (100 MHz, d_6-DMSO) δ 146.21, 134.25, 119.91, 117.74, 102.56. **HRMS (ESI)** m/z: calcd for [M+H$^+$] $C_{14}H_9N_3$ 220.0869, found: 220.0870.

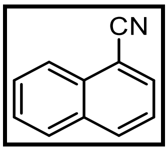

1-Naphthonitrile (2w)[5] Prepared according to the general procedure using 1-naphthonitrile (50.8 mg, 0.2 mmol) and BrCN (0.4 mmol, 1M in dioxane) at 50 °C. The product was purified by flash column chromatography using 20% EtOAc/hexanes to afford nitrile **2w** (26.9 mg, 88%) as red liquid. ^{1}**H NMR**

(500 MHz, $CDCl_3$) δ 8.24 (d, *J* = 7.5 Hz, 1H), 8.08 (d, *J* = 7.5 Hz, 1H), 7.93-7.90 (m, 2H), 7.71-7.68 (m, 1H), 7.64-7.60 (m, 1H), 7.54-7.51 (m, 1H). **^{13}C NMR** (125 MHz, $CDCl_3$) δ 133.24, 132.86, 132.58, 132.29, 128.61, 128.55, 127.50, 125.08, 124.87, 117.78, 110.11. **MS** (EI) *m/z* (relative intensity): 153 (M^+, 100), 126 (25), 76 (10), 63 (10).

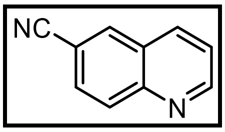

Quinoline-6-carbonitrile (2x)[4] white solid, 86% Prepared according to the general procedure using 6-iodoquinoline (51 mg, 0.2 mmol) and BrCN (0.4 mmol, 1M in dioxane) at 50 °C. The product was purified by flash column chromatography using 40% EtOAc/hexanes to afford nitrile **2x** (26.4 mg, 86%) as white solid. **^{1}H NMR** (500 MHz, $CDCl_3$) δ 9.07 (s, 1H), 8.27-8.23 (m, 3H), 7.89-7.87 (m, 1H), 7.58-7.56 (m, 1H). **^{13}C NMR** (125 MHz, $CDCl_3$) δ 153.13, 148.75, 136.83, 134.16, 130.86, 130.40, 127.62, 122.83, 118.43, 110.63. **MS** (EI) *m/z* (relative intensity): 154 (M^+, 100), 127 (30), 100 (10), 75 (10).

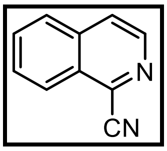

Isoquinoline-1-carbonitrile (2y)[15] Prepared according to the general procedure using 1-bromoisoquinoline (41.6 mg, 0.2 mmol) and BrCN (0.4 mmol, 1M in dioxane) at 50 °C. The product was purified by flash column chromatography using 40% EtOAc/hexanes to afford nitrile **2y** (24.6 mg, 80%) as white solid. **^{1}H NMR** (500 MHz, $CDCl_3$) δ 8.65 (d, *J* = 5.5 Hz, 1H), 8.35-8.33 (m, 1H), 7.96-7.94 (m, 1H), 7.91, 7.91-7.90 (m, 1H), 7.85-7.78 (m, 2H). **^{13}C NMR** (125 MHz, $CDCl_3$) δ 143.24, 135.87, 134.76, 131.71, 129.84, 129.32, 127.28, 125.28, 124.41, 115.79. **HRMS (ESI)** m/z: calcd for $[M+H^+]$ $C_{10}H_7N_2$ 155.0604, found: 155.0604.

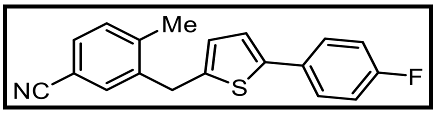

3-{[5-(4-Fluorophenyl)thiophen-2-yl]methyl}-4-methylbenzonitrile (2z)[9] Prepared according to the general procedure using 2-(4-fluorophenyl)-5-(5-iodo-2-methylbenzyl)thiophene (81.6 mg, 0.2 mmol) and BrCN (0.4 mmol, 1M in dioxane) at 50 °C. The product was purified by flash column chromatography using 20% EtOAc/hexanes to afford nitrile **2z** (54.7 mg, 89%) as white solid. **^{1}H**

NMR (500 MHz, $CDCl_3$) δ 7.40-7.35 (m, 4H), 7.18-7.16 (m, 1H), 6.96-6.92 (m, 3H), 6.59-6.58 (m, 1H), 4.03 (s, 2H), 2.29 (s, 3H). **^{13}C NMR** (125 MHz, $CDCl_3$) δ 162.11 (^1J = 245.75 Hz), 142.21, 142.12, 141.01, 139.59, 132.60, 131.12, 130.45 (^4J = 3.50 Hz), 130.43, 127.11 (^3J = 7.88 Hz), 126.53, 122.75, 118.98, 115.70 (^2J = 21.75 Hz), 109.95, 33.51, 19.81. **MS** (EI) *m/z* (relative intensity): 307 (M^+, 90), 191 (30), 178 (100).

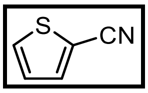

Thiophene-2-carbonitrile (2a')[5] Prepared according to the general procedure using 2-iodothiophene (22 μL, 0.2 mmol) and BrCN (0.4 mmol, 1M in dioxane) at 50 ^{o}C. The product was purified by flash column chromatography using 10% EtOAc/hexanes to afford nitrile **2a'** (18.36 mg, 85%) as yellow liquid. **^{1}H NMR** (500 MHz, $CDCl_3$) δ 7.60-7.59 (m, 2H), 7.12-7.10 (m, 1H). **^{13}C NMR** (125 MHz, $CDCl_3$) δ 137.25, 132.51, 127.49, 114.05, 109.51. **MS** (EI) *m/z* (relative intensity): 109 (M^+, 100), 70 (20), 58 (40), 45 (40).

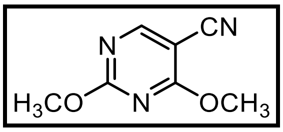

2,4-Dimethoxypyrimidine-5-carbonitrile (2b') Prepared according to the general procedure using 5-iodo-2,4-dimethoxypyrimidine (53.2 mg, 0.2 mmol) and BrCN (0.4 mmol, 1M in dioxane) at 50 ^{o}C. The product was purified by flash column chromatography using 20% EtOAc/hexanes to afford nitrile **2b'** (29.0 mg, 88%) as white solid. **^{1}H NMR** (500 MHz, $CDCl_3$) δ 8.52 (s, 1H), 4.09 (s, 3H), 4.05 (s, 3H). **^{13}C NMR** (125 MHz, $CDCl_3$) δ 170.96, 166.48, 163.44, 113.48, 90.07, 55.81, 55.11. **HRMS (ESI)** m/z: calcd for $[M+H^+]$ $C_7H_7N_3O_2$ 166.0611, found: 166.0614.

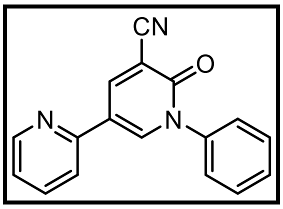

6'-Oxo-1'-phenyl-1',6'-dihydro-[2,3'-bipyridine]-5'-carbonitrile (2c') Prepared according to the general procedure using 5'-bromo-1'-phenyl-[2,3'-bipyridin]-6'(1'*H*)-one (65.4 mg, 0.2 mmol) and BrCN (0.4 mmol, 1M in dioxane) at 50 °C. The product was purified by flash column chromatography using 20% EtOAc/hexanes to afford nitrile **2c'** (33.9 mg, 62%) as white solid. **^{1}H NMR** (400 MHz, $CDCl_3$) δ 8.54-8.52 (m, 1H), 8.49-8.48 (m, 1H), 8.40-8.40 (m, 1H) 7.73-7.69 (m, 1H), 7.50-7.46 (m, 3H), 7.38-7.37 (m, 2H), 7.21-7.18 (m, 2H). **^{13}C NMR** (100 MHz, $CDCl_3$) δ 158.91, 151.18, 149.96, 145.66, 141.92, 139.64, 137.36, 129.67, 129.56, 126.25, 122.75, 118.37, 118.23, 115.32, 106.28. **HRMS (ESI)** m/z: calcd for [M+H$^+$] $C_{17}H_{12}N_3O$ 274.0975, found: 274.0973.

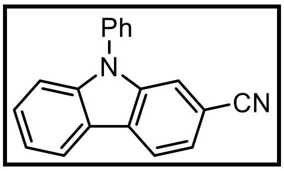

9-Phenyl-9*H*-carbazole-2-carbonitrile (2d')[8] Prepared according to the general procedure using 2-bromo-9-phenyl-9H-carbazole (64.4 mg, 0.2 mmol) and BrCN (0.4 mmol, 1M in dioxane) at 50 °C. The product was purified by flash column chromatography using 30% EtOAc/hexanes to afford nitrile **2d'** (41.8 mg, 78%) as white solid. **^{1}H NMR** (500 MHz, $CDCl_3$) δ 8.44 (m, 1H), 8.16 (d, *J* = 7.5 Hz, 1H), 7.66-7.63 (m, 3H), 7.56-7.48 (m, 4H), 7.4-7.36 (m, 3H). **^{13}C NMR** (125 MHz, $CDCl_3$) δ 142.50, 141.57, 136.36, 130.13, 129.11, 128.38, 127.34, 127.05, 125.20, 123.41, 122.11, 121.18, 120.59, 120.36, 110.44, 110.32, 102.58. **MS** (EI) *m/z* (relative intensity): 268 (M$^+$, 100), 134 (10), 120 (10).

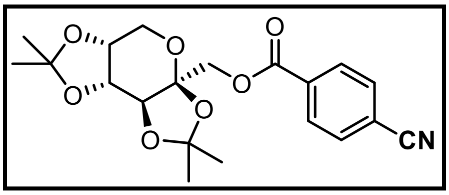

Levulose derivative (2e') Prepared according to the general procedure using ((3a*S*,5a*R*,8a*R*,8b*S*)-2,2,7,7-tetramethyltetrahydro-3a*H*-bis([1,3]dioxolo)[4,5-*b*:4',5'-*d*]pyran-3a-yl)methyl 4-iodobenzoate (98.1 mg, 0.2 mmol) and BrCN (0.4 mmol, 1M in dioxane) at 50 °C. The product was purified by flash column chromatography using 40% EtOAc/hexanes to afford nitrile **2e'** (55.3 mg, 71%) as colorless crystal. **^{1}H NMR** (500 MHz, $CDCl_3$) δ 8.17-8.15 (m, 2H), 7.74-7.72 (m, 2H), 4.69 (d, *J* = 11.5 Hz, 1H), 4.62(dd, *J* = 8.0, 3.0 Hz, 1H), 4.39 (d, *J* = 3.0 Hz, 1H), 4.35 (d, *J* = 11.5 Hz, 1H), 4.25 (d, *J* = 8.0 Hz, 1H), 3.94 (d, *J* = 13.5 Hz, 1H), 3.79 (d, *J* = 15.0 Hz, 1H), 1.52 (s, 3H), 1.42

(s, 3H), 1.32 (s, 6H). **^{13}C NMR** (125 MHz, $CDCl_3$) δ 164.30, 133.64, 132.17, 130.18, 117.80, 116.50, 109.06, 108.80, 101.33, 70.60, 70.58, 69.92, 66.11, 61.28, 26.37, 25.78, 25.32, 23.89. **HRMS (ESI)** m/z: calcd for [M+H$^+$] $C_{20}H_{24}NO_7$ 390.1547, found: 390.1548.

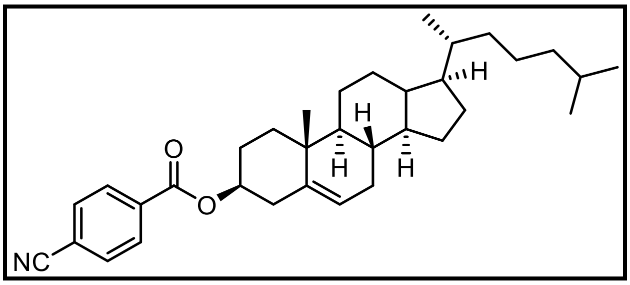

Cholesterol derivative (2f') Prepared according to the general procedure using (3*S*,8*S*,9*S*,10*R*,14*R*,17*R*)-10-methyl-17-((*R*)-6-methylheptan-2-yl)-2,3,4,7,8,9,10,11,12,13,14,15,16,17-tetradecahydro-1*H*-cyclopenta[*a*]phenanthren-3-yl 4-iodobenzoate (120.5 mg, 0.2 mmol) and BrCN (0.4 mmol, 1M in dioxane) at 50 °C. The product was purified by flash column chromatography using 50% EtOAc/hexanes to afford nitrile **2f'** (32.1 mg, 32%) as white solid. **^{1}H NMR** (500 MHz, $CDCl_3$) δ 8.14-8.12 (m, 2H), 7.73-7.72 (m, 2H), 5.42-5.41 (m, 1H), 4.90-4.84 (m, 1H), 2.47-2.45 (m, 2H), 2.0-1.97 (m, 3H), 1.93-1.91 (m, 1H), 1.87-1.81 (m, 1H), 1.76-1.72 (m, 1H), 1.59-1.45 (m, 7H), 1.28-1.23 (m, 5H), 1.11-1.06 (m, 3H), 1.04-0.97 (m, 3H), 0.92 (d, J = 6.5 Hz, 3H), 0.87 (dd, J_1 = 6.5 Hz, J_2= 2.5 Hz, 9H), 0.68 (s, 3H). **^{13}C NMR** (125 MHz, $CDCl_3$) δ 164.20, 139.18, 134.57, 132.06, 130.00, 123.08, 117.96, 116.13, 75.53, 56.62, 56.09, 49.97, 42.26, 39.47, 38.05, 36.91, 36.56, 36.14, 35.75, 31.89, 29.68, 28.19, 27.96, 27.75, 24.24, 23.80, 22.78, 22.53, 21.00, 19.30, 18.68, 11.81. **HRMS (ESI)** m/z: calcd for [M+H$^+$] $C_{34}H_{48}NO_2$ 502.3680, found: 502.3684.

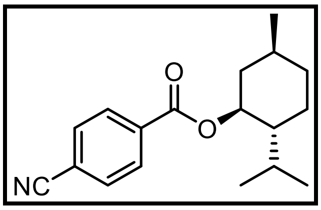

DL-Menthol derivative (2g') Prepared according to the general procedure using (1*S*,2*R*,5*S*)-2-isopropyl-5-methylcyclohexyl 4-iodobenzoate (77.2 mg, 0.2 mmol) and BrCN (0.4 mmol, 1M in dioxane) at 50 °C. The product was purified by flash column chromatography using 20% EtOAc/hexanes to afford nitrile **2g'** (46.2 mg, 81%) as white solid. **^{1}H NMR** (500 MHz, $CDCl_3$) δ

8.14-8.12 (m, 2H), 7.74-7.73 (m, 2H), 4.98-4.92 (m, 1H), 2.13-2.09 (m, 1H), 1.93-1.89 (m, 1H), 1.75-1.71 (m, 2H), 1.59-1.52 (m, 2H), 1.17-1.07 (m, 2H), 0.96-0.91 (m, 7H), 0.79 (d, *J* = 7.0 Hz, 3H). **^{13}C NMR** (125 MHz, $CDCl_3$) δ 164.40, 134.61, 132.13, 130.03, 118.03, 116.11, 75.90, 47.15, 40.80, 34.15, 31.40, 26.51, 23.53, 21.96, 20.69, 16.43. **HRMS (ESI)** m/z: calcd for [M+H$^+$] $C_{18}H_{24}NO_2$ 286.1802, found: 286.1802.

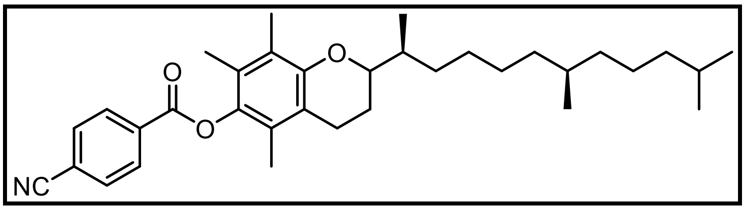

Vitamin E derivative (2h') Prepared according to the general procedure using 2-((2*S*,7*S*)-7,11-dimethyldodecan-2-yl)-5,7,8-trimethylchroman-6-yl 4-iodobenzoate (123.7 mg, 0.2 mmol) and BrCN (0.4 mmol, 1M in dioxane) at 50 ^{o}C. The product was purified by flash column chromatography using 40% EtOAc/hexanes to afford nitrile **2h'** (67.3 mg, 65%) as yellow solid. **^{1}H NMR** (500 MHz, $CDCl_3$) δ 8.37-8.35 (m, 2H), 7.84-7.82 (m, 2H), 2.65 (t, *J* = 7.0 Hz, 2H), 2.14 (s, 3H), 2.06 (s, 3H), 2.02 (s, 3H), 1.87-1.84 (m, 1H), 1.81-1.78 (m, 1H), 1.58-1.54 (m, 2H), 1.44-1.40 (m, 2H), 1.32-1.27 (m, 9H), 1.17-1.13 (m, 3H), 1.10-1.07 (m, 2H), 0.89-0.86 (m, 12H). **^{13}C NMR** (125 MHz, $CDCl_3$) δ 163.54, 149.70, 140.29, 133.37, 132.37, 130.54, 126.52, 124.80, 123.29, 117.86, 117.58, 116.79, 75.14, 39.31, 37.40, 37.38, 37.23, 32.74, 27.92, 24.76, 24.40, 24.14, 23.62, 22.68, 22.59, 20.96, 20.57, 19.71, 19.62, 13.01, 12.16, 11.82. **HRMS (ESI)** m/z: calcd for [M+H$^+$] $C_{34}H_{48}NO_3$ 518.3629, found: 518.3626.

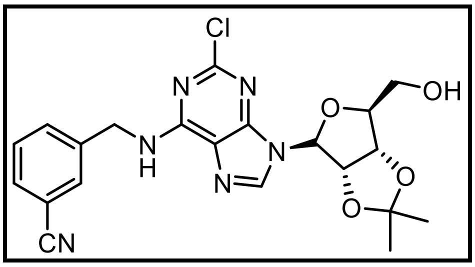

CF102 intermediate (2i') Prepared according to the general procedure using ((3a*S*,4*S*,6*R*,6a*S*)-6-(2-chloro-6-((3-iodobenzyl)amino)-9*H*-purin-9-yl)-2,2-dimethyltetrahydrofuro[3,4-*d*][1,3]dioxol-4-yl)methanol (111.6 mg, 0.2 mmol) and BrCN (0.4 mmol, 1M in dioxane) at 50 ^{o}C. The product was purified by flash column chromatography using 100% EtOAc/hexanes to afford nitrile **2i'** (68.5

mg, 75%) as yellow solid. **^{1}H NMR** (400 MHz, $CDCl_3$) δ 7.71 (s, 1H), 7.62 (s, 1H), 7.58-7.53 (m, 2H), 7.43-7.39 (m, 1H), 7.00 (s, 1H), 5.80 (d, *J* = 4.8 Hz, 1H), 5.55-5.52 (m, 1H), 5.17-5.07 (m, 2H), 4.82 (s, 1H), 4.47 (s, 1H), 4.12 (q, *J* = 7.6 Hz, 1H), 3.96-3.93 (m, 1H), 3.81 (t, *J* = 10.8 Hz, 1H), 1.61 (s, 3H), 1.34 (s, 3H). **^{13}C NMR** (100 MHz, $CDCl_3$) δ 154.96, 148.76, 140.07, 132.16, 131.21, 131.08, 129.41, 118.47, 114.12, 112.64, 93.59, 86.02, 82.86, 81.38, 63.18, 60.32, 43.57, 27.48, 25.14, 20.98, 14.10. **HRMS (ESI)** m/z: calcd for [M+H$^+$] $C_{21}H_{22}N_6O_4Cl$ 457.1386, found: 457.1383.

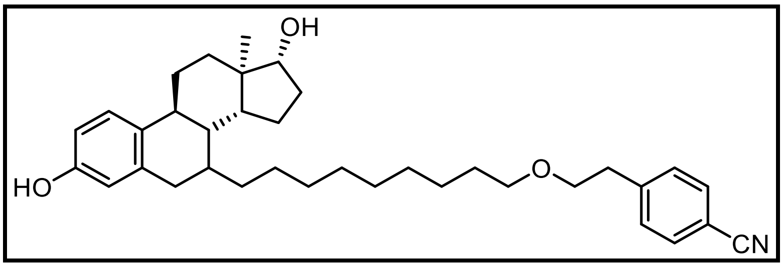

Fulvestrant intermediate (2j') Prepared according to the general procedure using (8*S*,9*S*,13*R*,14*R*,17*R*)-7-(9-(4-iodophenethoxy)nonyl)-13-methyl-7,8,9,11,12,13,14,15,16,17-decahydro-6*H*-cyclopenta[*a*]phenanthrene-3,17-diol (128.9 mg, 0.2 mmol) and BrCN (0.4 mmol, 1M in dioxane) at 50 °C. The product was purified by flash column chromatography using 50% EtOAc/hexanes to afford nitrile **2j'** (72.9 mg, 67%) as yellow solid. **^{1}H NMR** (400 MHz, $CDCl_3$) δ 7.53 (m, 2H), 7.40 (m, 2H), 7.17 (s, 2H), 7.09 (d, *J* = 8.0 Hz, 1H), 6.70-6.68 (m, 1H), 6.63-6.60 (m, 2H), 5.81(d, *J* = 10.0 Hz, 1H), 5.37(d, *J* = 12.0 Hz, 1H), 4.62-4.58 (m, 1H), 4.19-4.13 (m, 1H), 4.02-3.97 (m, 1H), 2.78-2.67 (m, 2H), 1.96 (s, 3H), 1.47 (m, 4H), 1.31-1.10 (m, 22H), 0.77 (s, 3H). **^{13}C NMR** (100 MHz, $CDCl_3$) δ 171.22, 157.03, 141.89, 136.68, 135.36, 132.39, 127.43, 126.73, 120.79, 117.71, 116.67, 82.75, 70.24, 46.70, 43.42, 42.79, 40.47, 37.45, 35.70, 33.77, 31.43, 30.19, 28.94, 28.82, 28.13, 27.67, 27.42, 27.16, 24.93, 24.42, 22.84, 22.67, 21.18, 12.33. **HRMS (ESI)** m/z: calcd for [M+H$^+$] $C_{36}H_{50}NO_3$ 544.3785, found: 544.3786.

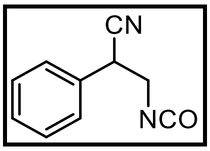

3-Isocyanato-3-phenylpropanenitrile (4a) Prepared according to the general procedure using 2-phenyloxirane (19 µL, 0.2 mmol) and BrCN (0.6 mmol, 1M in dioxane) at 50 °C. The product was

purified by flash column chromatography using 10% EtOAc/hexanes to afford nitrile **4a** (23.0 mg, 67%) as yellow liquid. **^{1}H NMR** (500 MHz, $CDCl_3$) δ 7.42-7.35 (m, 3H), 7.23-7.22 (m, 2H), 5.42-5.39 (m, 1H), 4.84-4.80 (m, 1H), 4.33-4.30 (m, 1H). **^{13}C NMR** (125 MHz, $CDCl_3$) δ 142.17, 139.07, 129.15, 128.55, 126.54, 108.87, 75.46, 70.31. **HRMS (ESI)** m/z: calcd for $[M+H^+]$ $C_{10}H_9N_2O$ 173.0709, found: 173.0710.

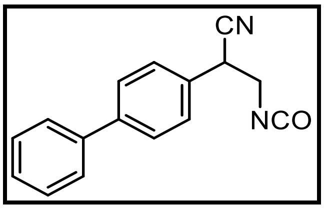

3-([1,1'-Biphenyl]-4-yl)-3-isocyanatopropanenitrile (4b) Prepared according to the general procedure using 2-([1,1'-biphenyl]-4-yl)oxirane (39.2 mg, 0.2 mmol) and BrCN (0.6 mmol, 1M in dioxane) at 50 °C. The product was purified by flash column chromatography using 20% EtOAc/hexanes to afford nitrile **4b** (31.7 mg, 64%) as white solid. **^{1}H NMR** (500 MHz, $CDCl_3$) δ 7.64-7.58 (m, 4H), 7.48-7.45 (m, 2H), 7.39-7.36 (m, 1H), 7.31-7.30 (m, 2H), 5.47-5.44 (m, 1H), 4.87-4.83 (m, 1H), 4.38-4.34 (m, 1H). **^{13}C NMR** (125 MHz, $CDCl_3$) δ 142.15, 141.49, 140.26, 137.92, 128.83, 127.79, 127.60, 127.07, 126.95, 108.82, 75.35, 70.00. **HRMS (ESI)** m/z: calcd for $[M+H^+]$ $C_{16}H_{13}N_2O$ 249.1022, found: 249.1024.

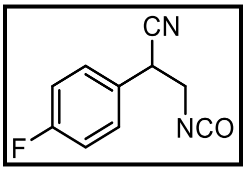

3-(4-Fluorophenyl)-3-isocyanatopropanenitrile (4c) Prepared according to the general procedure using 2-(4-fluorophenyl)oxirane (27.6 mg, 0.2 mmol) and BrCN (0.6 mmol, 1M in dioxane) at 50 °C. The product was purified by flash column chromatography using 10% EtOAc/hexanes to afford nitrile **4c** (28.9 mg, 76%) as yellow liquid. **^{1}H NMR** (500 MHz, $CDCl_3$) δ7.21-7.18 (m, 2H), 7.10-7.06 (m, 2H), 5.41-5.37 (m, 1H), 4.84-4.80 (m, 1H), 4.29-4.25 (m, 1H). **^{13}C NMR** (125 MHz) δ 162.60 (^1J = 246.25 Hz), 142.19, 134.83, 128.30, 127.68 (^3J = 8.75 Hz), 115.94, 115.52 (^2J = 21.25 Hz), 108.71, 75.36, 69.55. **HRMS (ESI)** m/z: calcd for $[M+H^+]$ $C_{10}H_8FN_2O$ 191.0615, found: 191.0616.

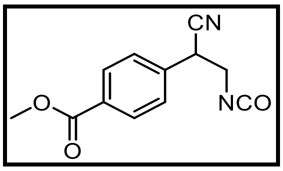

Methyl 4-(2-cyano-1-isocyanatoethyl)benzoate (4d) Prepared according to the general procedure using methyl 4-(oxiran-2-yl)benzoate (35.6 mg, 0.2 mmol) and BrCN (0.6 mmol, 1M in dioxane) at 50 ^oC. The product was purified by flash column chromatography using 20% EtOAc/hexanes to afford nitrile **4d** (33.1 mg, 72%) as white solid. **^{1}H NMR** (500 MHz, $CDCl_3$) δ 7.97-7.89 (m, 2H), 7.37-7.35 (m, 1H), 7.27-7.19 (m, 1H), 3.80 (m, 3H), 3.54-3.42 (m, 1H), 2.94-2.92 (m, 2H). **^{13}C NMR** (125 MHz, $CDCl_3$) δ 165.97, 146.07, 143.96, 130.30, 126.56, 126.10, 108.73, 75.20, 69.80, 39.19. **HRMS (ESI)** m/z: calcd for [M+H$^+$] $C_{12}H_{11}N_2O_3$ 231.0764, found: 231.0765.

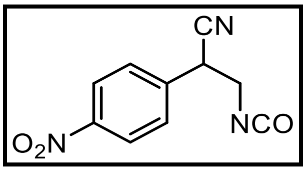

3-Isocyanato-3-(4-nitrophenyl)propanenitrile (4e) Prepared according to the general procedure using 2-(4-nitrophenyl)oxirane (33 mg, 0.2 mmol) and BrCN (0.6 mmol, 1M in dioxane) at 50 ^oC. The product was purified by flash column chromatography using 40% EtOAc/hexanes to afford nitrile **4e** (23.4 mg, 54%) as yellow solid. **^{1}H NMR** (500 MHz, $CDCl_3$) δ 8.20-8.18 (m, 2H), 7.57-7.55 (m, 2H), 5.04-5.02 (m, 1H), 3.66-3.63 (m, 1H), 3.54-3.50 (m, 1H). **^{13}C NMR** (125 MHz, $CDCl_3$) δ 126.92, 126.37, 124.31, 123.65, 121.26, 108.33, 81.01, 72.49. **HRMS (ESI)** m/z: calcd for [M+H$^+$] $C_{10}H_8N_3O_3$ 218.0560, found: 218.0561.

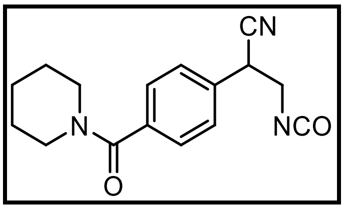

3-Isocyanato-3-(4-(piperidine-1-carbonyl)phenyl)propanenitrile (4f) Prepared according to the general procedure using (4-(oxiran-2-yl)phenyl)(piperidin-1-yl)methanone (46.2 mg, 0.2 mmol) and BrCN (0.6 mmol, 1M in dioxane) at 50 ^oC. The product was purified by flash column chromatography using 40% EtOAc/hexanes to afford nitrile **4f** (35.1 mg, 62%) as white solid. **^{1}H NMR** (500 MHz, $CDCl_3$) δ 7.41-7.24 (m, 4H), 4.87-4.84 (m, 1H), 3.69 (s, 2H), 3.55-3.49 (m, 2H), 3.31

(s, 2H), 1.69-1.50 (m, 6H). **^{13}C NMR** (125 MHz, $CDCl_3$) δ 169.81, 140.08, 136.41, 127.38, 127.35, 126.72, 108.54, 75.13, 69.68, 39.26, 24.28, 20.85. **HRMS (ESI)** m/z: calcd for [M+H$^+$] $C_{16}H_{18}N_3O_2$ 284.1394, found: 284.1395.

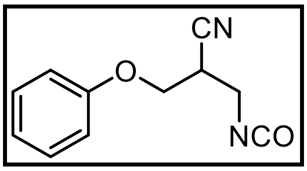

3-Isocyanato-4-phenoxybutanenitrile (4g) Prepared according to the general procedure using 2-(phenoxymethyl)oxirane (30.0 mg, 0.2 mmol) and BrCN (0.6 mmol, 1M in dioxane) at 50 ^{o}C. The product was purified by flash column chromatography using 10% EtOAc/hexanes to afford nitrile **4g** (26.7 mg, 66%) as white solid. **^{1}H NMR** (500 MHz, $CDCl_3$) δ 7.33-7.29 (m, 2H), 7.03-7.00 (m, 1H), 6.91-6.89 (m, 2H), 5.09-5.06 (m, 1H), 4.22-4.16 (m, 2H), 4.10-4.05 (m, 2H). **^{13}C NMR** (125 MHz, $CDCl_3$) δ 157.94, 141.59, 129.69, 121.87, 118.14, 114.60, 79.19, 67.77, 57.06. **HRMS (ESI)** m/z: calcd for [M+H$^+$] $C_{11}H_{11}N_2O_2$ 203.0815, found: 203.0817.

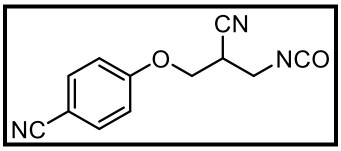

4-(3-Cyano-2-isocyanatopropoxy)benzonitrile (4h) Prepared according to the general procedure using 4-(oxiran-2-ylmethoxy)benzonitrile (35.0 mg, 0.2 mmol) and BrCN (0.6 mmol, 1M in dioxane) at 50 ^{o}C. The product was purified by flash column chromatography using 40% EtOAc/hexanes to afford nitrile **4h** (30.9 mg, 68%) as white solid. **^{1}H NMR** (500 MHz, $CDCl_3$) δ 7.60-7.58 (m, 2H), 6.97-6.95 (m, 2H), 5.11-5.09 (m, 1H), 4.25-4.19 (m, 2H), 4.12-4.03 (m, 2H). **^{13}C NMR** (125 MHz, $CDCl_3$) δ 161.10, 141.42, 134.18, 118.85, 115.34, 108.68, 105.22, 78.74, 68.02, 56.91. **HRMS (ESI)** m/z: calcd for [M+H$^+$] $C_{12}H_{10}N_3O_2$ 228.0768, found: 228.0769.

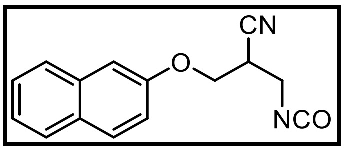

3-Isocyanato-4-(naphthalen-2-yloxy)butanenitrile (4i) Prepared according to the general procedure using 2-((naphthalen-2-yloxy)methyl)oxirane (40 mg, 0.2 mmol) and BrCN (0.6 mmol, 1M in dioxane) at 50 °C. The product was purified by flash column chromatography using 20% EtOAc/hexanes to afford nitrile **4i** (35.3 mg, 70%) as yellow liquid. **^{1}H NMR** (500 MHz, $CDCl_3$) δ 7.81-7.74 (m, 3H), 7.50-7.47 (m, 1H), 7.41-7.39 (m, 1H), 7.17-7.15 (m, 1H), 7.11-7.10 (m, 1H), 5.09-5.06 (m, 1H), 4.24-4.05 (m, 4H). **^{13}C NMR** (125 MHz, $CDCl_3$) δ 155.92, 141.59, 134.29, 129.84, 129.39, 127.78, 126.86, 126.73, 124.25, 118.53, 108.92, 106.98, 79.21, 67.83, 57.06. **HRMS (ESI)** m/z: calcd for [M+H$^+$] $C_{15}H_{13}N_2O_2$ 253.0972, found: 253.0973.

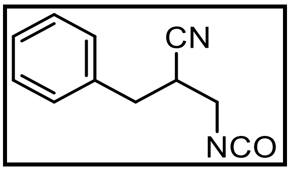

3-Isocyanato-4-phenylbutanenitrile (4j) Prepared according to the general procedure using 2-benzyloxirane (26.8 mg, 0.2 mmol) and BrCN (0.6 mmol, 1M in dioxane) at 50 °C. The product was purified by flash column chromatography using 10% EtOAc/hexanes to afford nitrile **4j** (29.0 mg, 78%) as white solid. **^{1}H NMR** (500 MHz, $CDCl_3$) δ 7.37-7.28 (m, 3H), 7.22-7.20 (m, 2H), 5.02-4.98 (m, 1H), 4.08-4.03 (m, 1H), 3.80-3.75 (m, 1H), 3.07-3.03 (m, 2H). **^{13}C NMR** (125 MHz, $CDCl_3$) δ 141.43, 134.77, 129.27, 128.80, 127.29, 108.89, 81.91, 59.27, 30.55. **HRMS (ESI)** m/z: calcd for [M+H$^+$] $C_{11}H_{11}N_2O$ 187.0866, found: 187.0868.

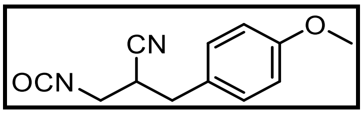

3-Isocyanato-4-(4-methoxyphenyl)butanenitrile (4k) Prepared according to the general procedure using 2-(4-methoxybenzyl)oxirane (32.8 mg, 0.2 mmol) and BrCN (0.6 mmol, 1M in dioxane) at 50 °C. The product was purified by flash column chromatography using 10% EtOAc/hexanes to afford nitrile **4k** (33.3 mg, 77%) as white solid. **^{1}H NMR** (500 MHz, $CDCl_3$) δ 7.12-7.11 (m, 2H), 6.88-6.86 (m, 2H), 4.97- 4.94 (m, 1H), 4.06-4.01 (m, 1H), 3.80 (s, 3H), 3.78-3.73 (m, 1H), 3.00-2.85 (m, 2H). **^{13}C NMR** (125 MHz, $CDCl_3$) δ 158.87, 141.54, 130.35, 126.70, 114.28, 109.00, 82.18, 59.23, 55.29, 39.63. **HRMS (ESI)** m/z: calcd for [M+H$^+$] $C_{12}H_{13}N_2O_2$ 217.0972, found: 217.0973.

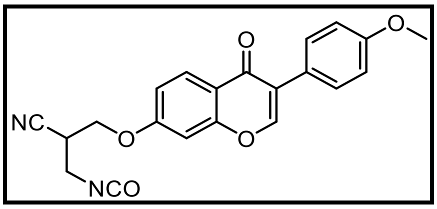

3-Isocyanato-4-((3-(4-methoxyphenyl)-4-oxo-4*H*-chromen-7-yl)oxy)butanenitrile (4l) Prepared according to the general procedure using 2-phenyloxirane (64.8 mg, 0.2 mmol) and BrCN (0.6 mmol, 1M in dioxane) at 50 °C. The product was purified by flash column chromatography using 100% EtOAc/hexanes to afford nitrile **4l** (45.9 mg, 61%) as white solid. **^{1}H NMR** (500 MHz, $CDCl_3$) δ 8.24-8.22 (m, 1H), 7.92 (s, 1H), 7.51-7.48 (m, 2H), 7.00-6.97 (m, 3H), 6.86-6.85 (m, 1H), 5.15-5.10 (m, 1H), 4.29-4.23 (m, 2H), 4.16-4.06 (m, 2H), 3.84 (s, 3H). **^{13}C NMR** (125 MHz, $CDCl_3$) δ 175.71, 161.97, 159.67, 157.67, 152.20, 141.50, 130.12, 128.28, 125.04, 124.00, 119.26, 114.30, 114.01, 108.62, 101.26, 78.68, 68.23, 57.01, 55.37. **HRMS (ESI)** m/z: calcd for $[M+H^+]$ $C_{21}H_{17}N_2O_5$ 377.1232, found: 377.1233.

Radical scavenger experiments

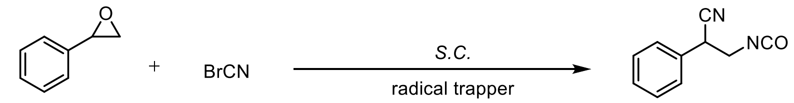

radical trapper	yield of product
TEMPO	59%
BHT	57%
Benzyl acrylate	62%

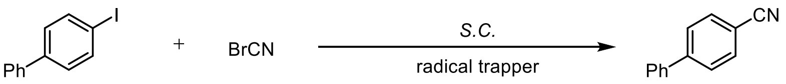

radical trapper	yield of product
TEMPO	68%
BHT	72%
Benzyl acrylate	79%

Reference

1. Cohen, D.T.; Buchwald, S.L. Mild Palladium-Catalyzed Cyanation of (Hetero)aryl Halides and Triflates in Aqueous Media. *Org. Lett.* **2015**, *17*, 202–205.

2. Ushkov, A.V.; Grushin, V.V. Rational Catalysis Design on the Basis of Mechanistic Understanding: Highly Efficient Pd-Catalyzed Cyanation of Aryl Bromides with NaCN in Recyclable Solvents. *J. Am. Chem. Soc.* **2011**, *133*, 10999–11005.

3. Mills, L.R.; Graham, J.M.; Patel, P.; Rousseaux, S.A.L. Ni-Catalyzed Reductive Cyanation of Aryl Halides and Phenol Derivatives via Transnitrilation. *J. Am. Chem. Soc.* **2019**, *141*, 19257–19262

4. Peng, S.; Yang, L. Copper-Catalyzed Cyanation of Aryl Iodides with Formamide as the Cyano Source. *Asian J. Org. Chem.* **2022**, *11*, e202200437.

5. Liu, S.-Z.; Li, J.; Xue, C.-G.; Xu, X.-T.; Lei, L.-S.; Huo, C.-Y.; Wang, Z.; Wang, S.-H. Copper-Promoted Cyanation of Aryl Iodides with *N,N*-dimethyl Aminomalononitrile. *Tetrahedron Lett.* **2021**, *65*, 152749.

6. Arakawa, Y.; Kang, S.M.; Tsuji, H.; Watanabea, J.; Konishi, G.-I. The Design of Liquid Crystalline Bistolane-Based Materials with Extremely High Birefringence. *RSC Adv.* **2016**, *6*, 92845–92851.

7. Vayer, M.; Zhang, S.; Moran, J.; Lebœuf D. Rapid and Mild Metal-Free Reduction of Epoxides to Primary Alcohols Mediated by HFIP. *ACS Catal.* **2022**, *12*, 3309–3316.

8. Park, J.-H.; Kim, Y.-K.; Lee, E.-Y.; Jeong, E.-J.; Hwang, S.-H. Triazine-Based Compounds and Organic Light-Emitting Devices Including Triazine-Based Compounds. United States, US20150171336.

9. Zhang, L.; Lu, P.; Wang, Y. $Cu(NO_3)_2\cdot 3H_2O$-Mediated Cyanation of Aryl Iodides and Bromides Using DMF as a Aingle Surrogate of Cyanide. *Chem. Commun.* **2015**, *51*, 2840–2843.

10. Mohammadinezhada, A.; Akhlaghinia, B. Co^{II} Immobilized on an Aminated Magnetic Metal–Organic Framework Catalyzed C–N and C–S Bond Forming Reactions: A Journey for the Mild and Efficient Synthesis of Arylamines and Arylsulfides. *New J. Chem.* **2019**, *43*, 15525–15538.

11 Li C.; Mizuno N.; Murata K.; Ishii K.; Suenobu T.; Yamaguchi K.; Suzuki K. Selectivity Switch in the Aerobic Oxygenation of Sulfides Photocatalysed by Visible-light-responsive Decavanadate. *Green Chem.,* **2020***, 22,* 3896–3905.

12 Whiting E.; Lanning M. E.; Scheenstra J. A.; Fletcher S. Chromatography-Free Entry to Substituted Salicylonitriles: Mitsunobu-Triggered Domino Reactions of Salicylaldoximes. *J. Org. Chem.* **2015**, *80*, 1229–1234.

13 Srivastava R.R.; Zych A.J.; Jenkins D.M.; Wang H.-J.; Chen Z.-J.; Fairfax D. J. Application of Polymer-Supported Triphenylphosphine and Microwave Irradiation to the Palladium-Catalyzed Cyanation of Aryl Triflates. *Synth. Commun.* **2007**, *37*, 431–438.

14 Maestri G.; Cañeque T.; Della Ca' N.; Derat E.; Catellani M.; Chiusoli G. P.; Malacria M. Pd Catalysis in Cyanide-Free Synthesis of Nitriles from Haloarenes via Isoxazolines. *Org. Lett.* **2016***, 18,* 6108–6111.

15 Kim, S. H.; An, J. H.; Lee, J. H. Highly Chemoselective Deoxygenation of *N*-heterocyclic *N*-oxides under Transition Metal-free Conditions. *Org. Biomol. Chem*., **2021**, *19*, 3735–3742.

NMR spectrum

2a ([1]**H NMR**, 500 MHz, $CDCl_3$)

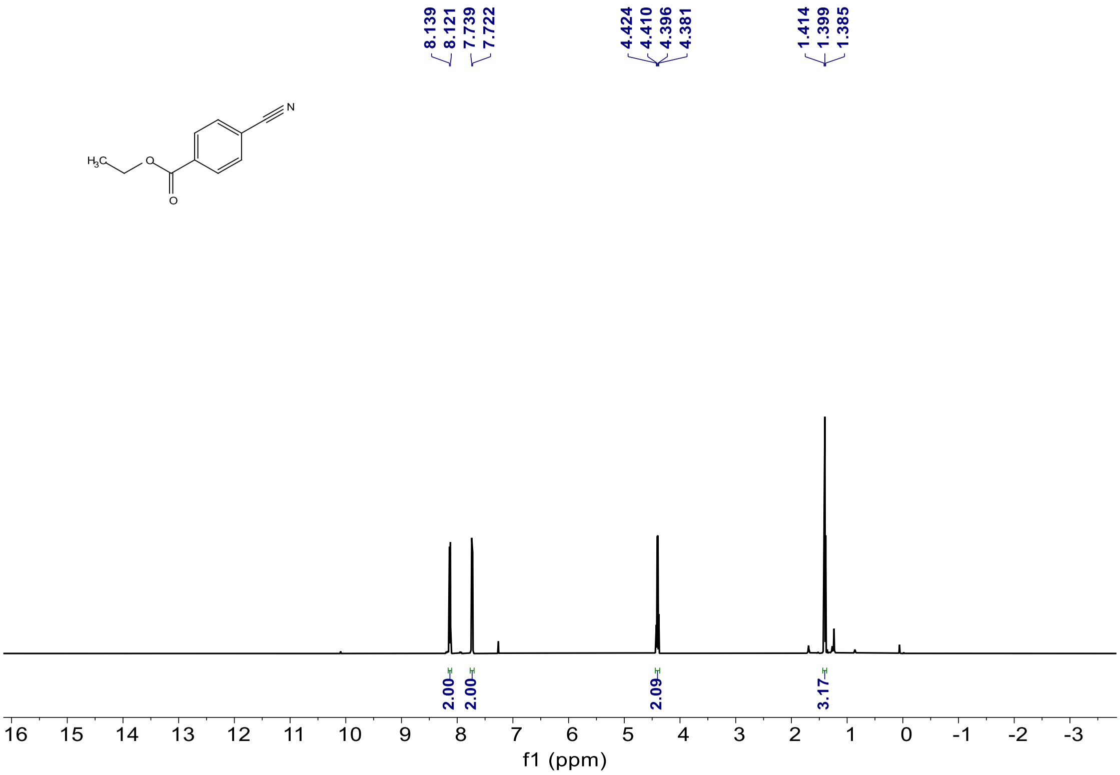

2a [13]**C NMR** (125 MHz, $CDCl_3$)

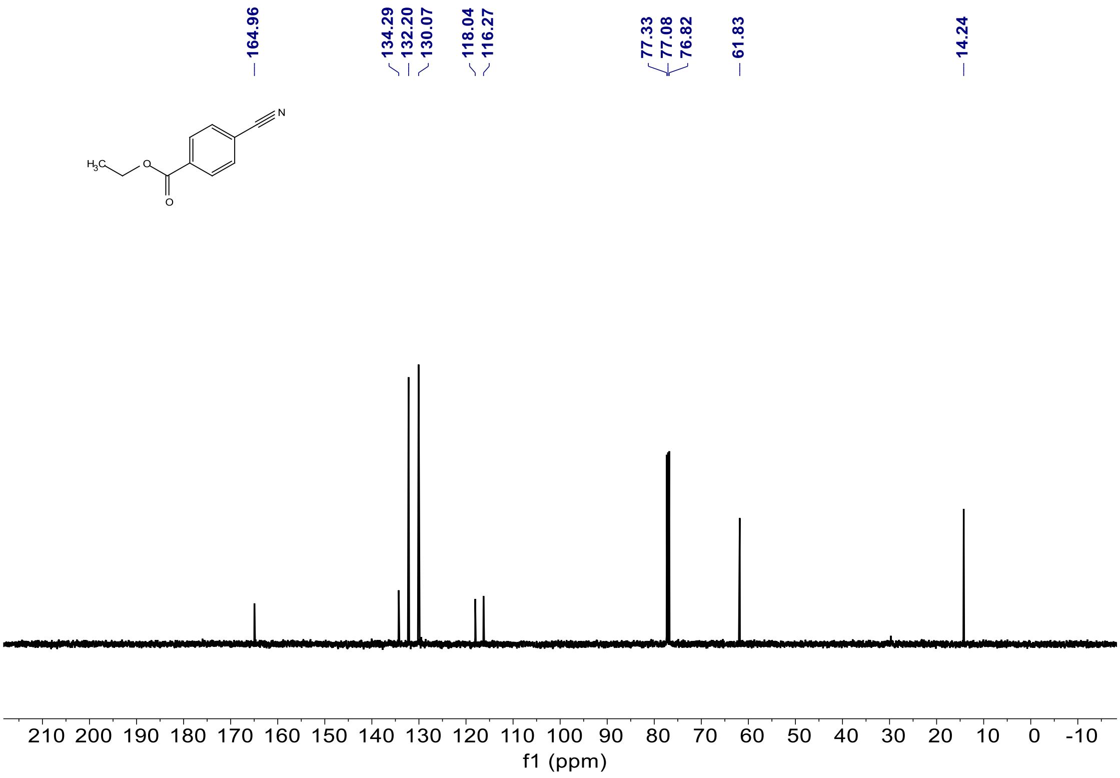

2b ([1]**H NMR**, 500 MHz, CDCl$_3$)

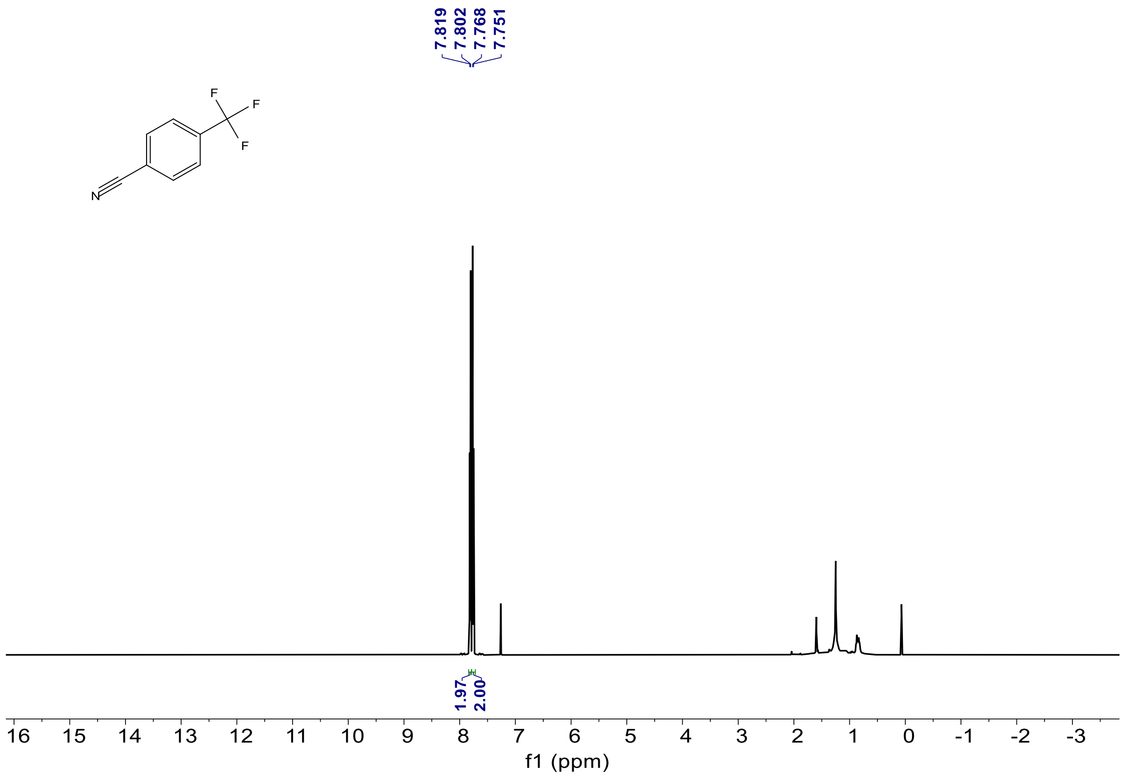

2b [13]**C NMR** (125 MHz, CDCl$_3$)

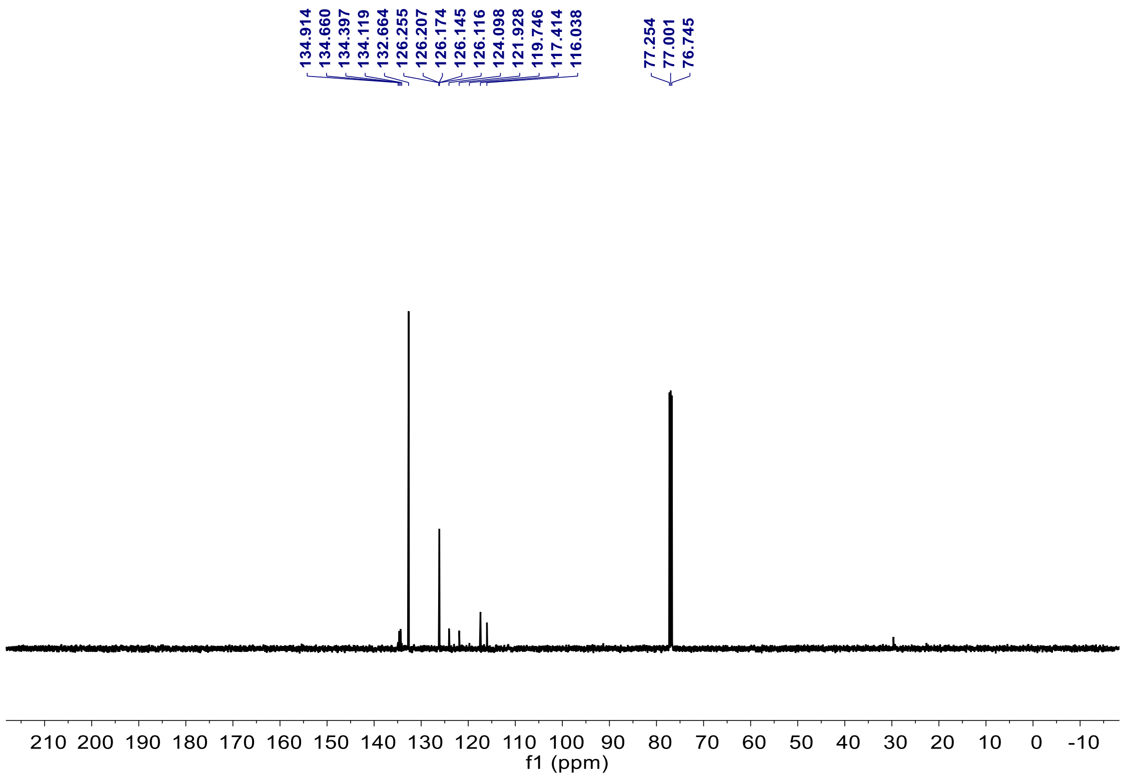

2b ^{19}F NMR (471 MHz, $CDCl_3$)

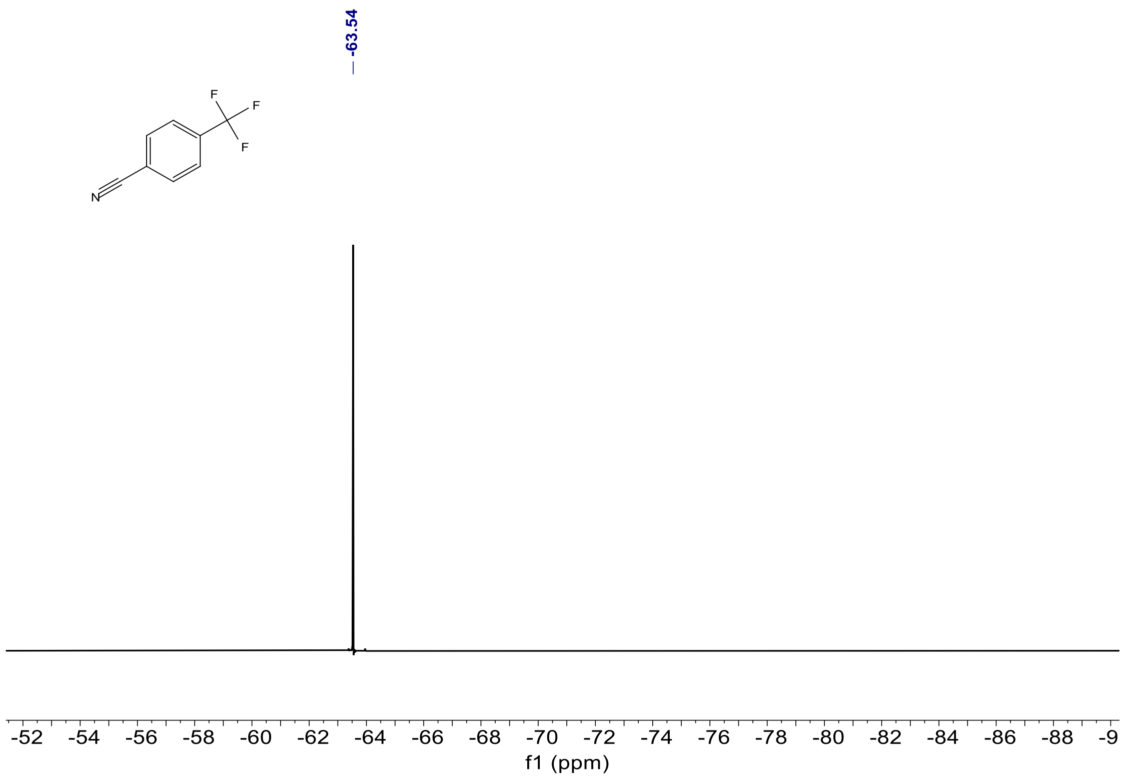

2c (**^{1}H NMR**, 500 MHz, $CDCl_3$)

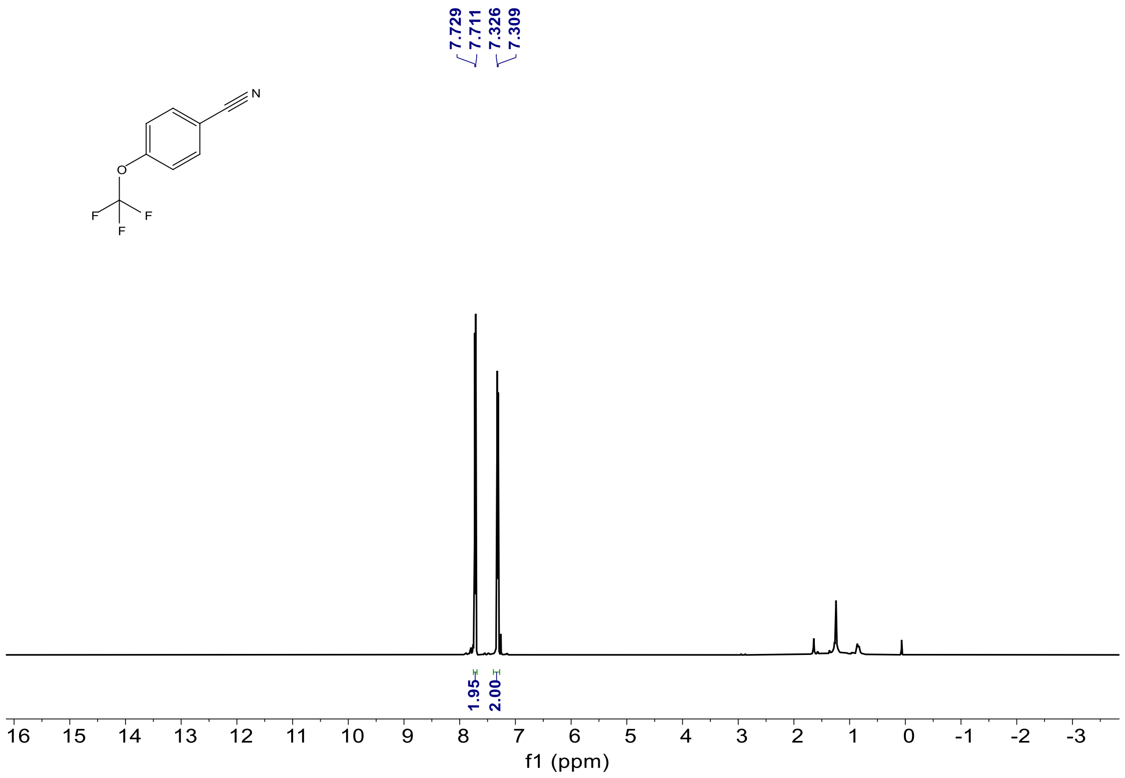

2c ^{13}C NMR (125 MHz, $CDCl_3$)

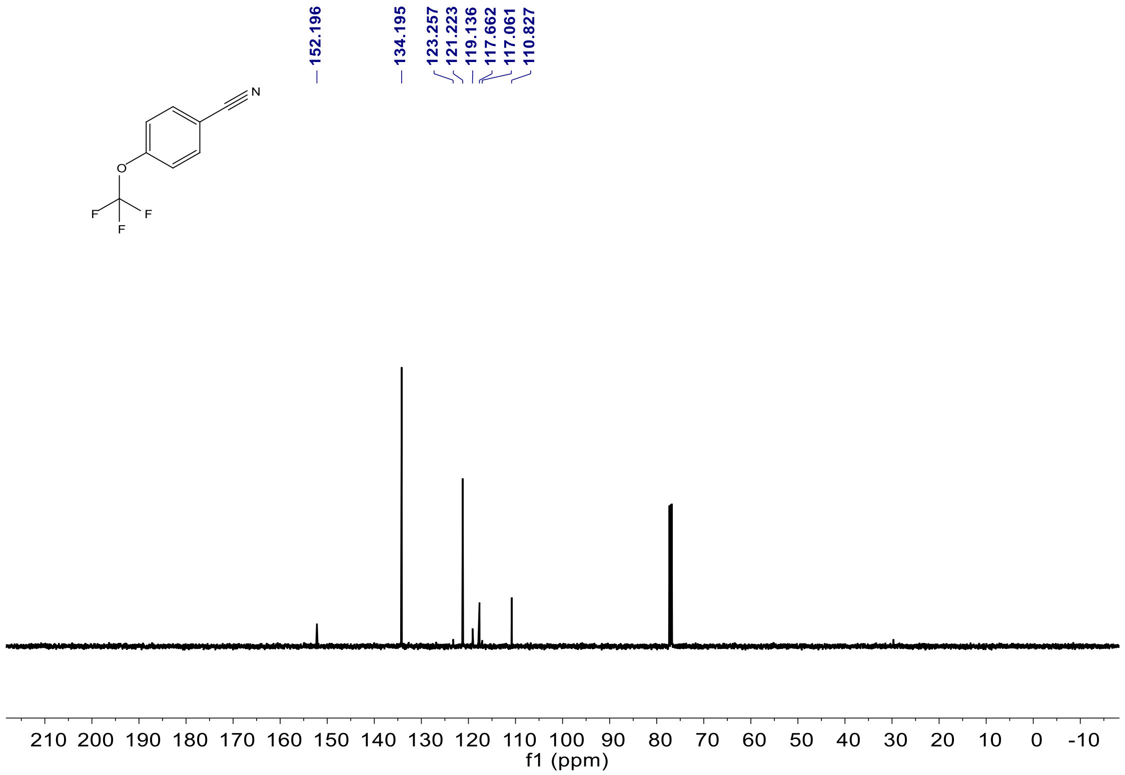

2c ^{19}F NMR (471 MHz, $CDCl_3$)

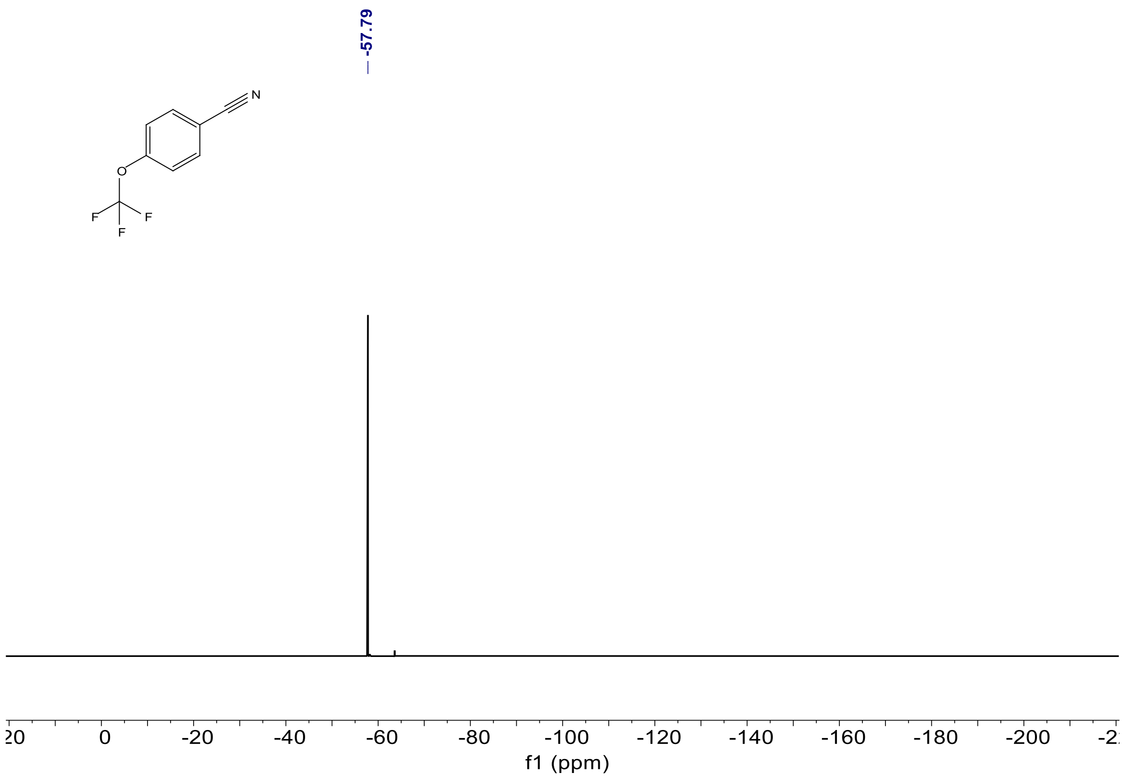

2d (^{1}**H NMR**, 500 MHz, CDCl$_3$)

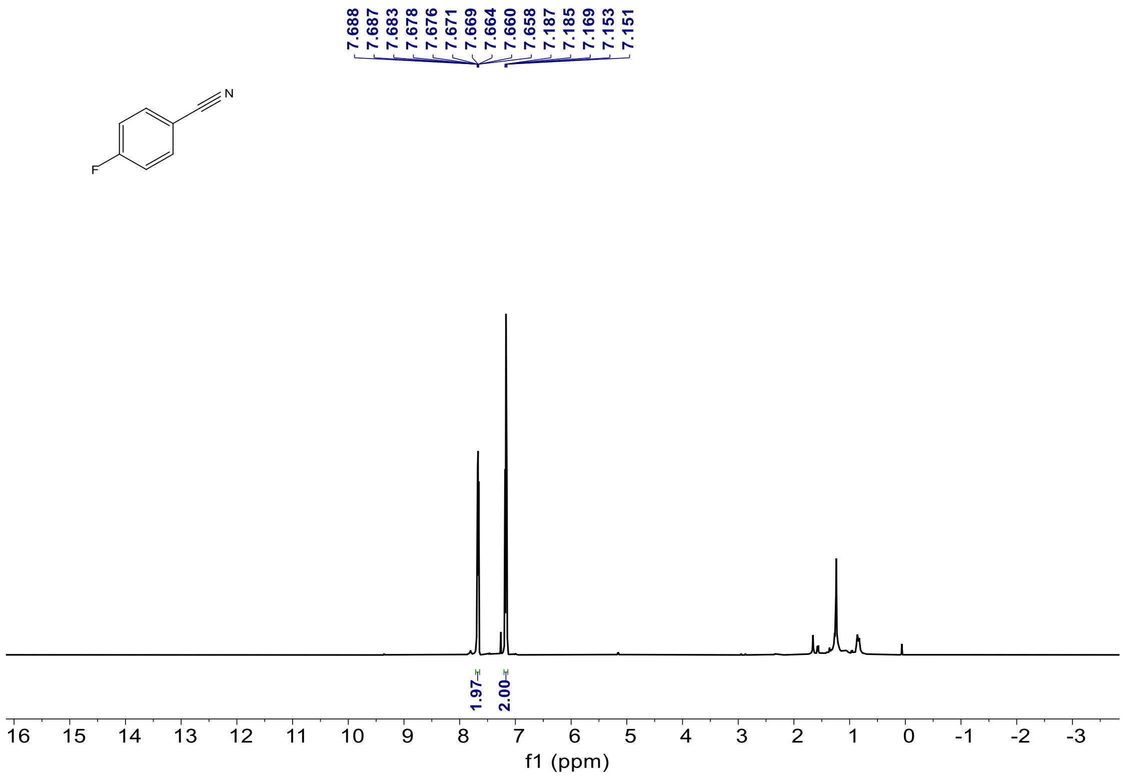

2d ^{13}**C NMR** (125 MHz, CDCl$_3$)

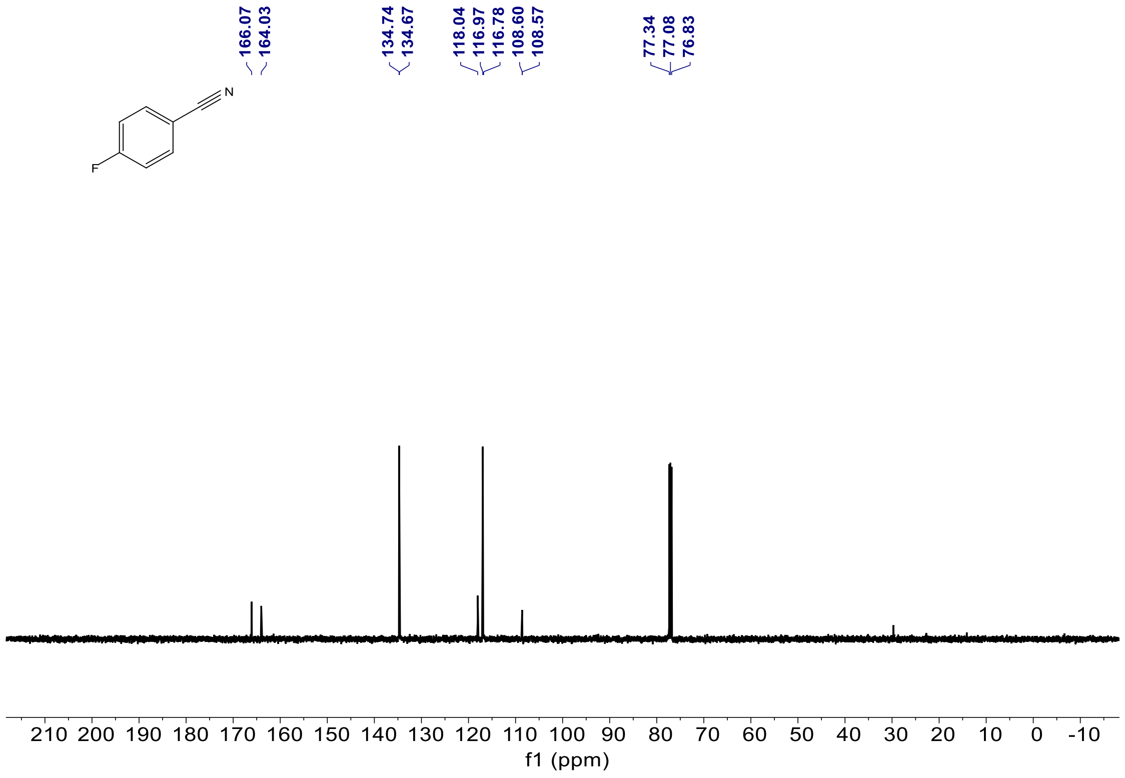

2e (**^{1}H NMR**, 500 MHz, $CDCl_3$)

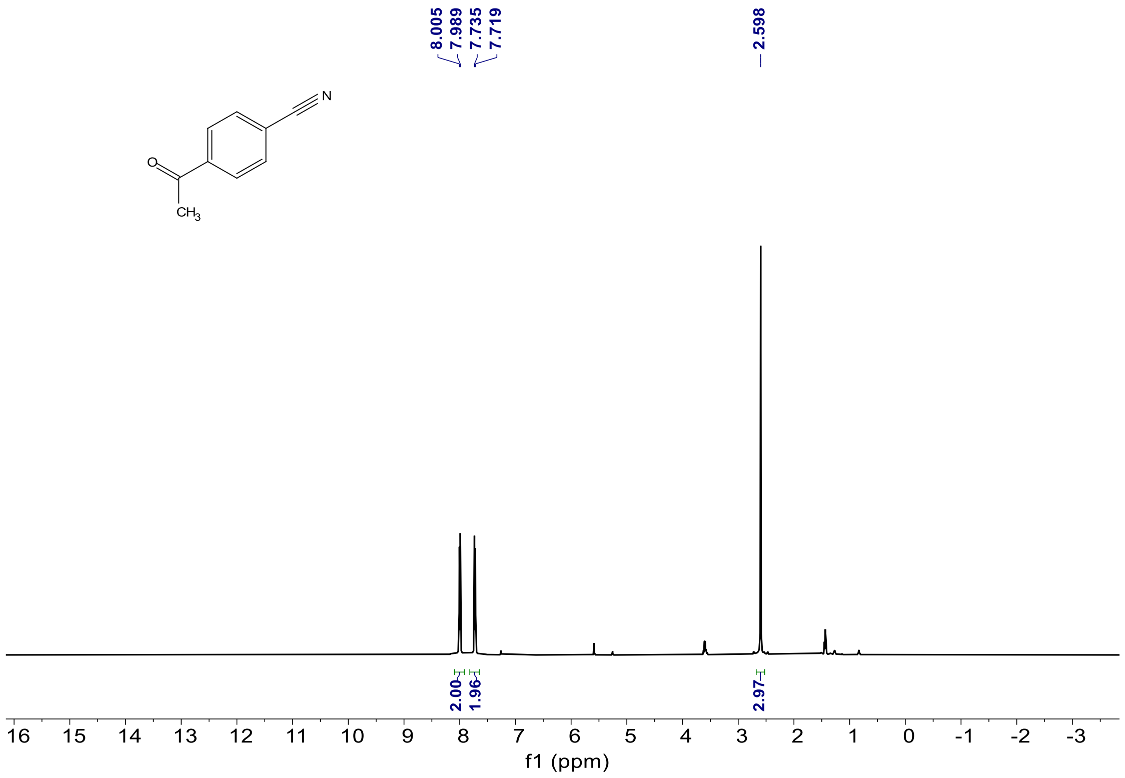

2e ^{13}C NMR (125 MHz, $CDCl_3$)

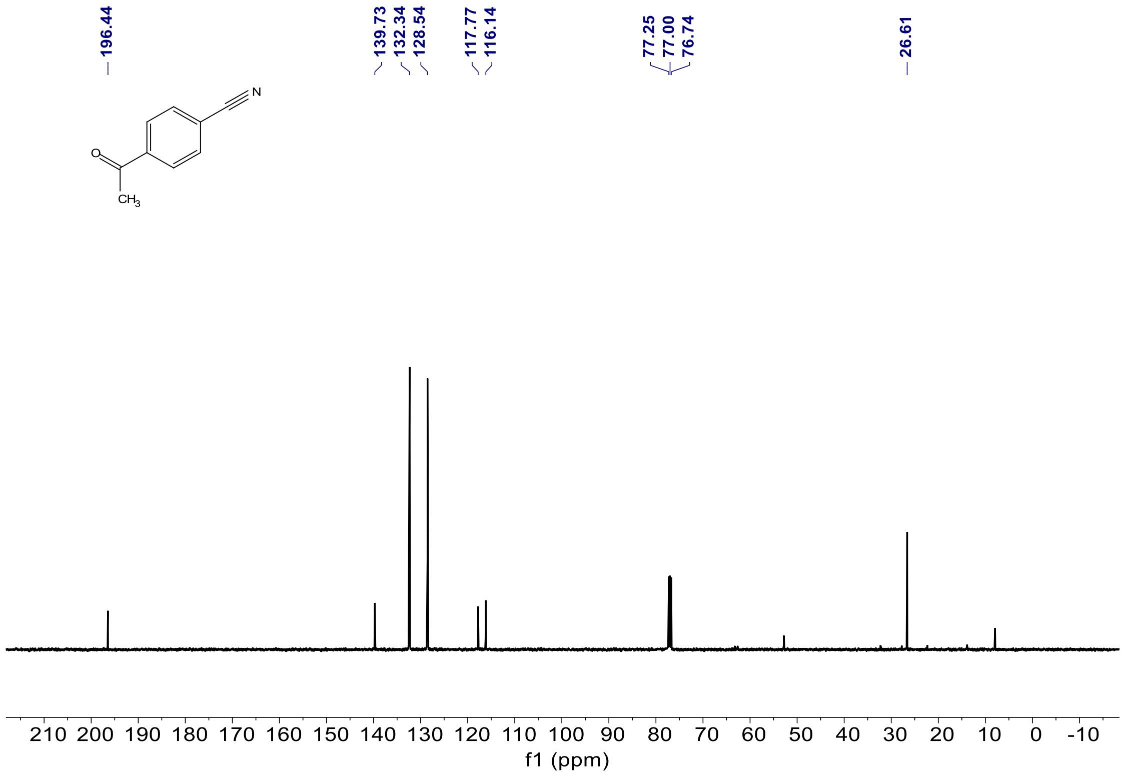

2f (**^{1}H NMR**, 400 MHz, $CDCl_3$)

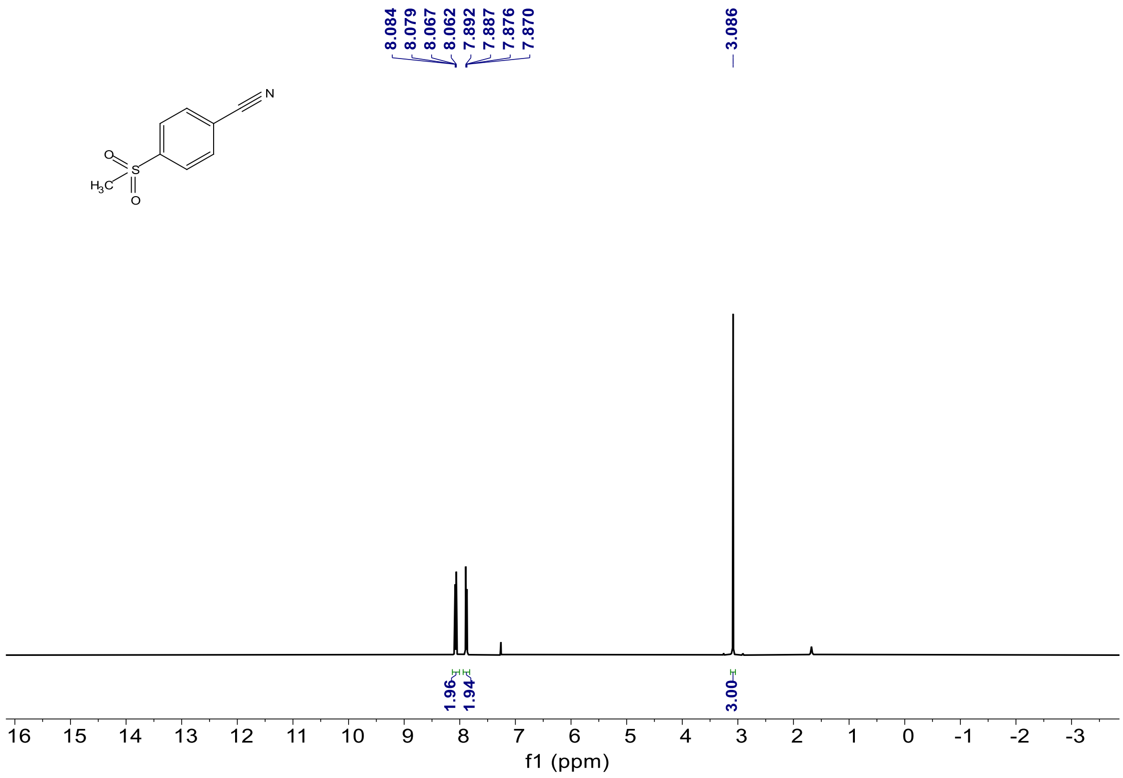

2f ^{13}**C NMR** (100 MHz, $CDCl_3$)

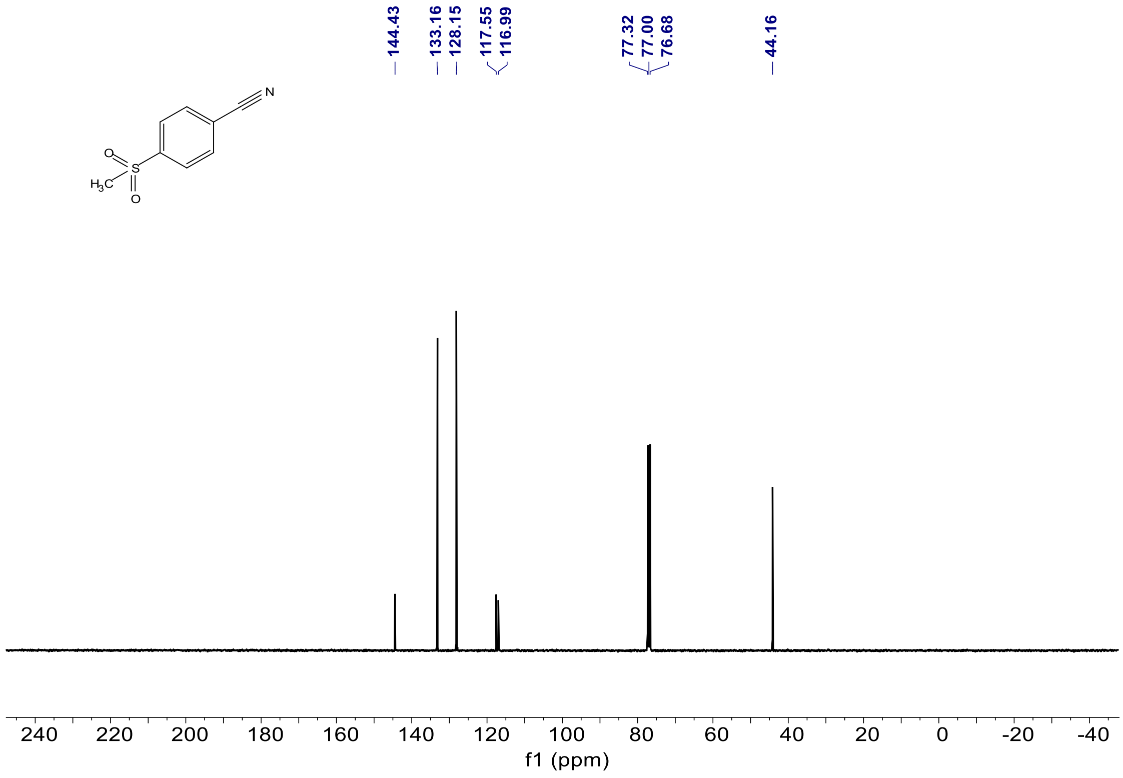

2g ([1]**H NMR**, 500 MHz, CDCl3)

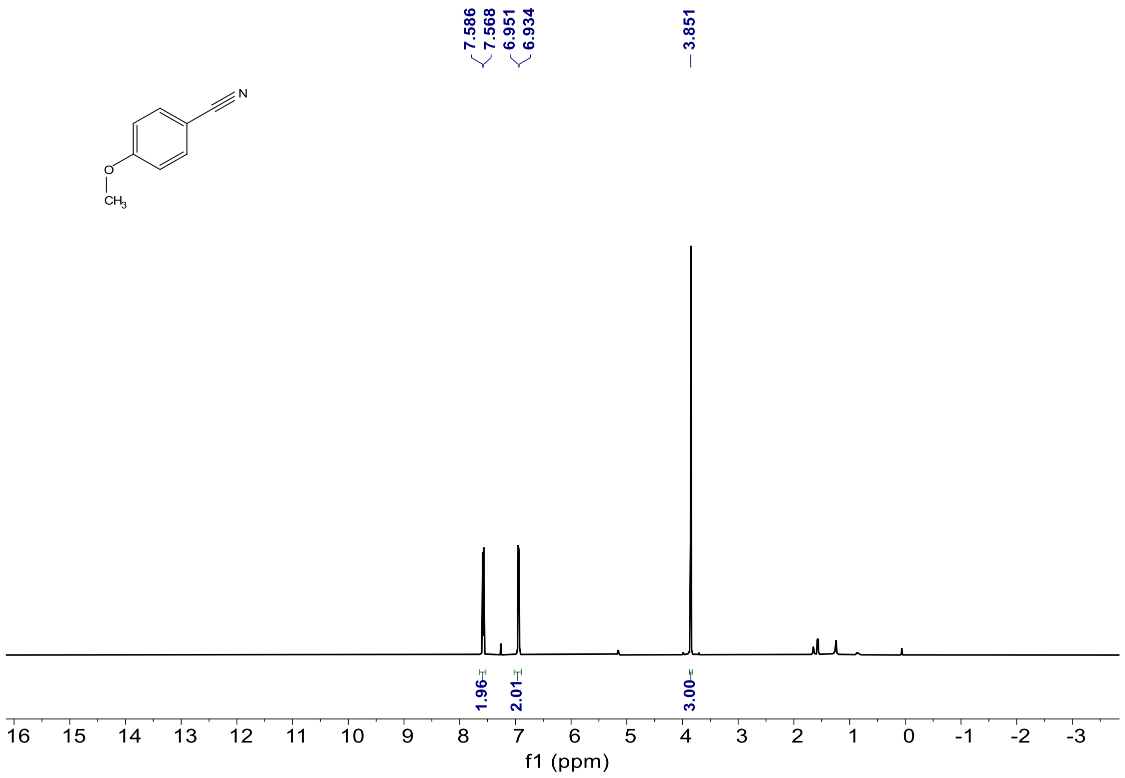

2g [13]**C NMR** (125 MHz, CDCl3)

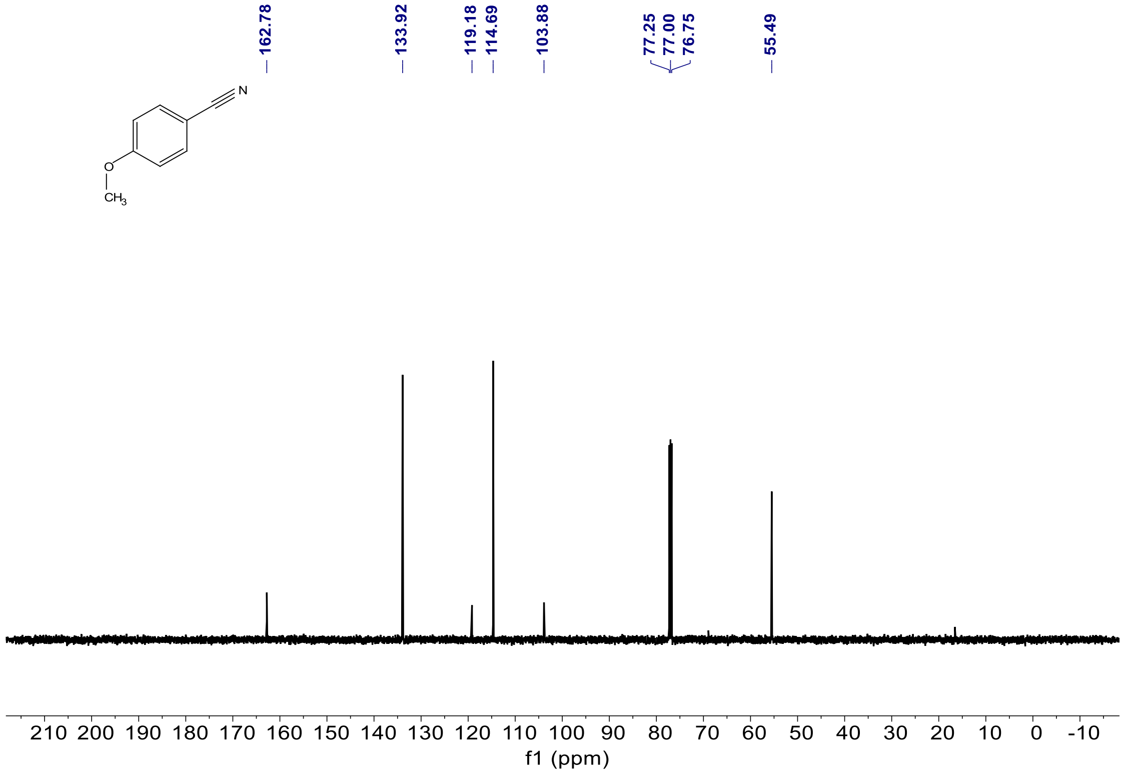

2h (**^{1}H NMR**, 500 MHz, $CDCl_3$)

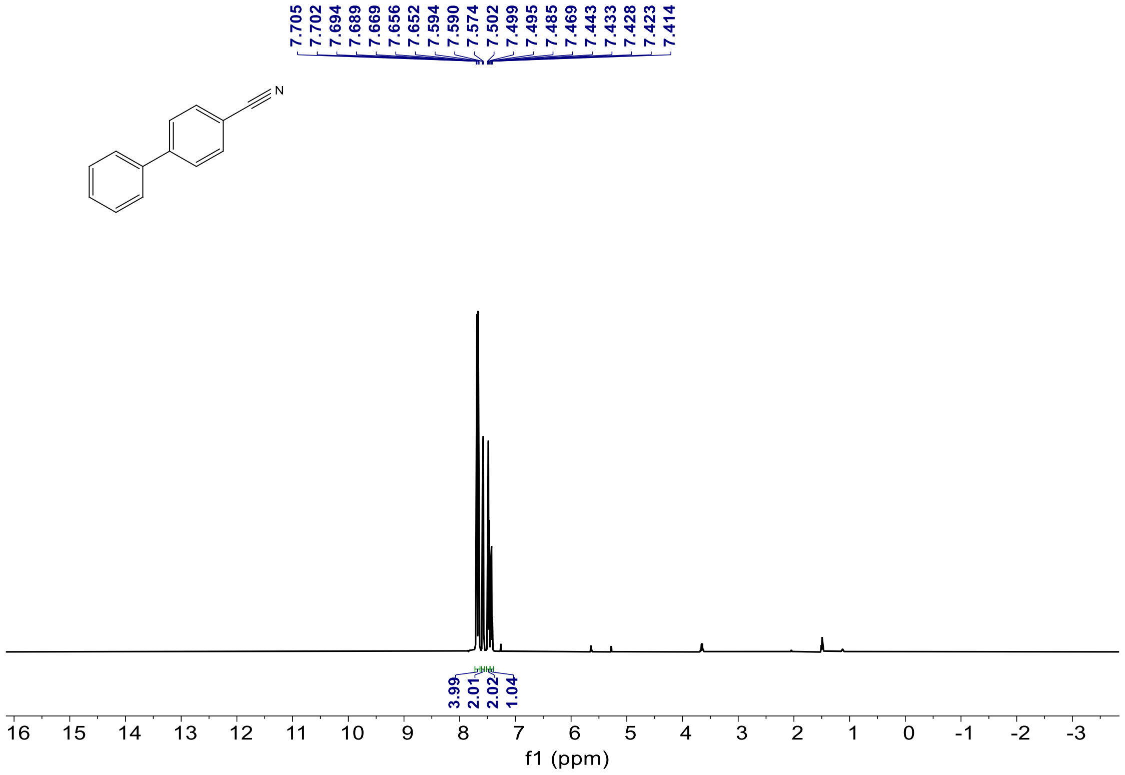

2h ^{13}C NMR (125 MHz, $CDCl_3$)

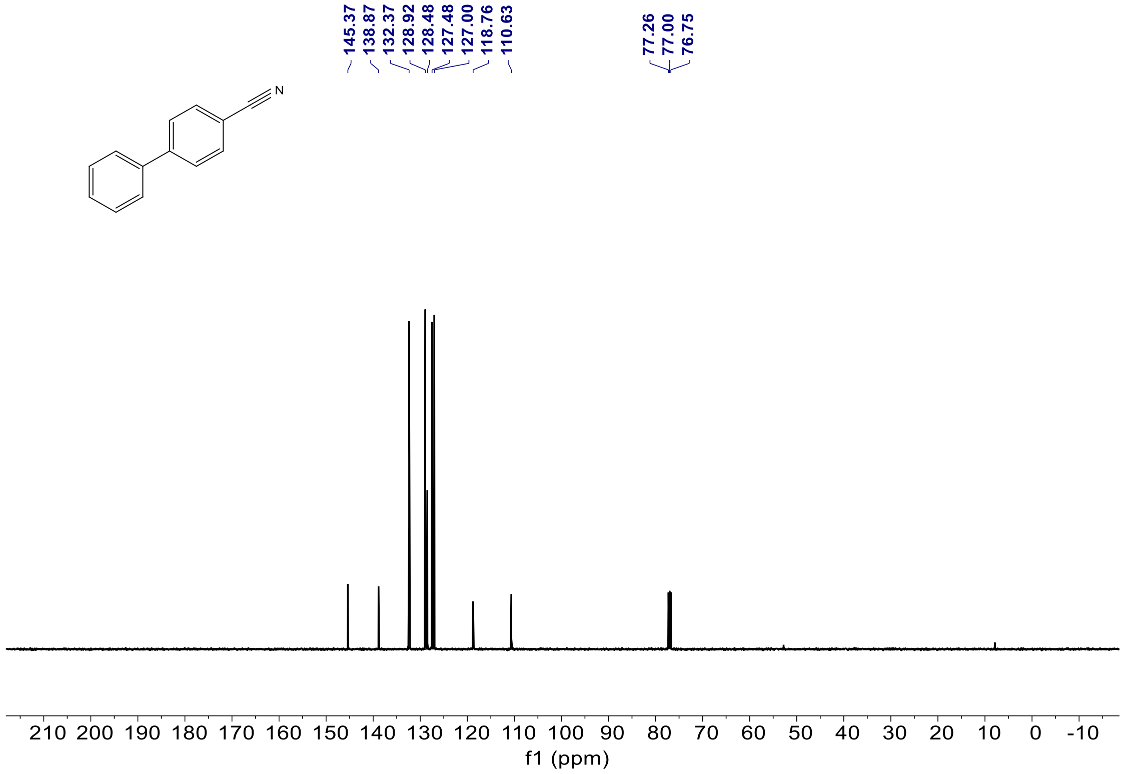

2i ([1]**H NMR**, 500 MHz, $CDCl_3$)

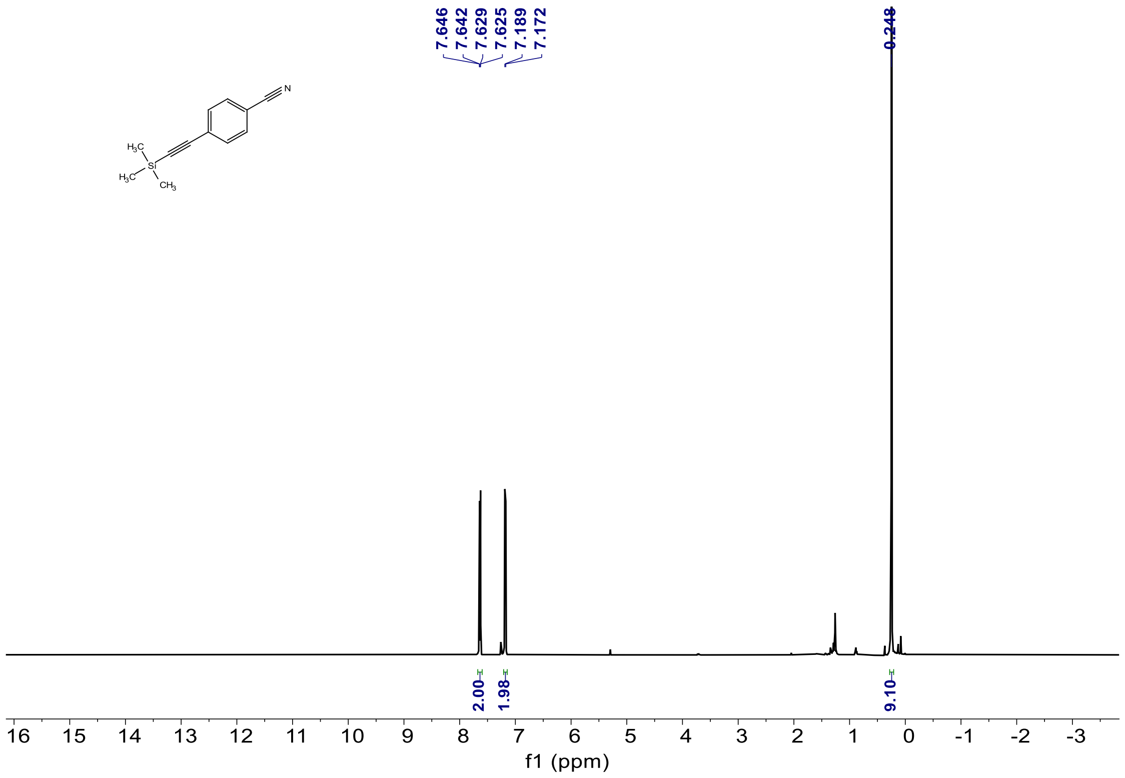

2i [13]**C NMR** (125 MHz, $CDCl_3$)

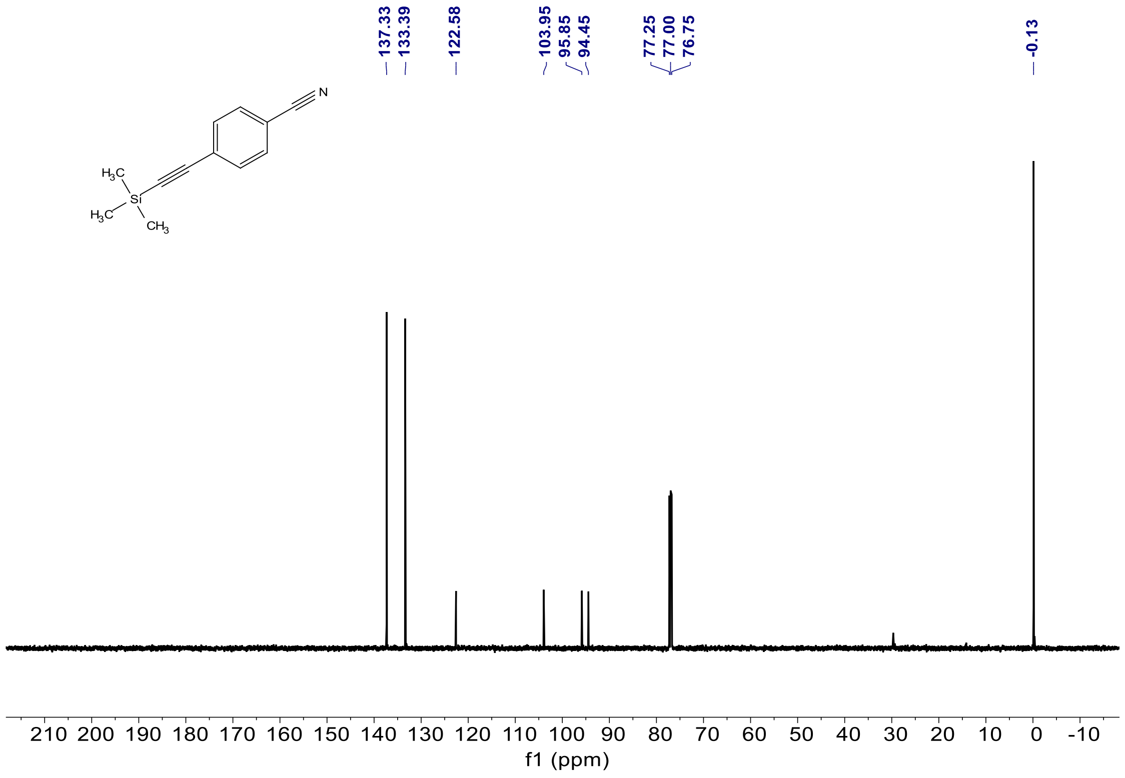

2j ([1]**H NMR**, 500 MHz, $CDCl_3$)

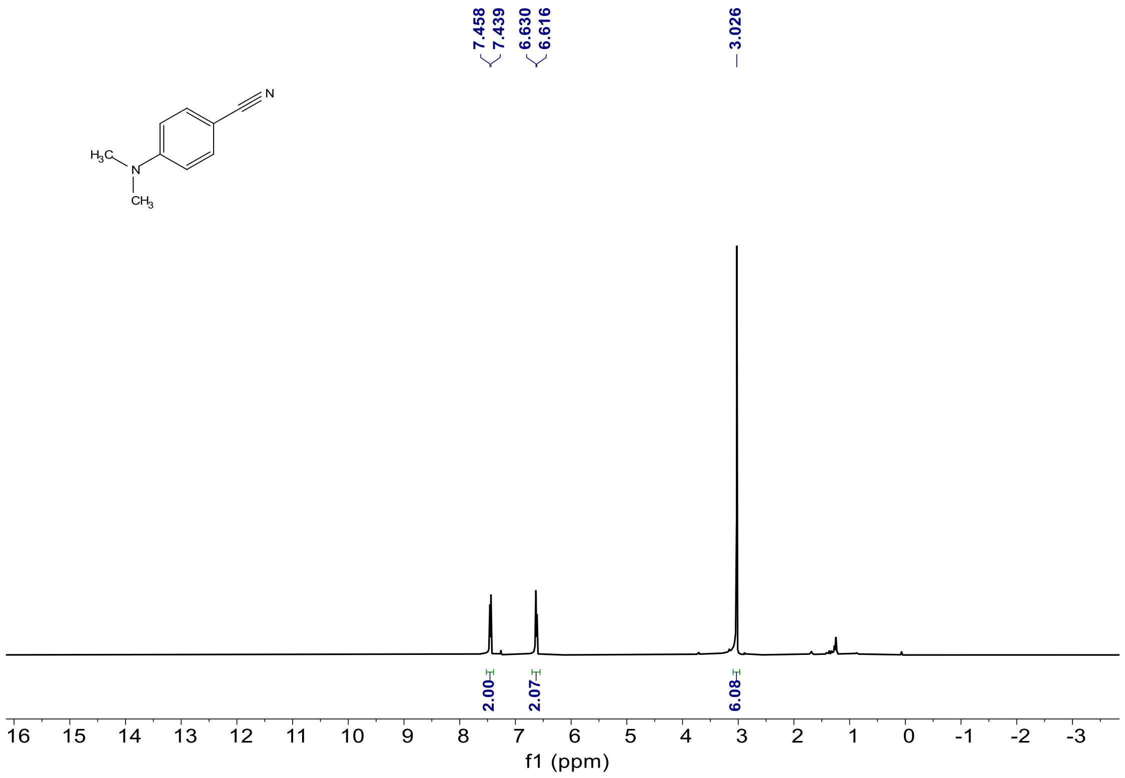

2j [13]**C NMR** (125 MHz, $CDCl_3$)

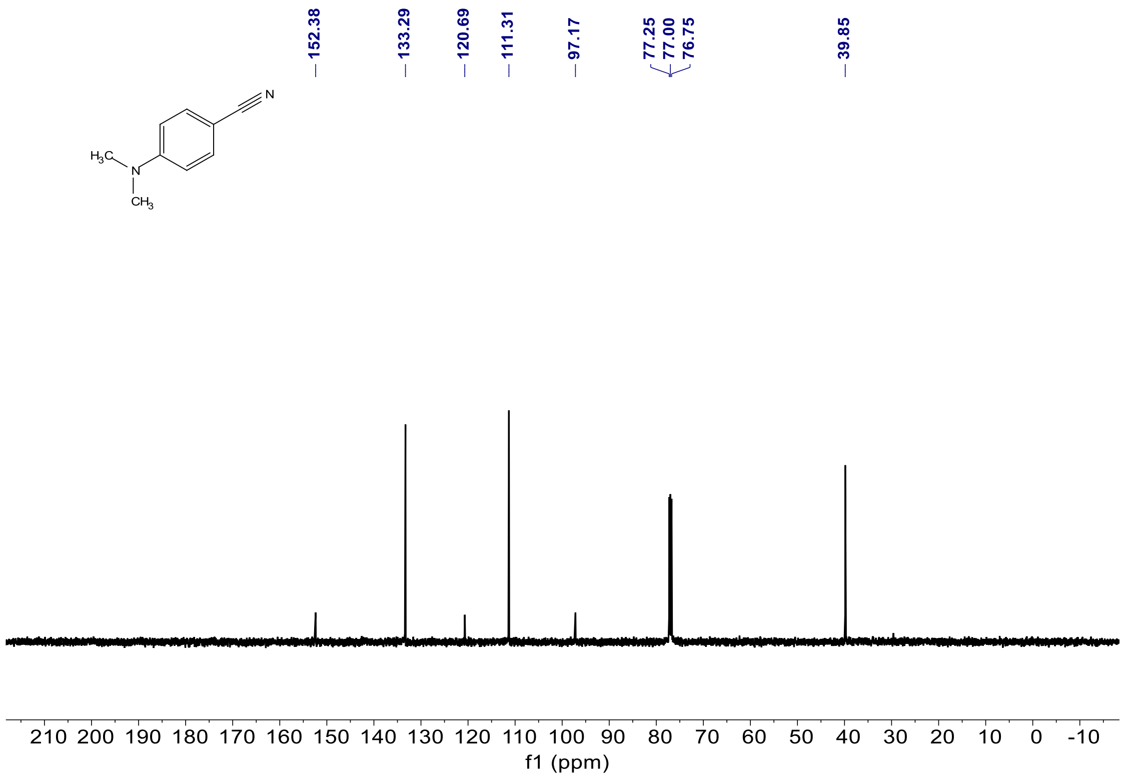

2k (^{1}**H NMR**, 500 MHz, $CDCl_3$)

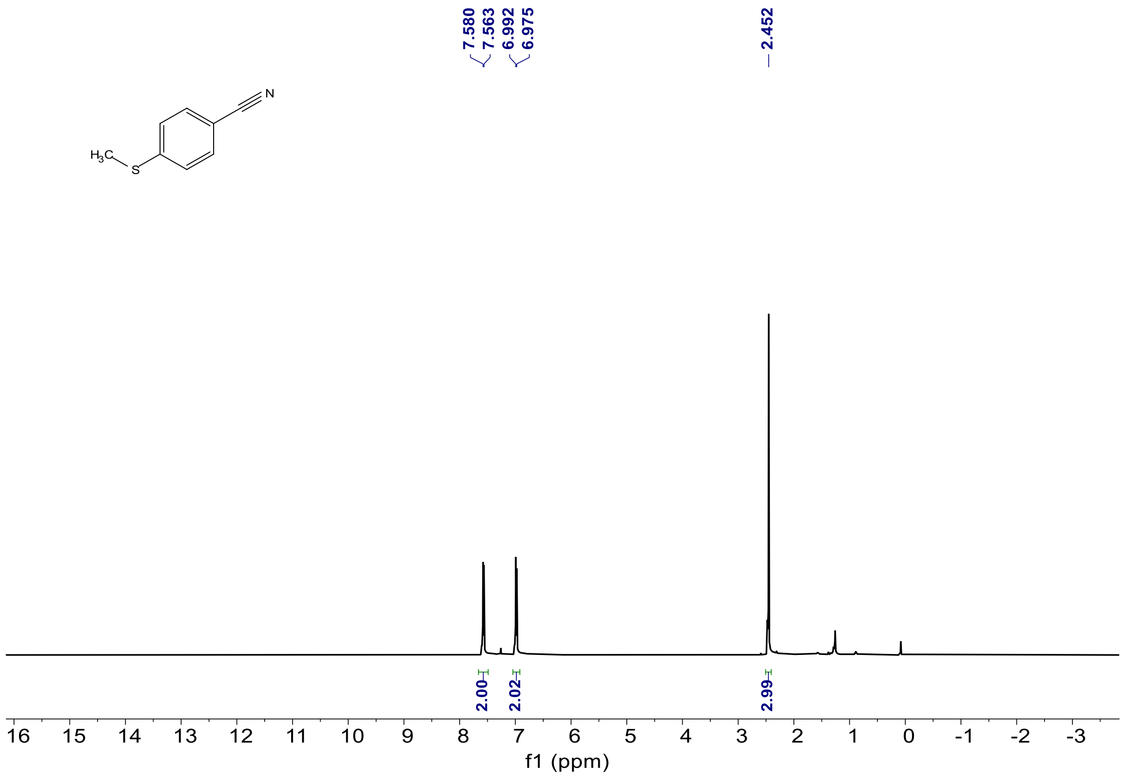

2k ^{13}**C NMR** (125 MHz, $CDCl_3$)

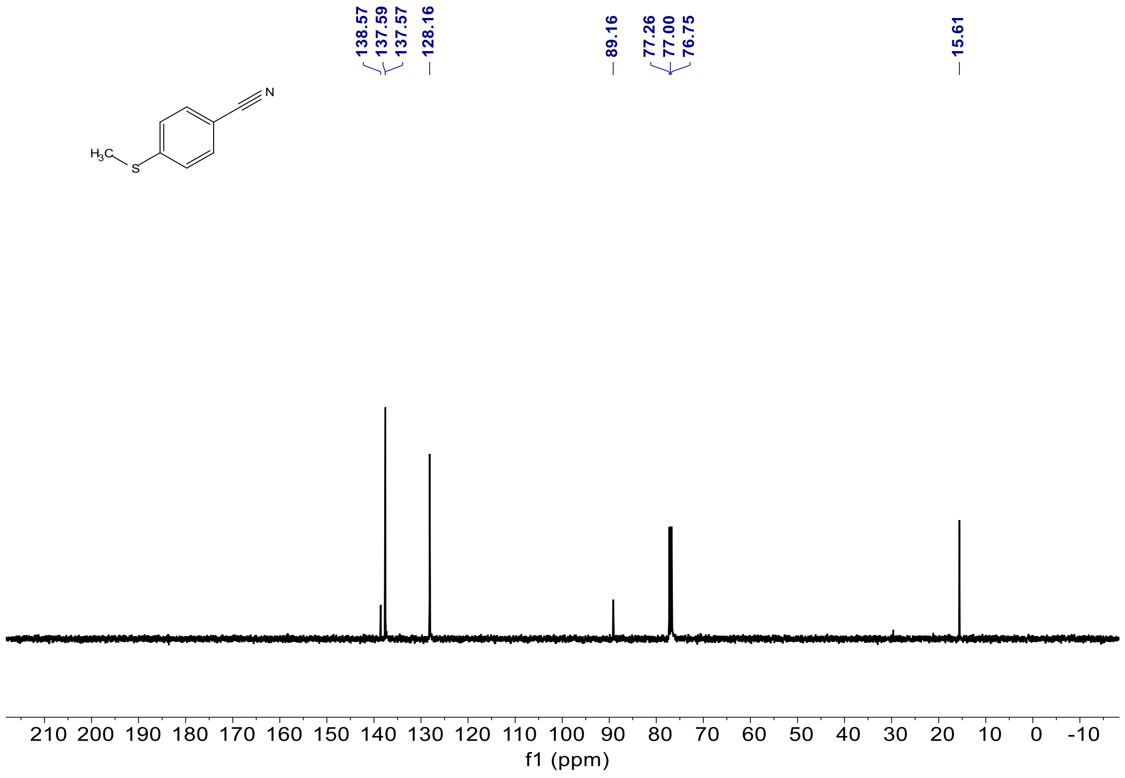

2l (**^{1}H NMR**, 500 MHz, $CDCl_3$)

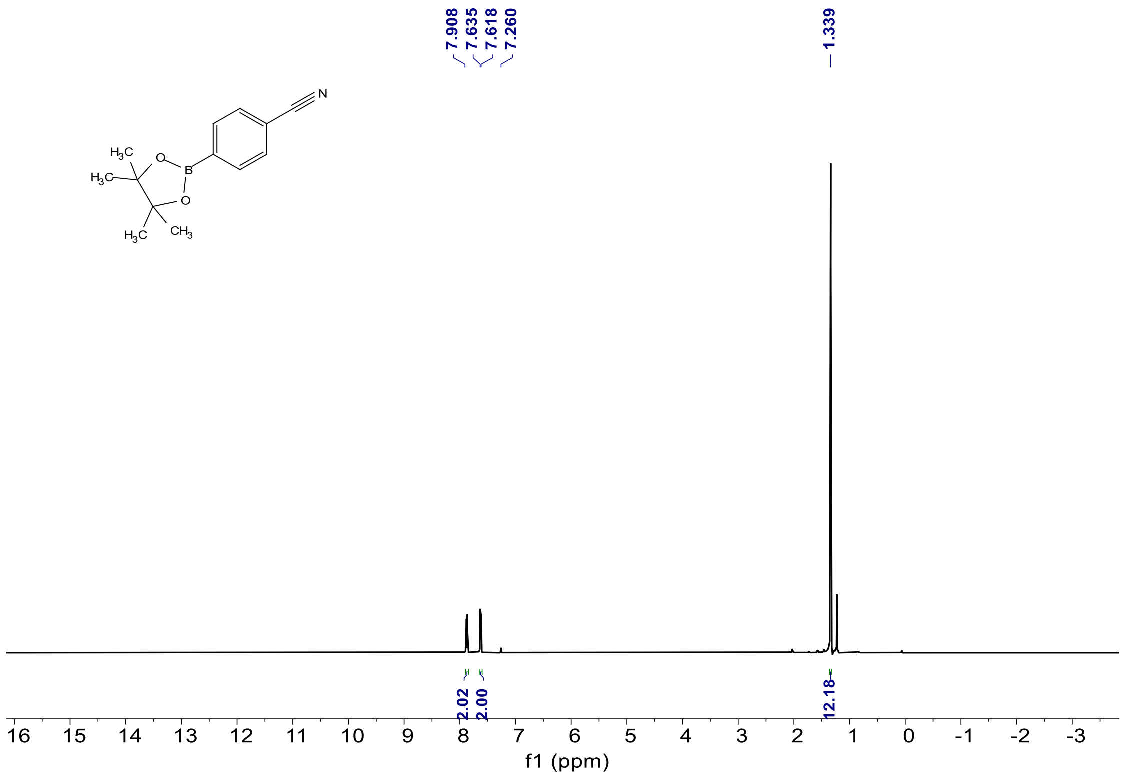

2l **^{13}C NMR** (125 MHz, $CDCl_3$)

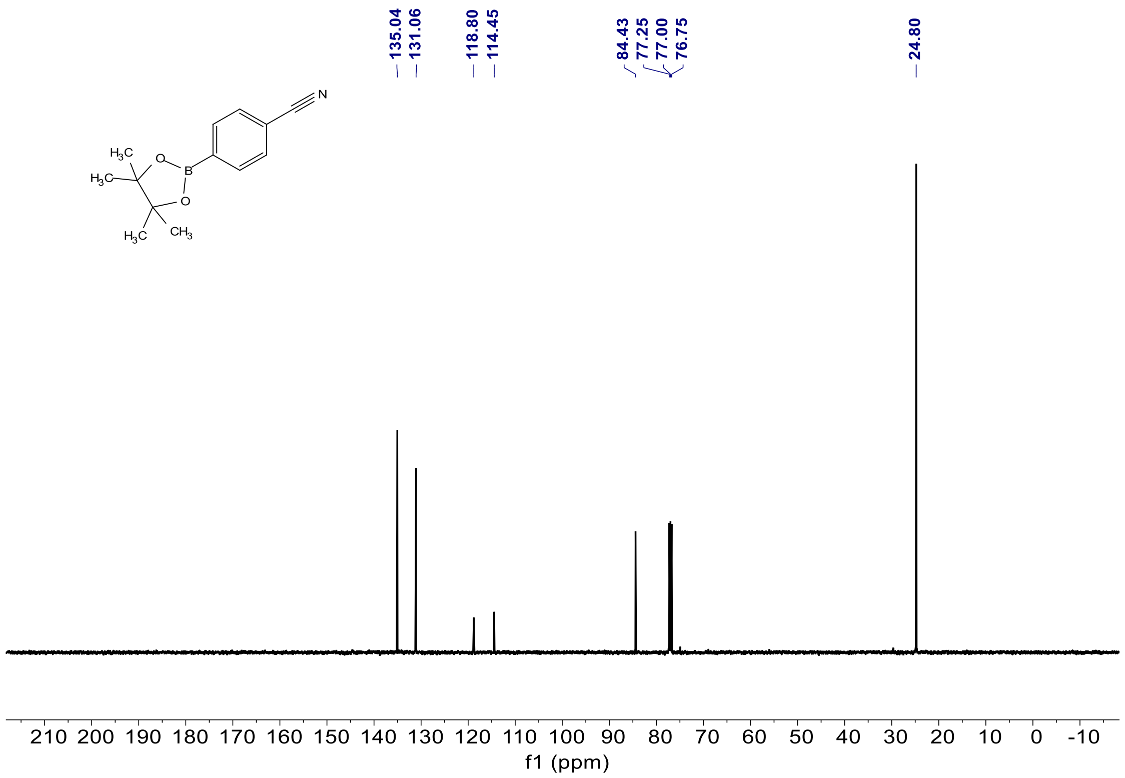

2m (^{1}**H NMR**, 500 MHz, $CDCl_3$)

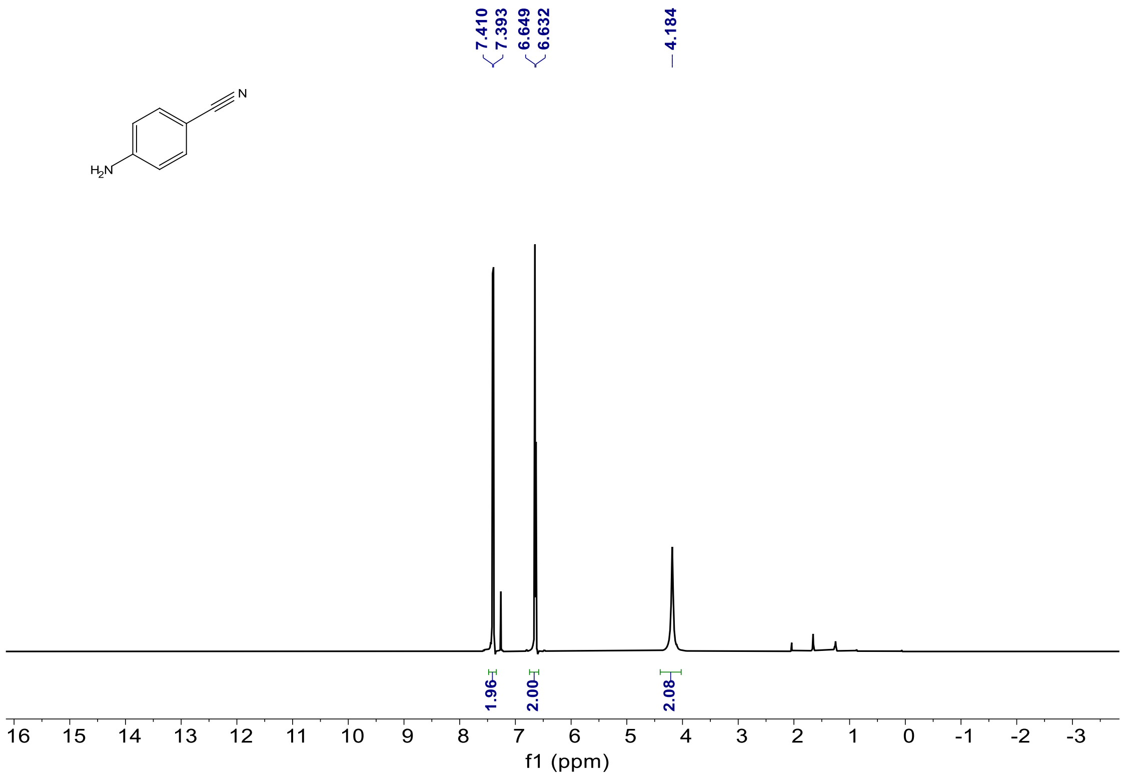

2m ^{13}**C NMR** (125 MHz, $CDCl_3$)

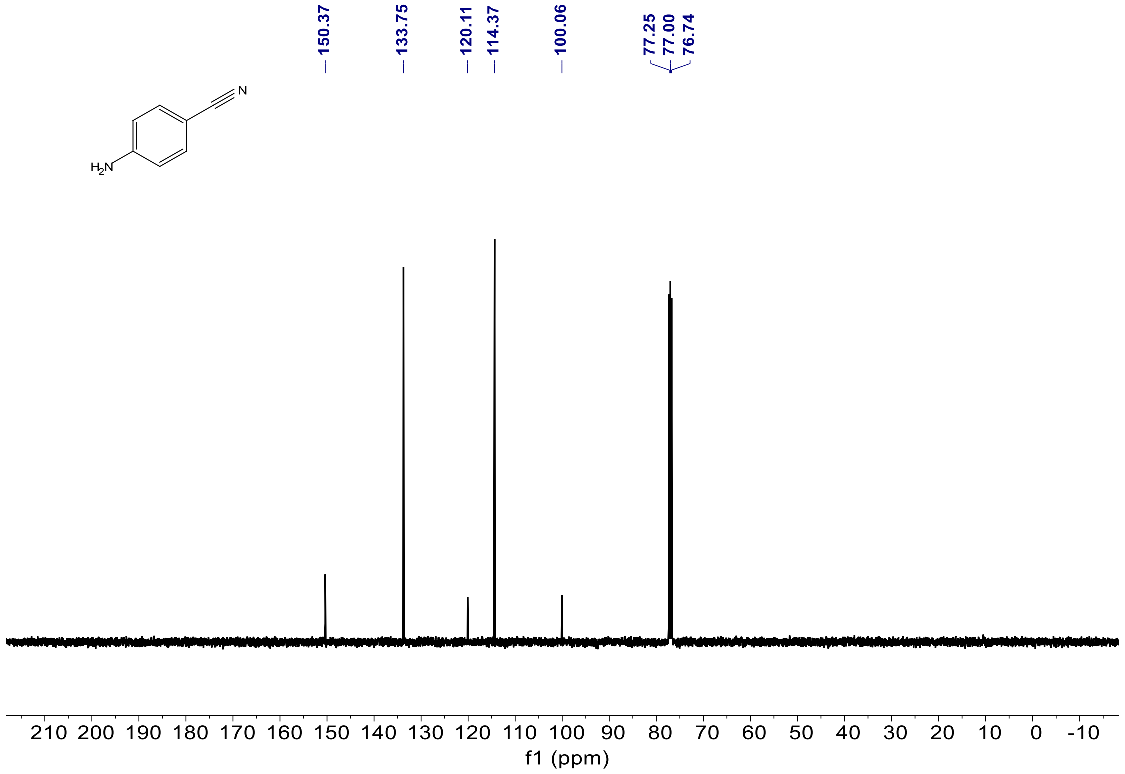

2n ([1]**H NMR**, 500 MHz, CDCl3)

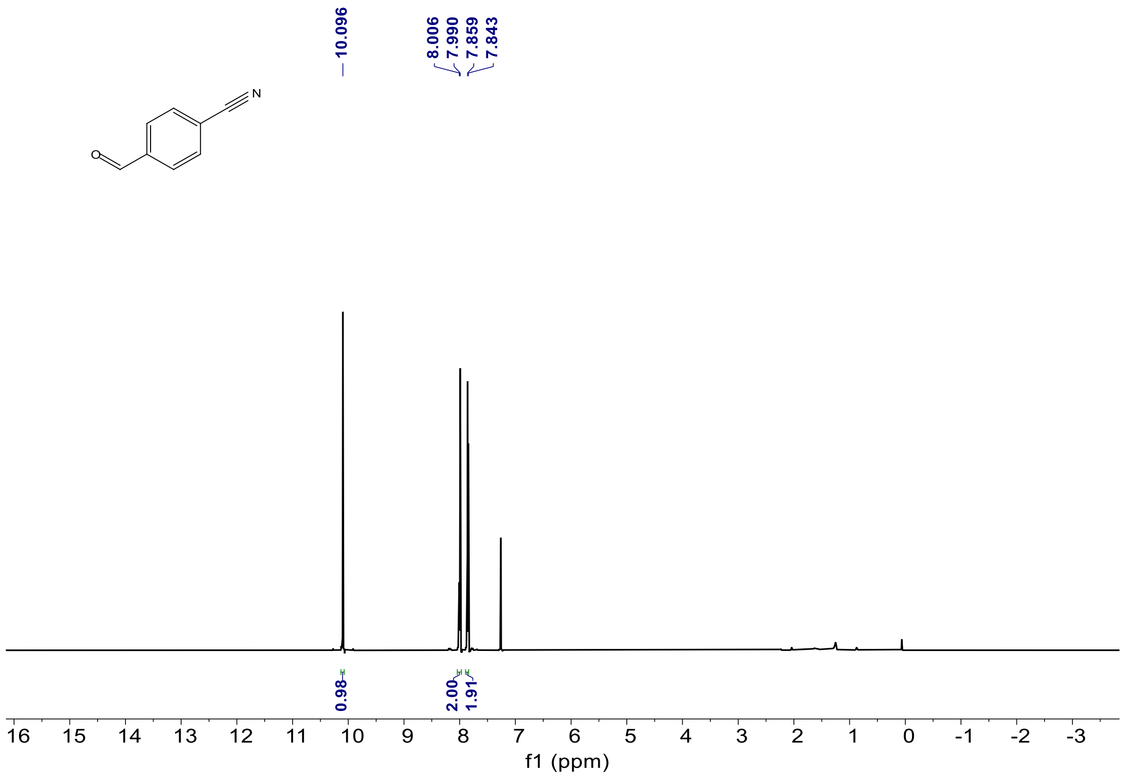

2n [13]**C NMR** (125 MHz, CDCl3)

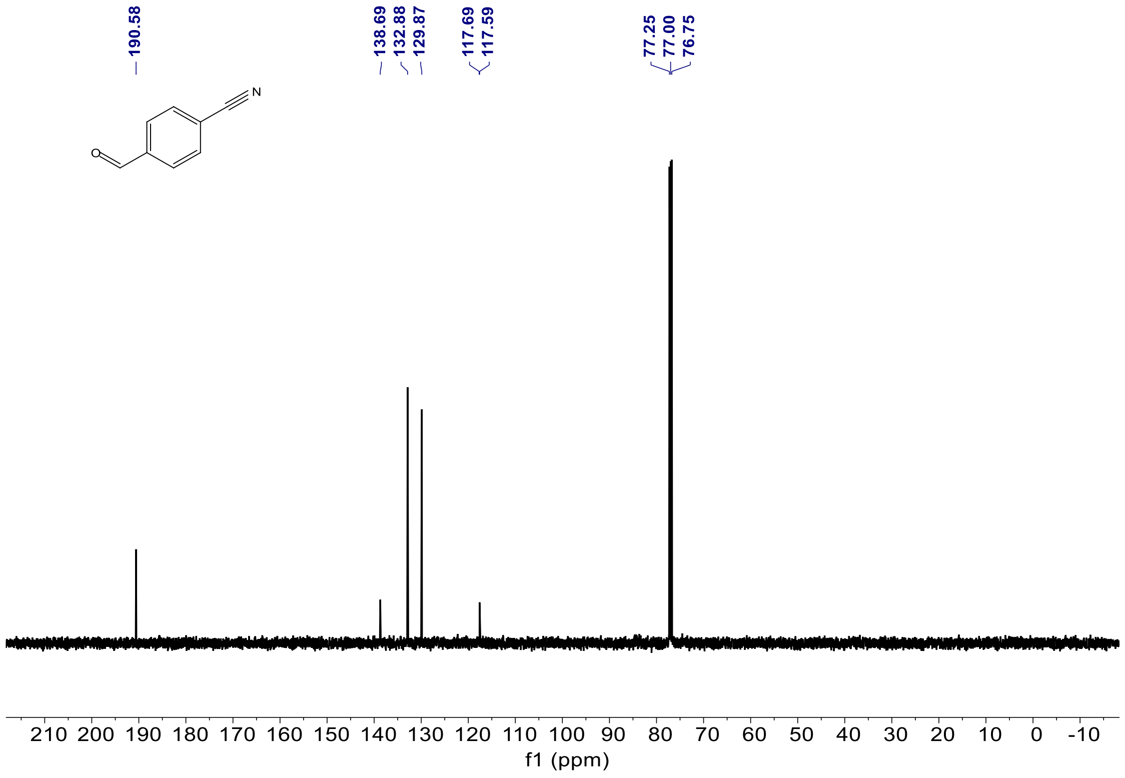

2o ([1]**H NMR**, 500 MHz, $CDCl_3$)

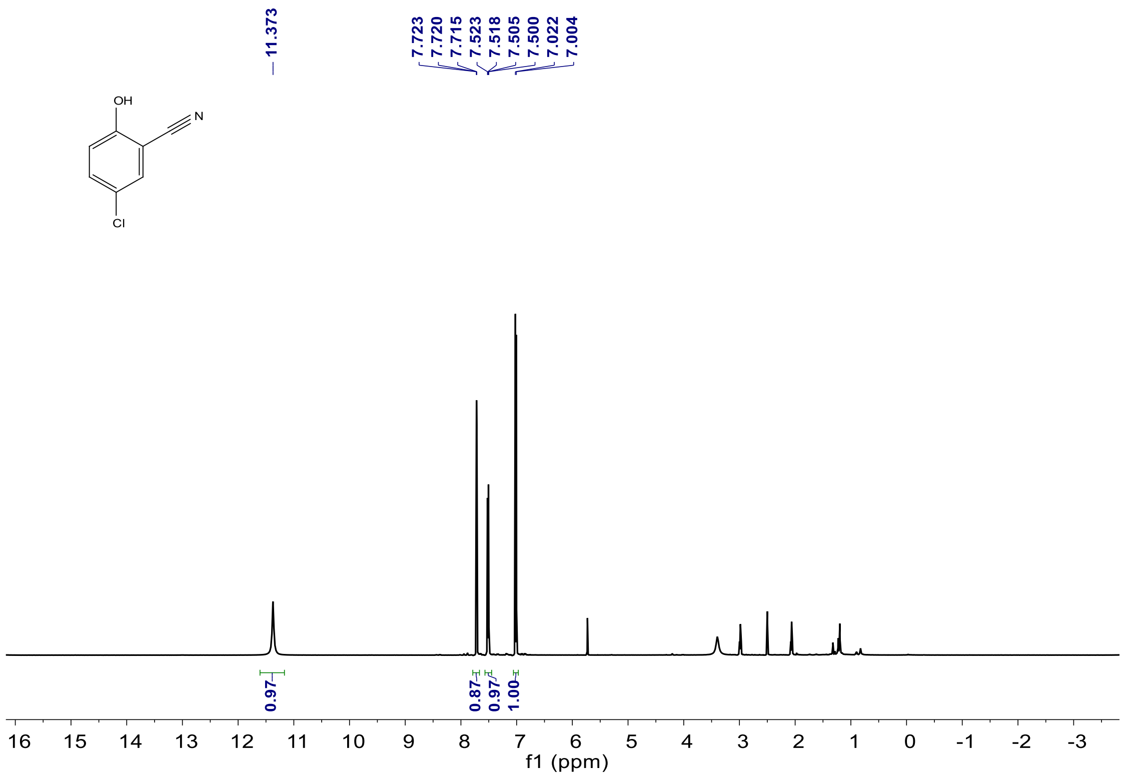

2o [13]**C NMR** (125 MHz, $CDCl_3$)

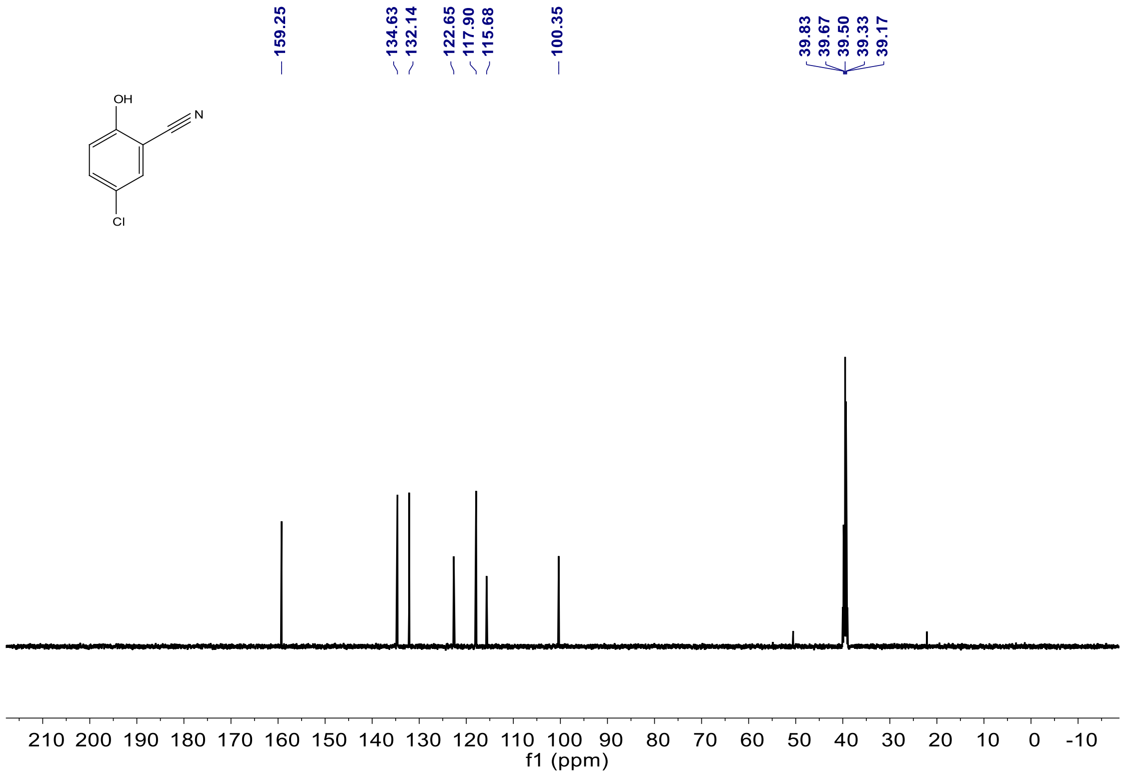

2p (**^{1}H NMR**, 500 MHz, $CDCl_3$)

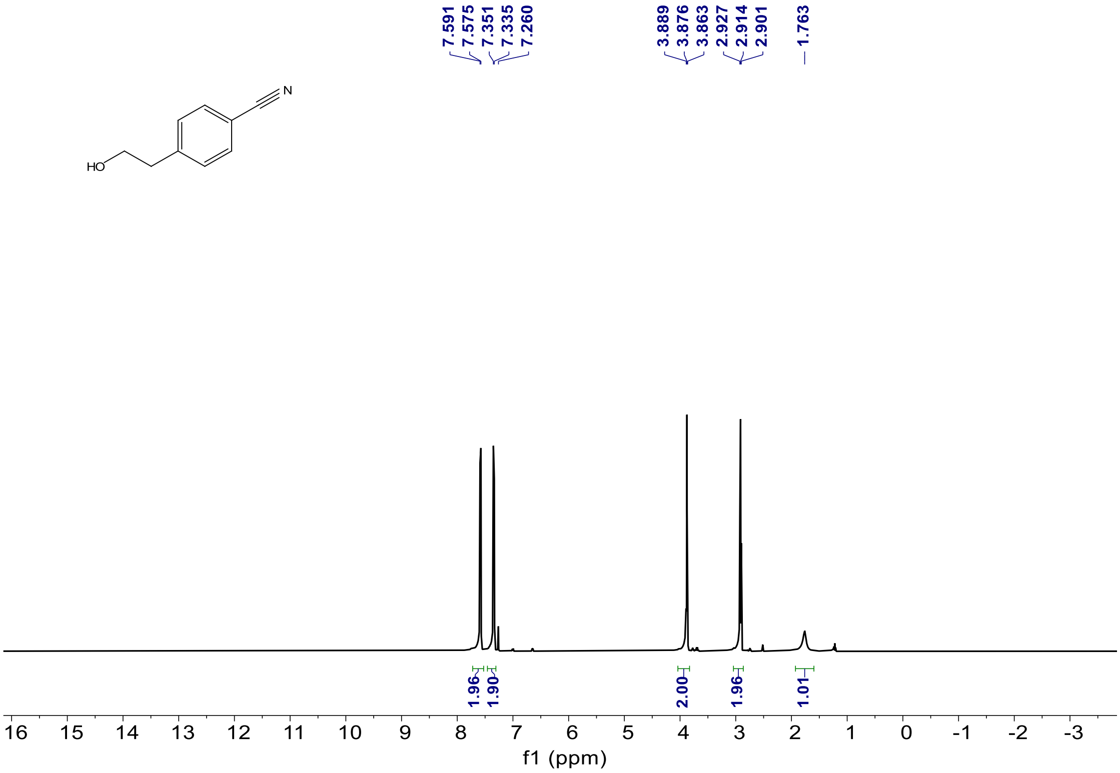

2p ^{13}**C NMR** (125 MHz, $CDCl_3$)

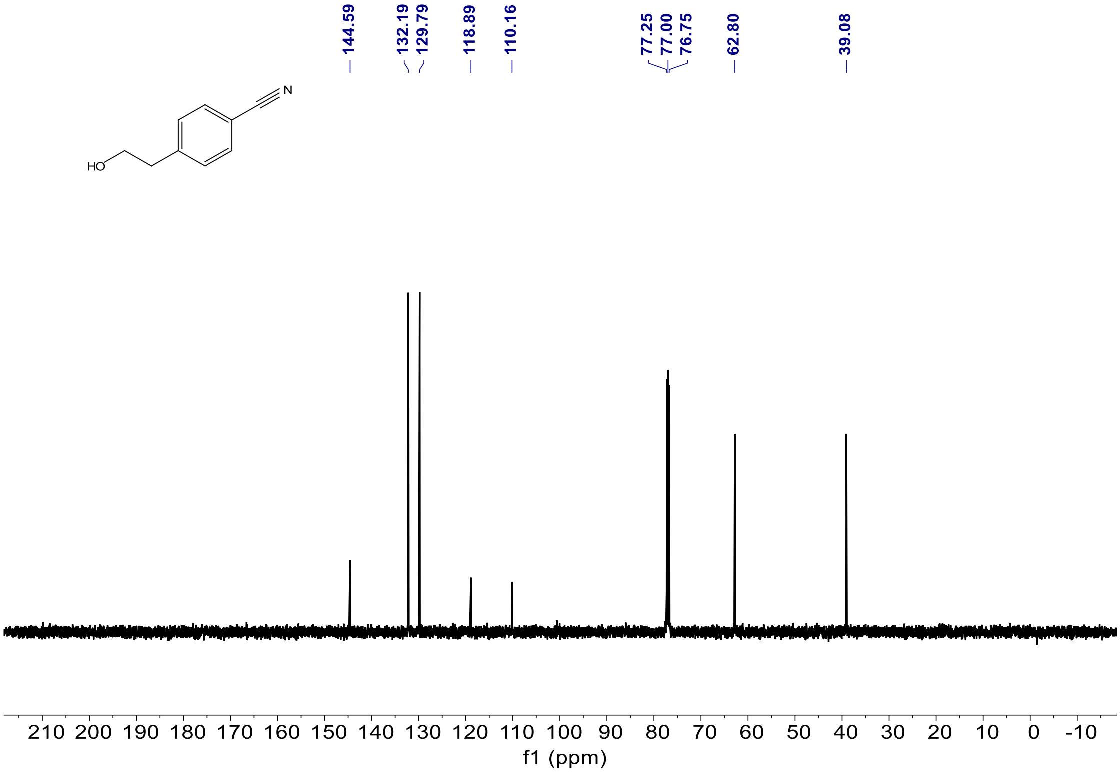

2q (**^{1}H NMR**, 500 MHz, $CDCl_3$)

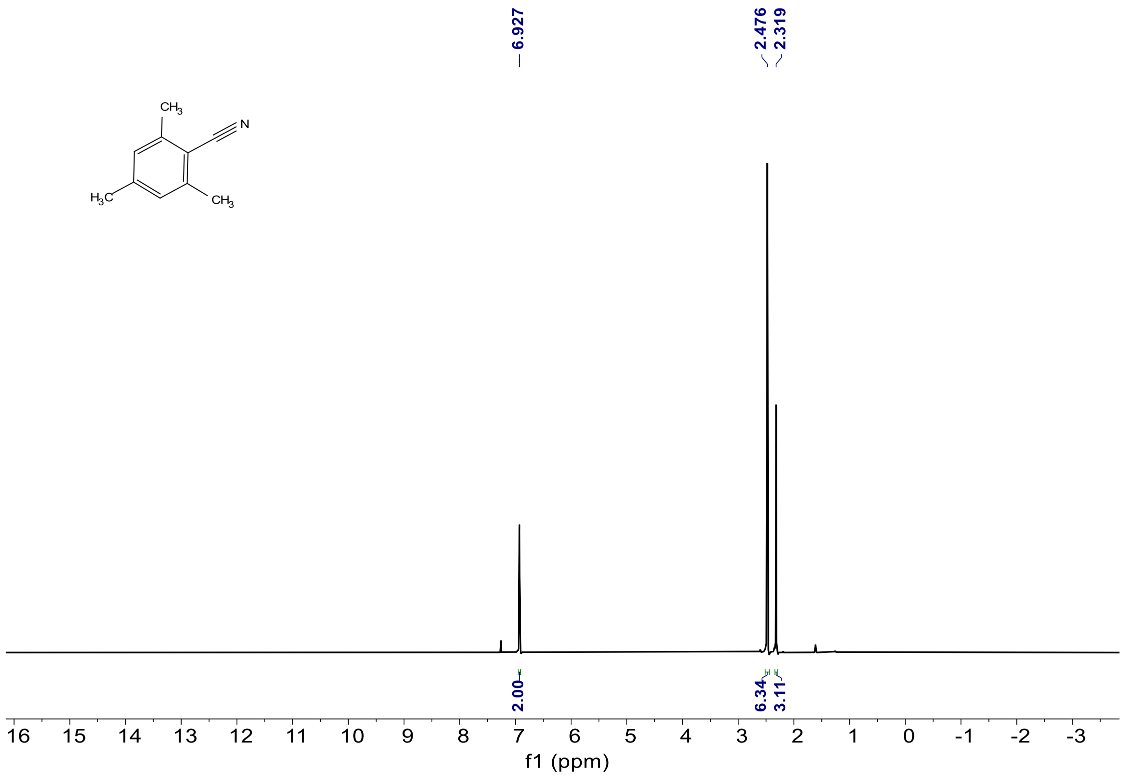

2q ^{13}C NMR (125 MHz, $CDCl_3$)

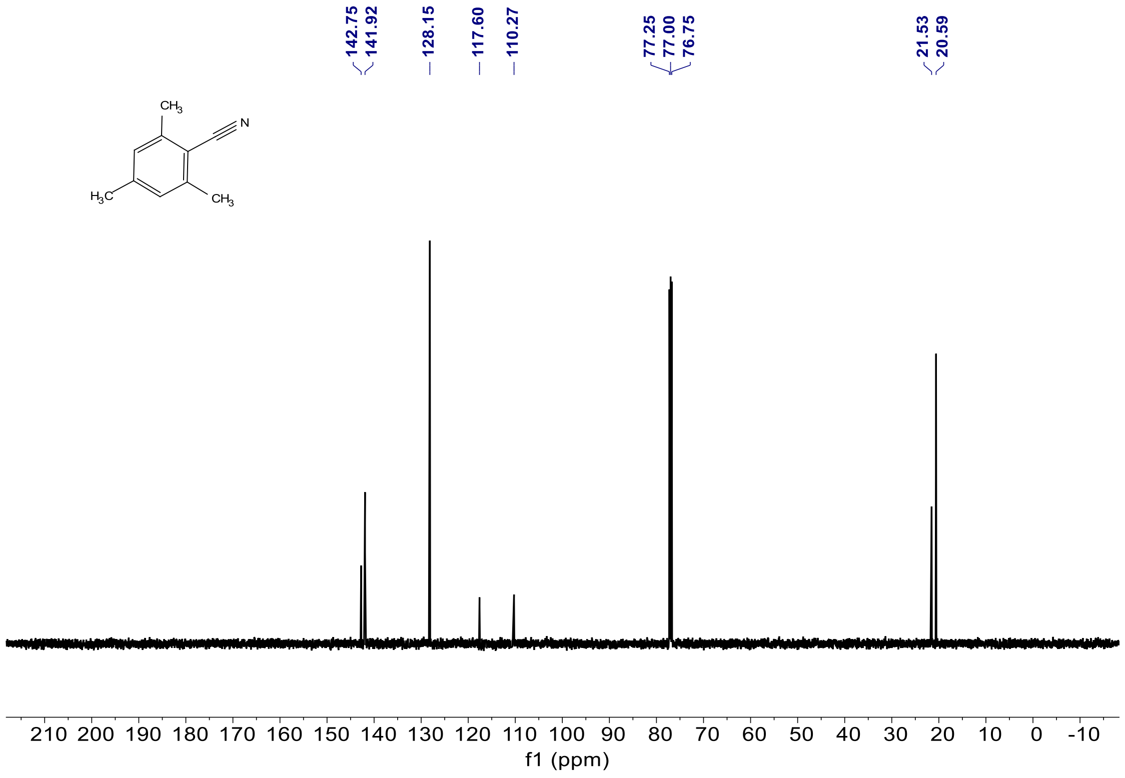

2r (**¹H NMR**, 500 MHz, $CDCl_3$)

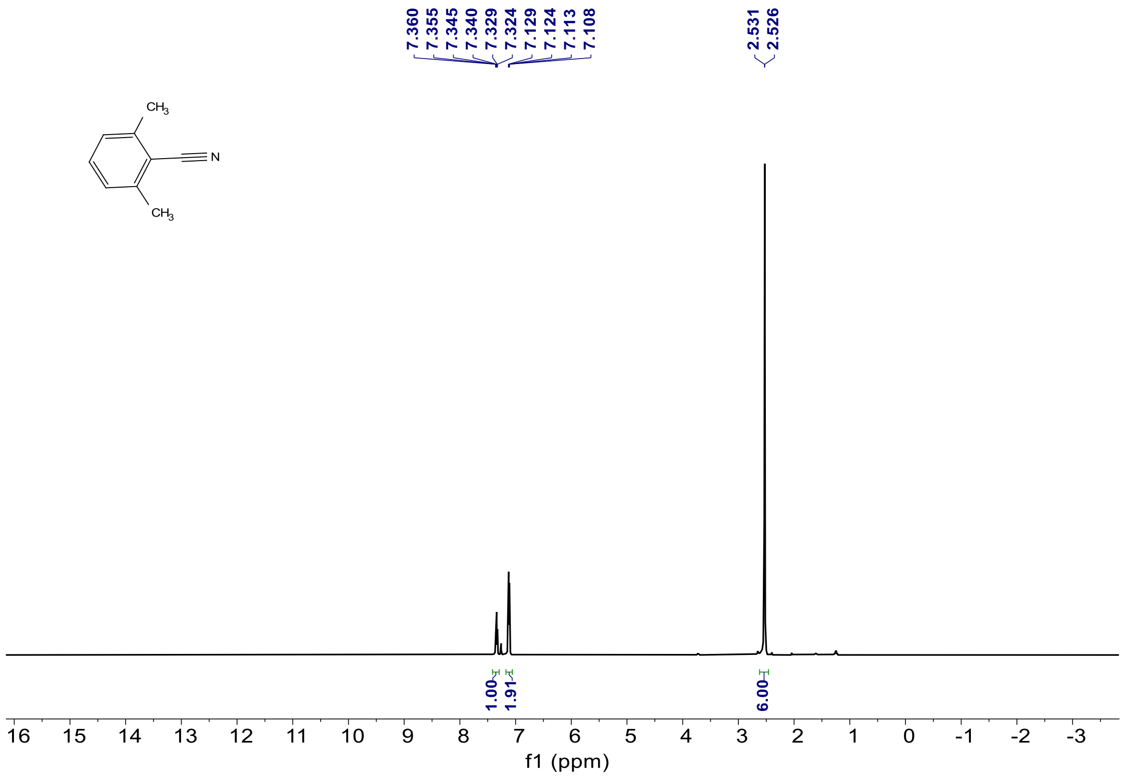

2r **¹³C NMR** (125 MHz, $CDCl_3$)

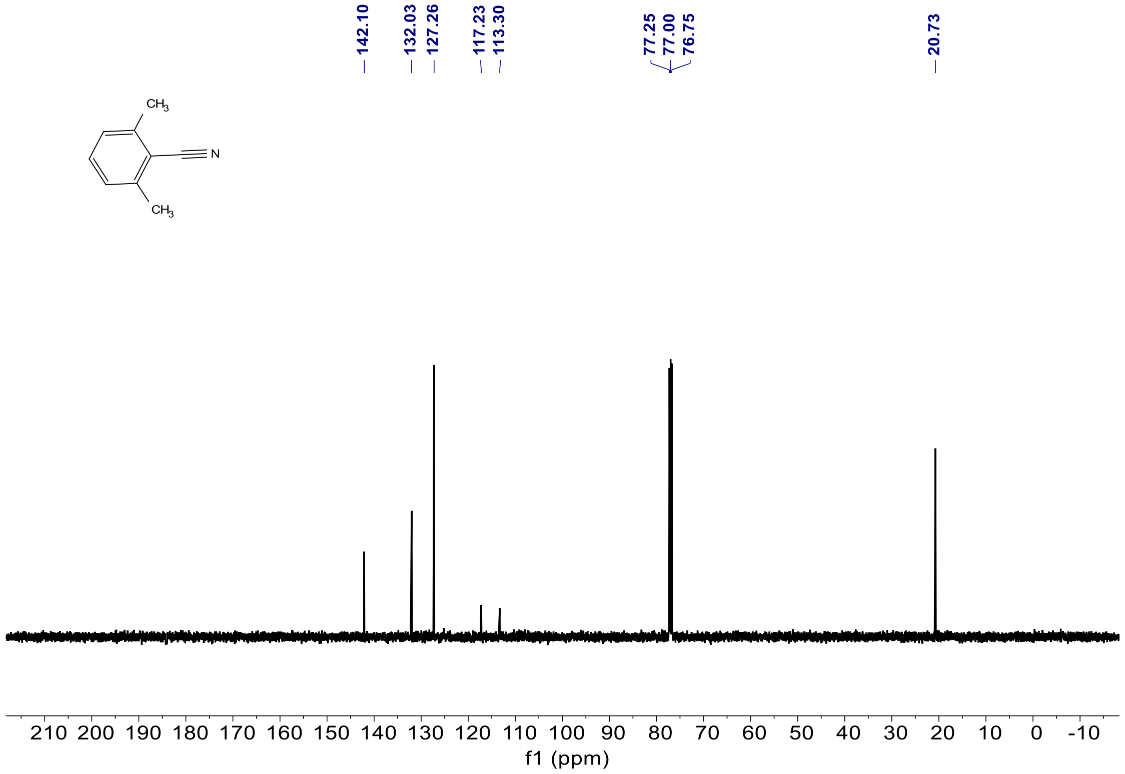

2s (**¹H NMR**, 400 MHz, $CDCl_3$)

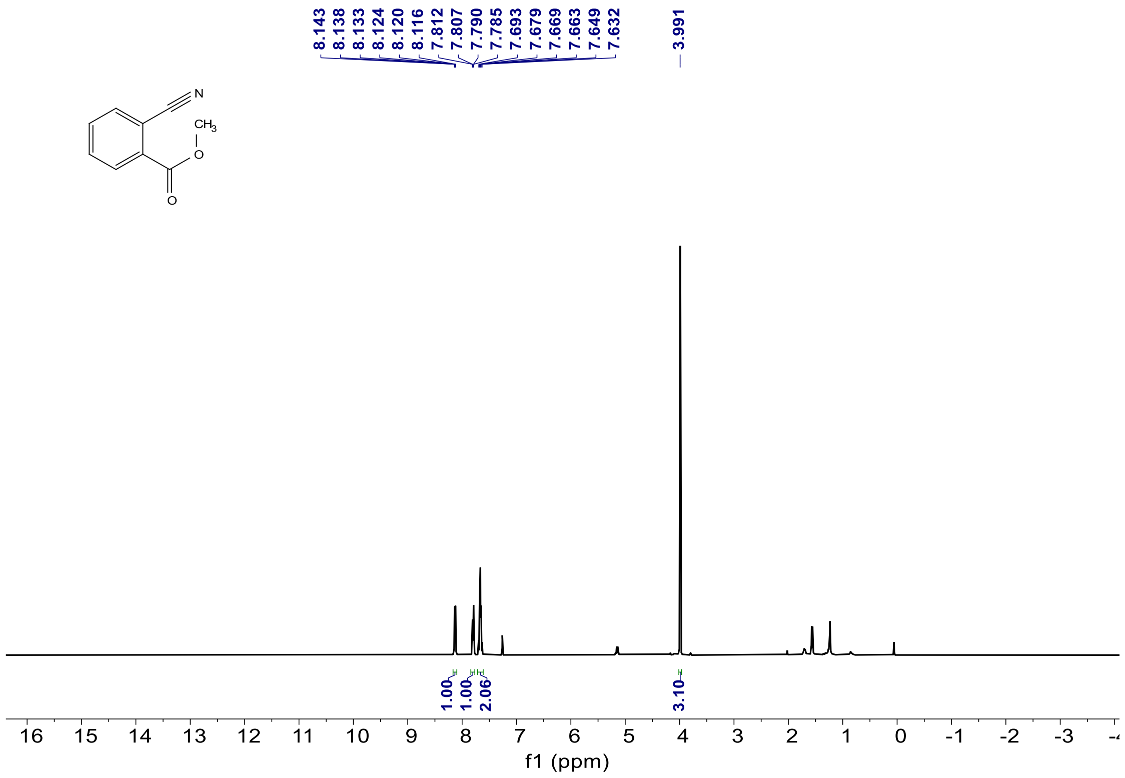

2s **¹³C NMR** (100 MHz, $CDCl_3$)

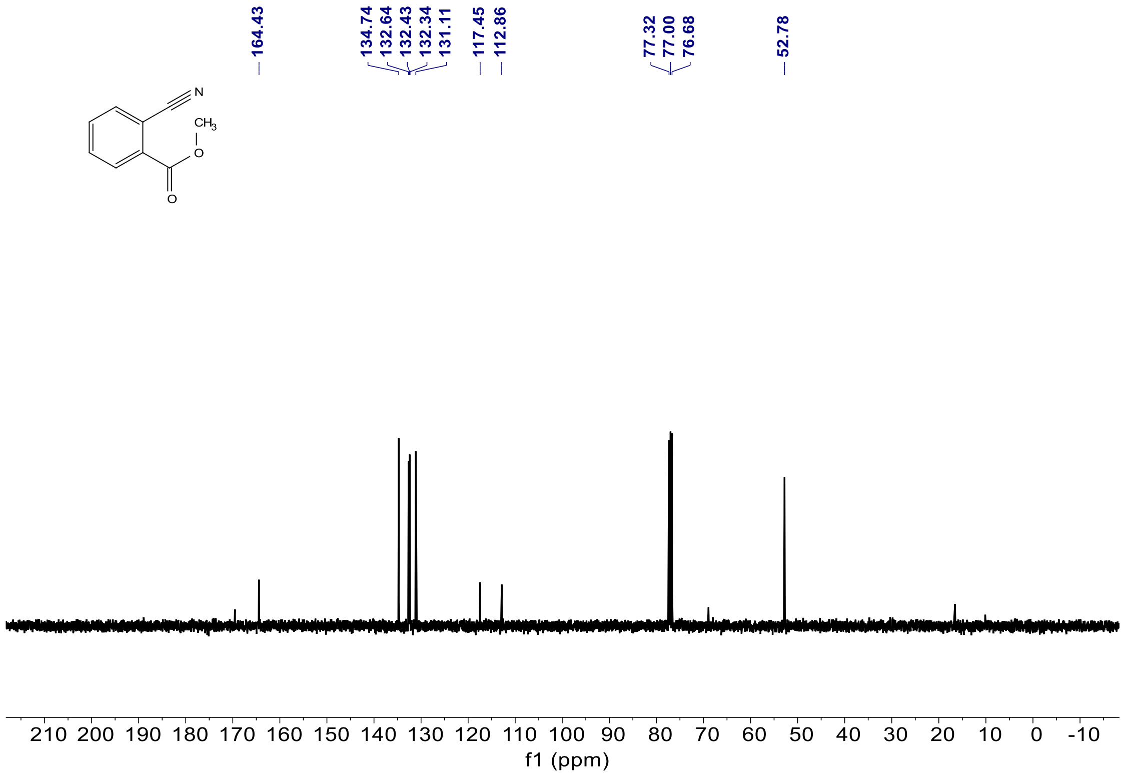

2t (^{1}**H NMR**, 400 MHz, $CDCl_3$)

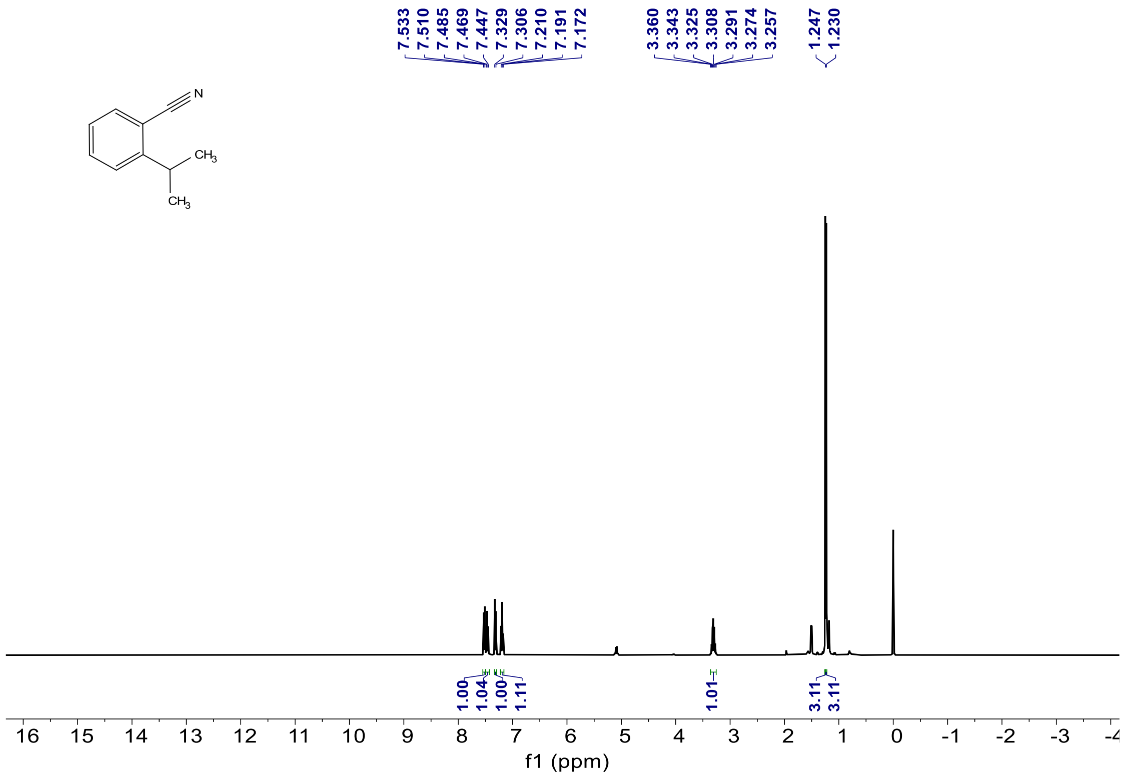

2t ^{13}**C NMR** (100 MHz, $CDCl_3$)

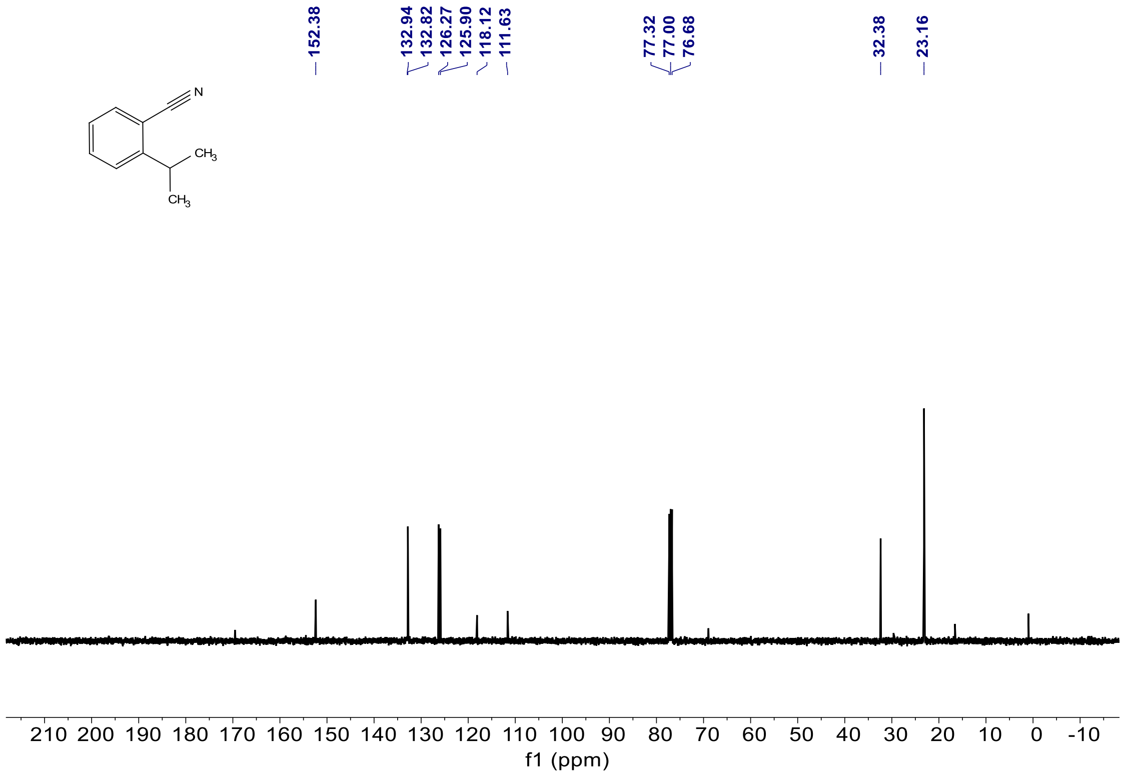

2u ([1]**H NMR**, 500 MHz, $CDCl_3$)

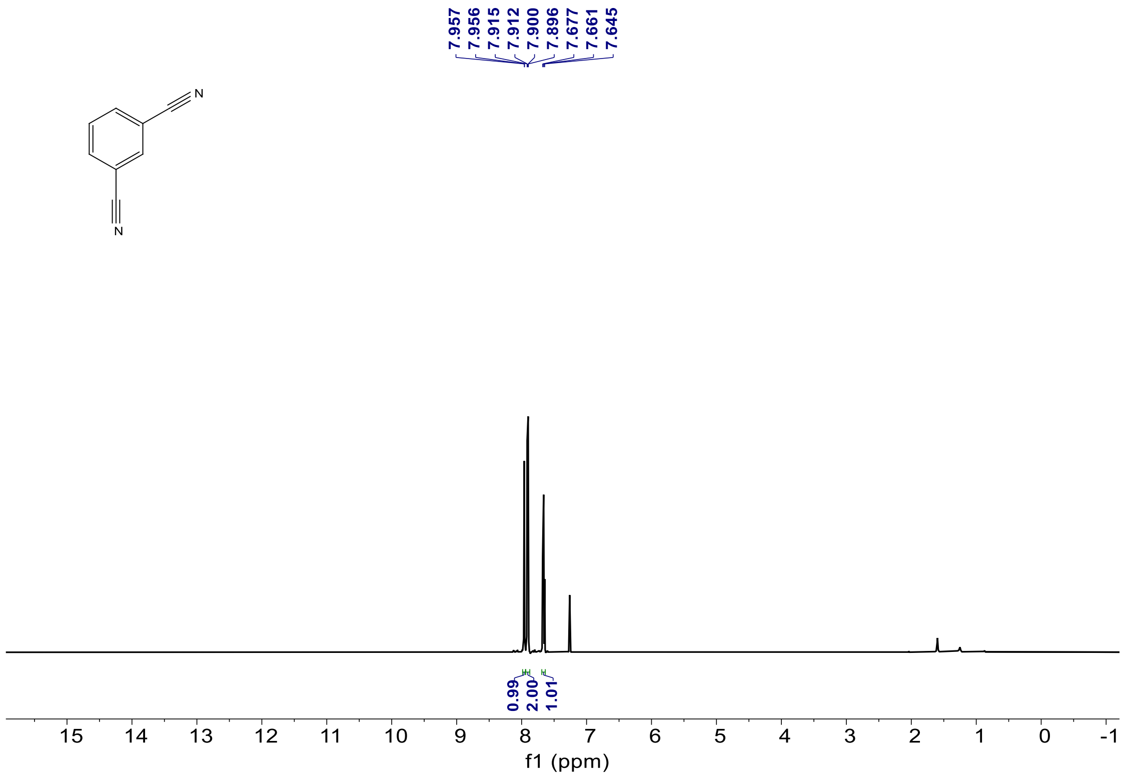

2u [13]**C NMR** (125 MHz, $CDCl_3$)

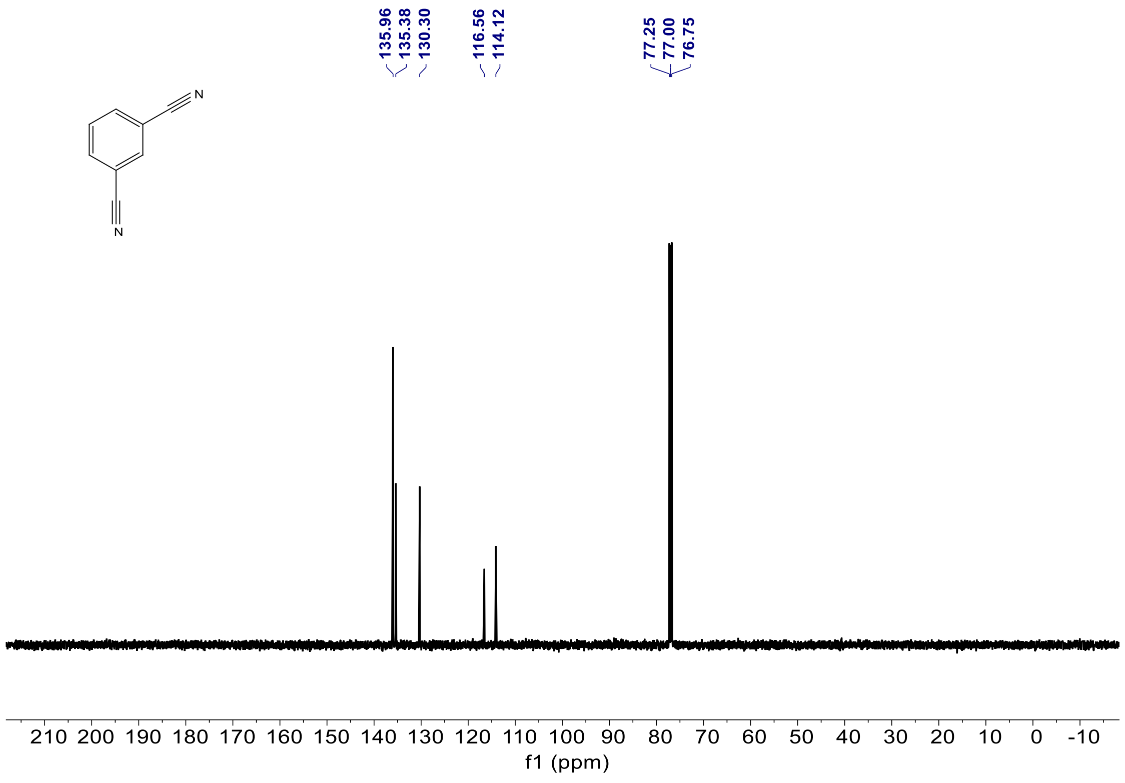

2v (**^{1}H NMR**, 400 MHz, d_6-DMSO)

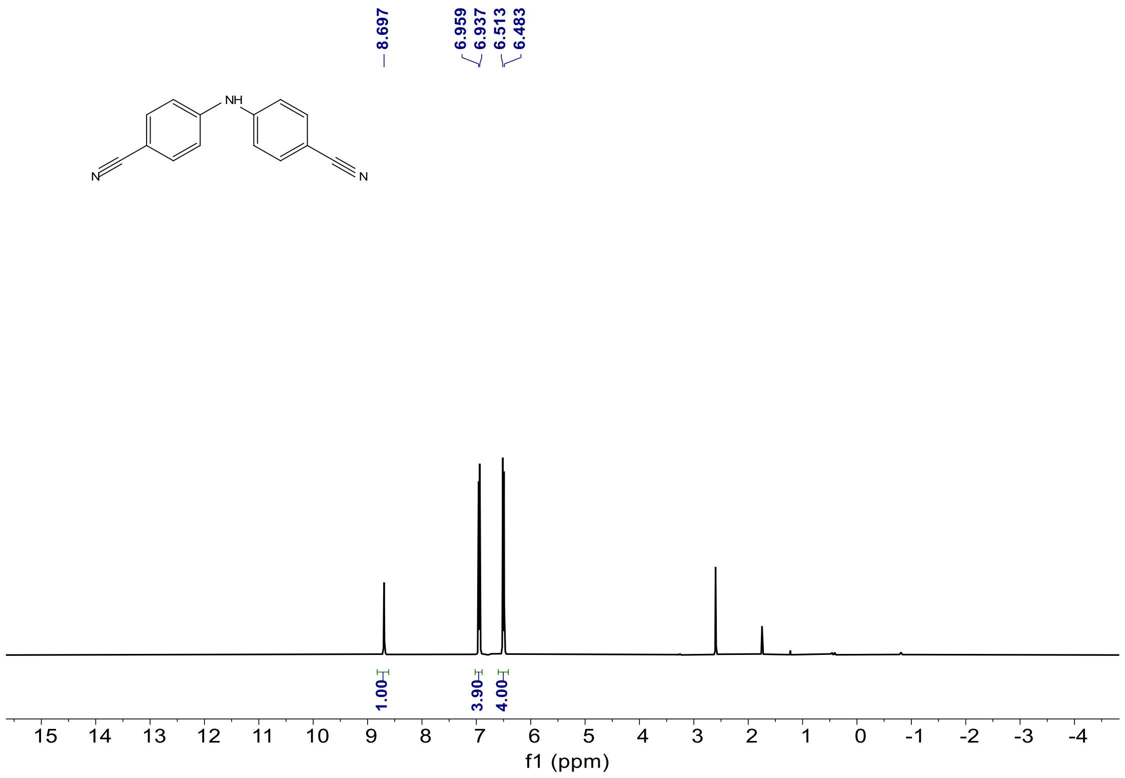

2v ^{13}C NMR (100 MHz, d_6-DMSO)

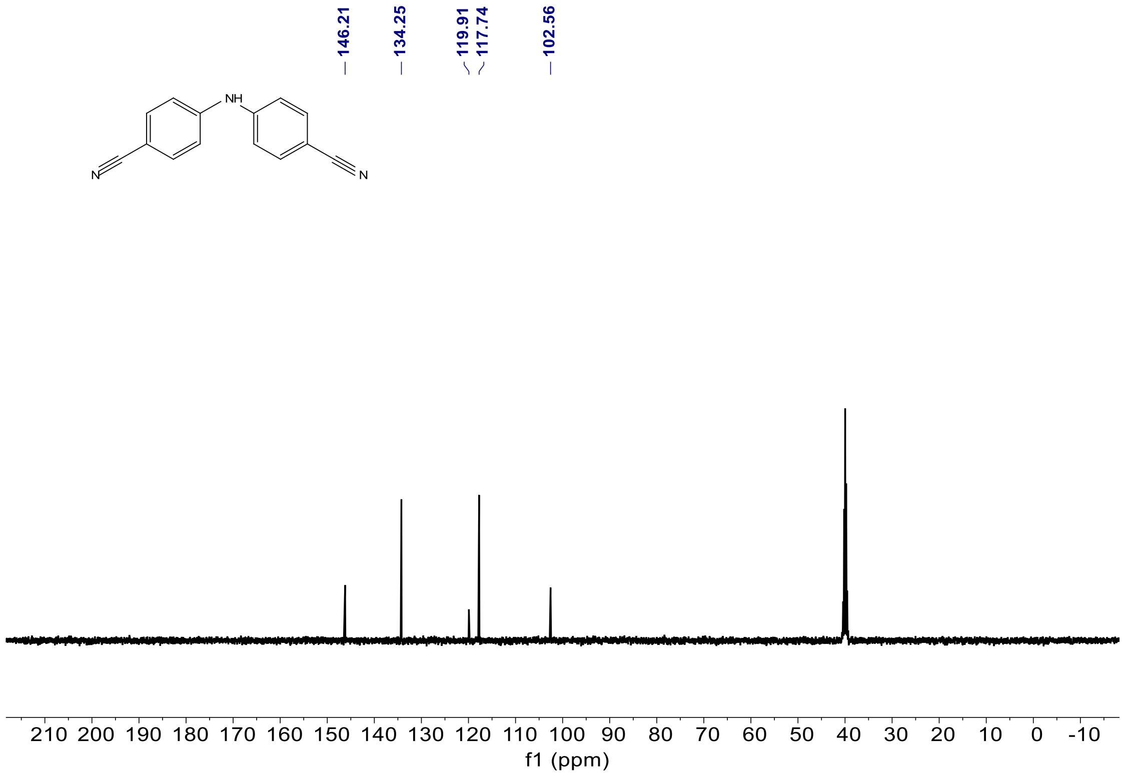

2w (^{1}**H NMR**, 500 MHz, $CDCl_3$)

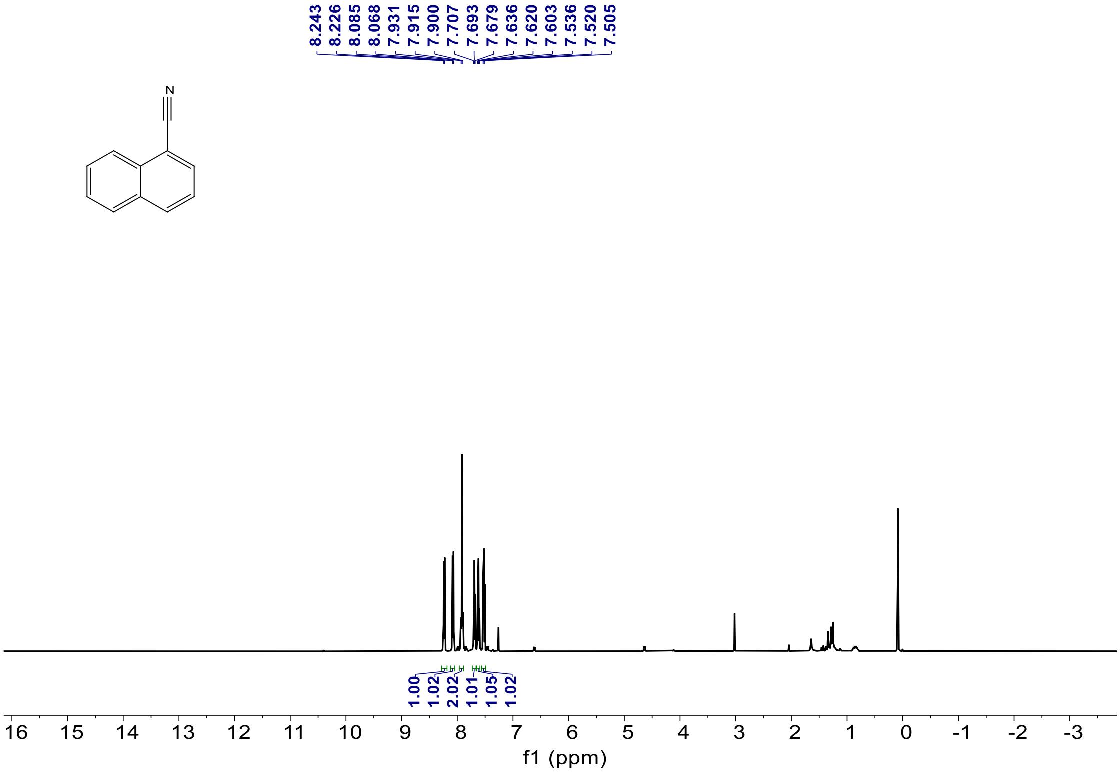

2w ^{13}**C NMR** (125 MHz, $CDCl_3$)

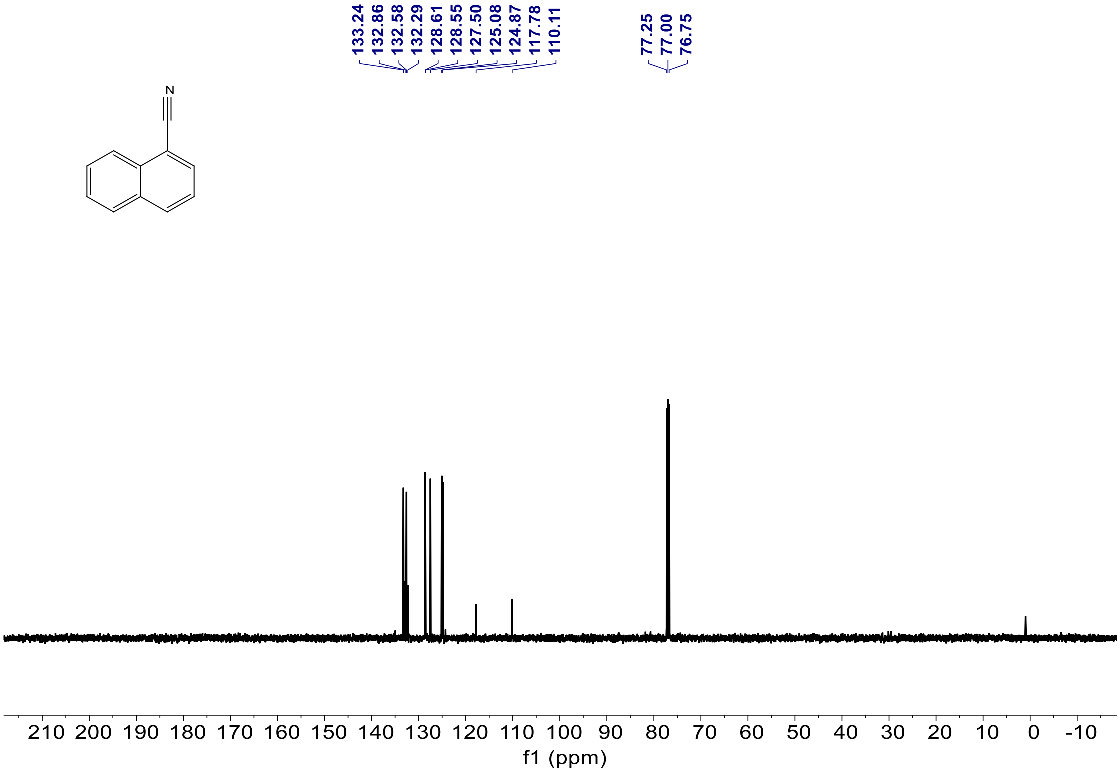

2x (**^{1}H NMR**, 500 MHz, $CDCl_3$)

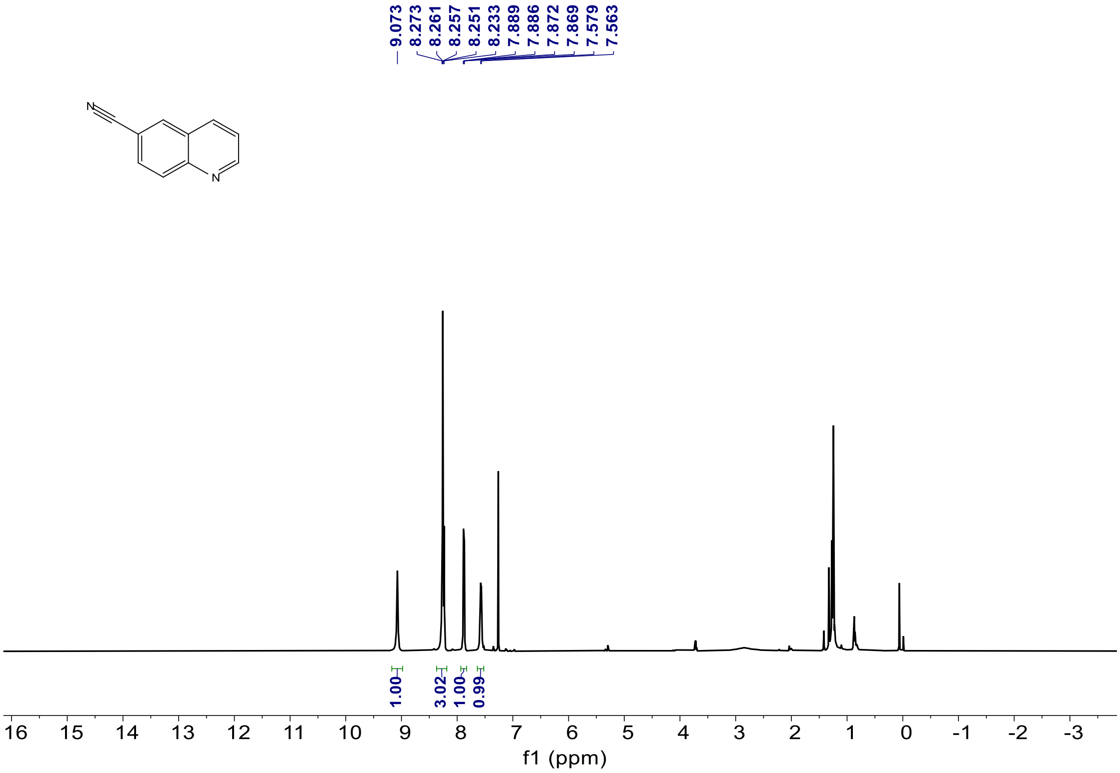

2x **^{13}C NMR** (125 MHz, $CDCl_3$)

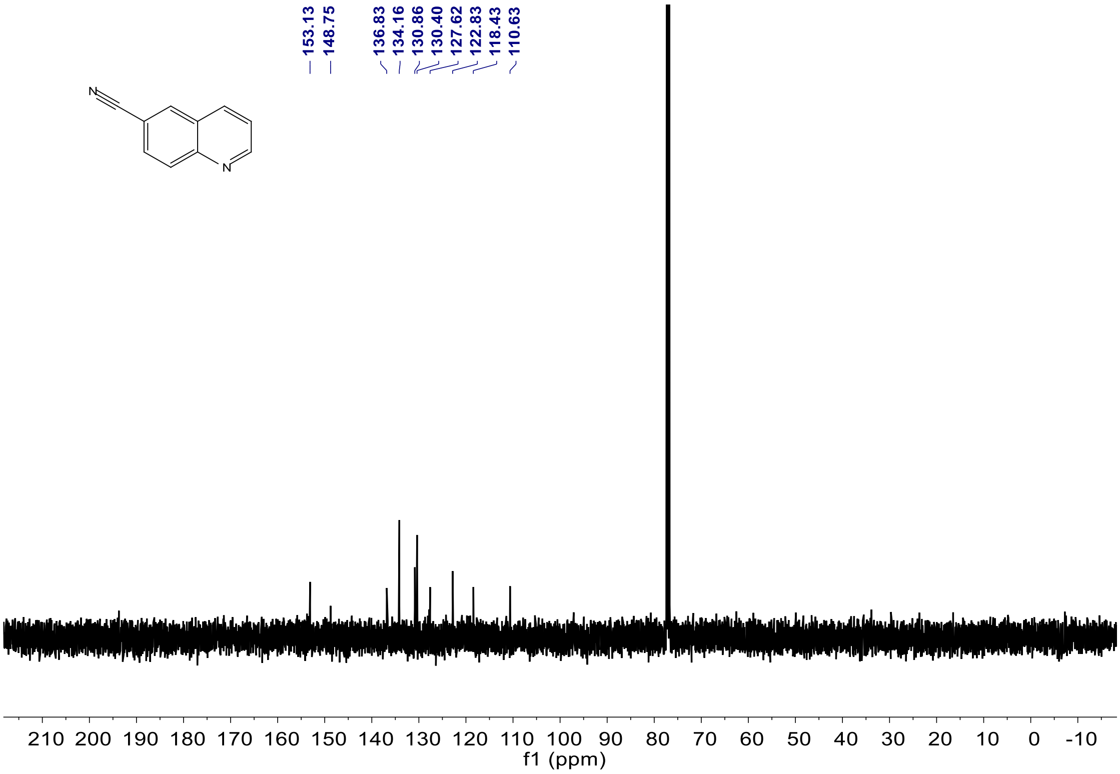

2y ([1]**H NMR**, 500 MHz, $CDCl_3$)

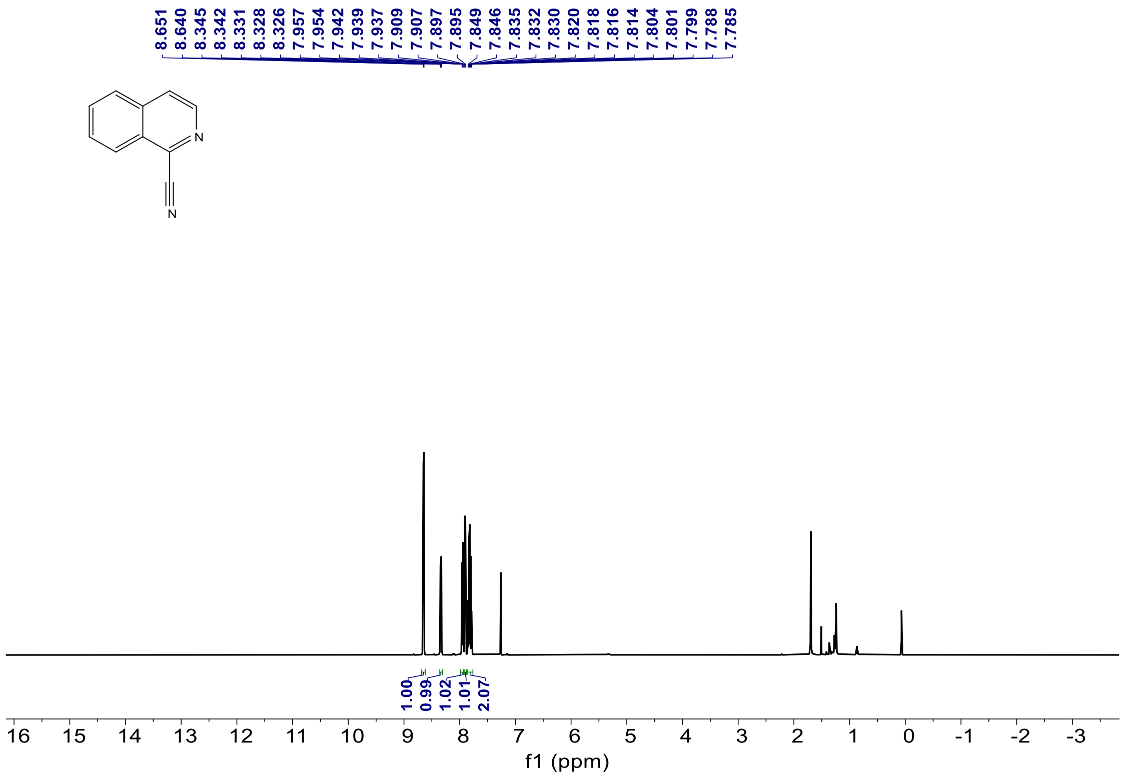

2y [13]**C NMR** (125 MHz, $CDCl_3$)

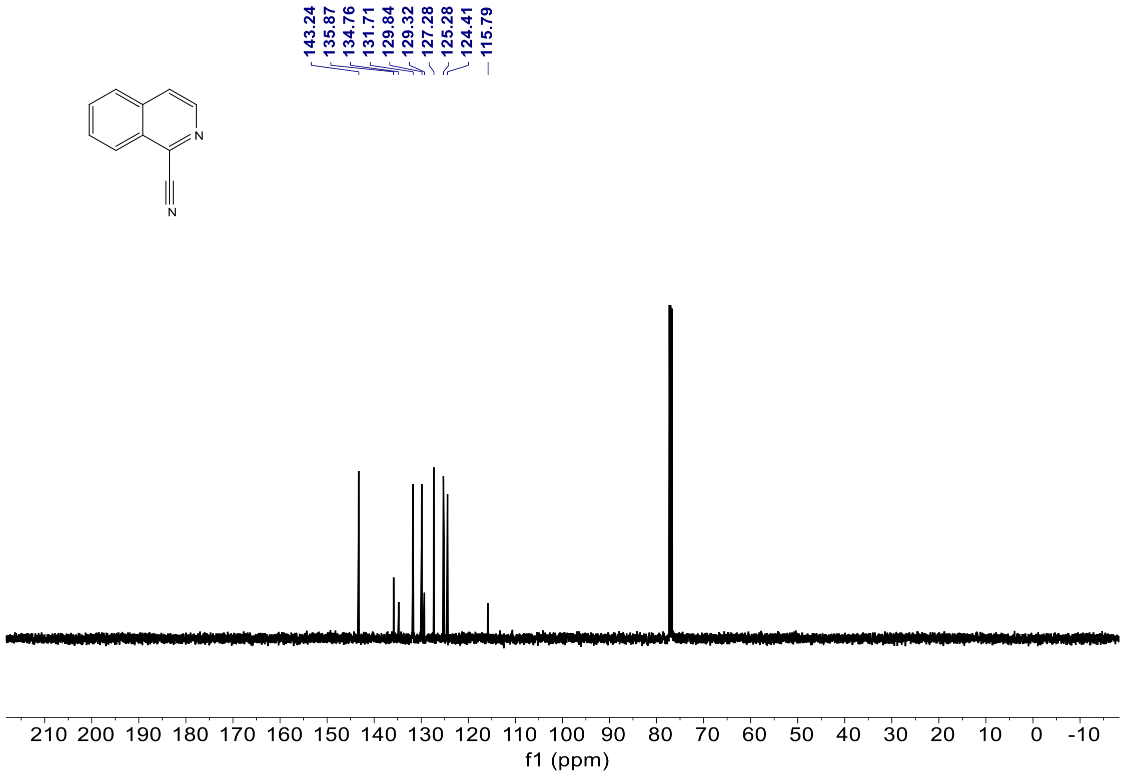

2z (**^{1}H NMR**, 500 MHz, $CDCl_3$)

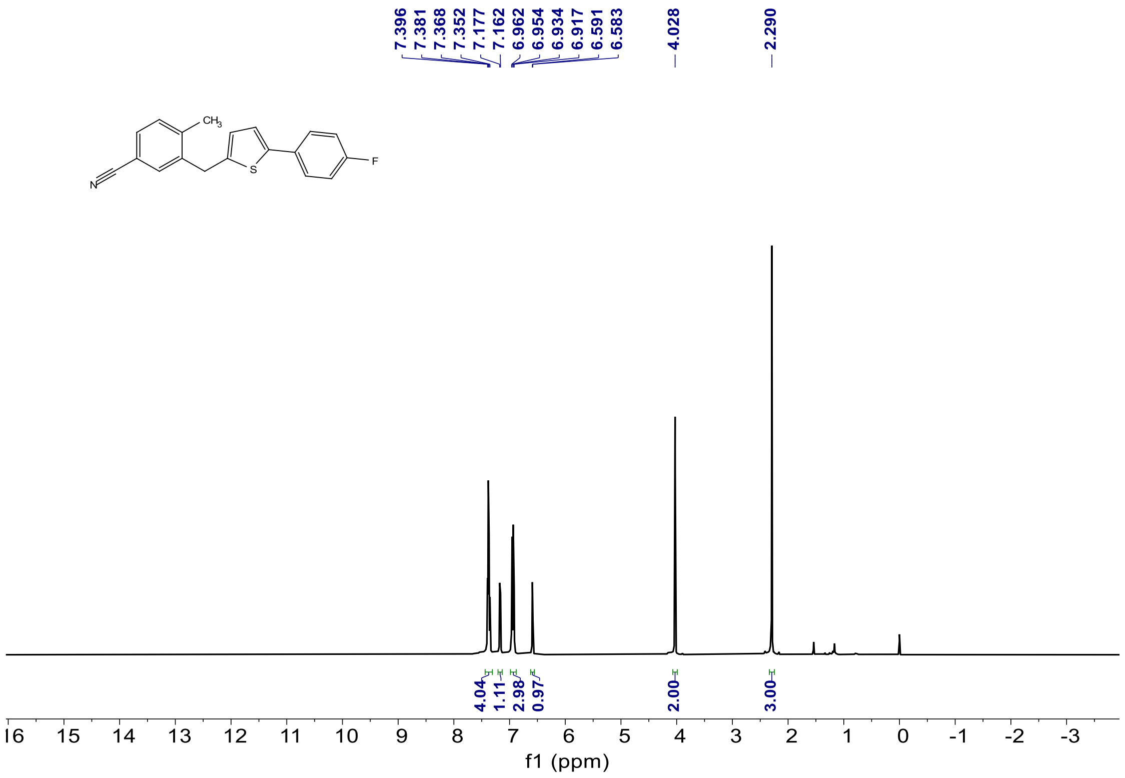

2z ^{13}C NMR (125 MHz, $CDCl_3$)

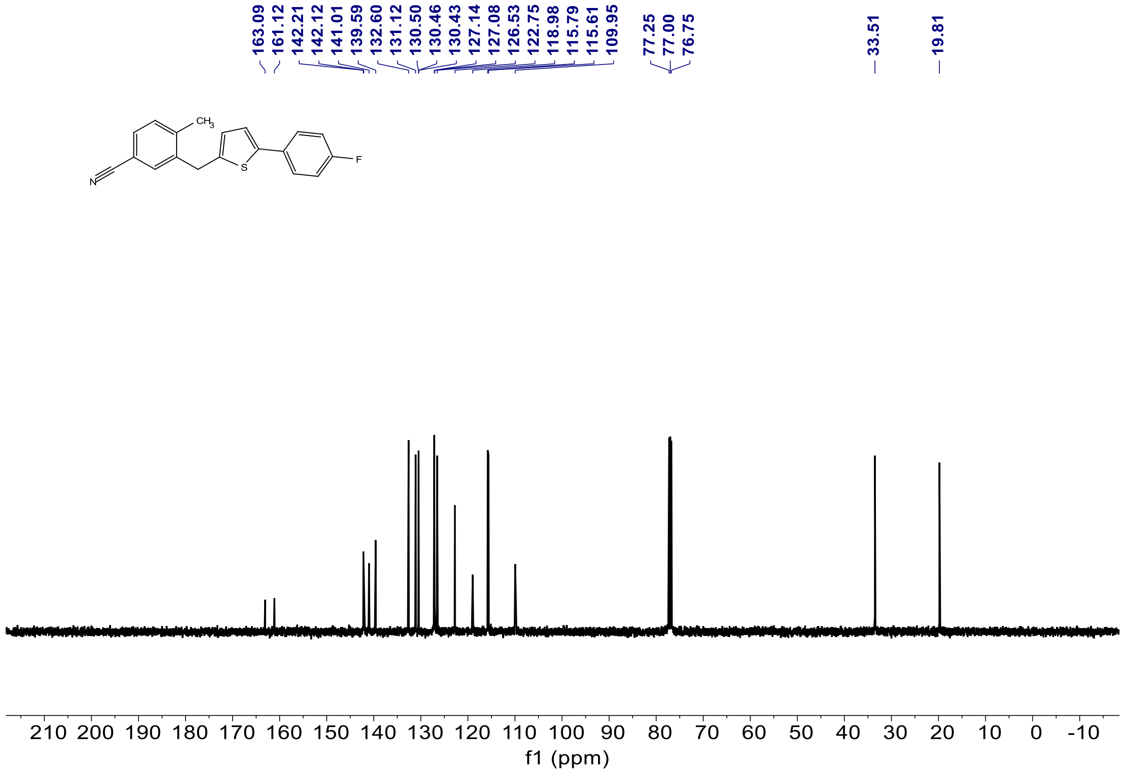

2a’ (**^{1}H NMR**, 500 MHz, $CDCl_3$)

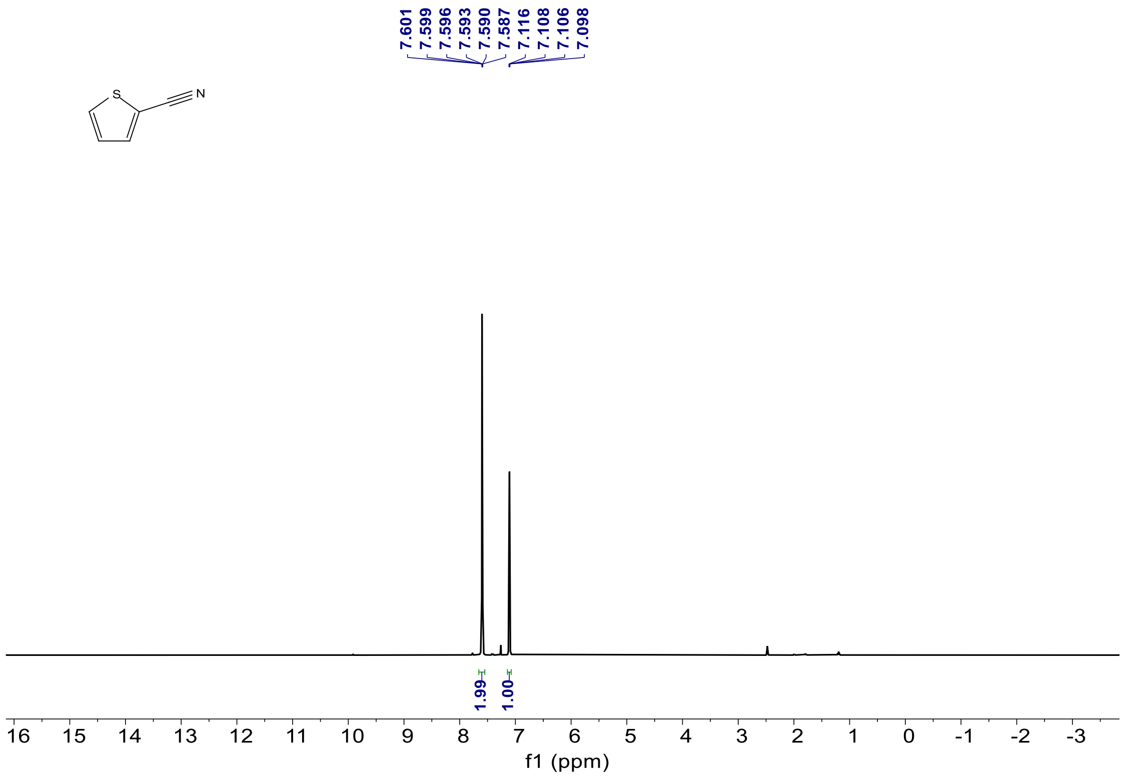

2a’ ^{13}C NMR (125 MHz, $CDCl_3$)

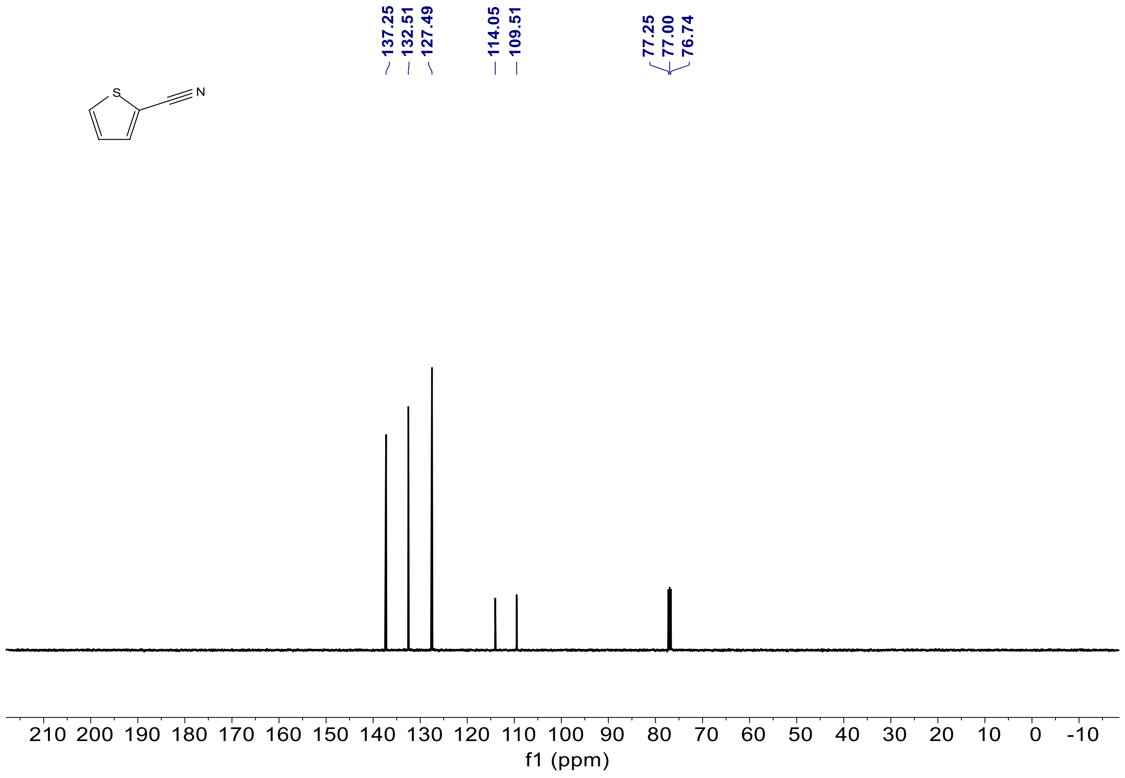

2b' (**^{1}H NMR**, 500 MHz, $CDCl_3$)

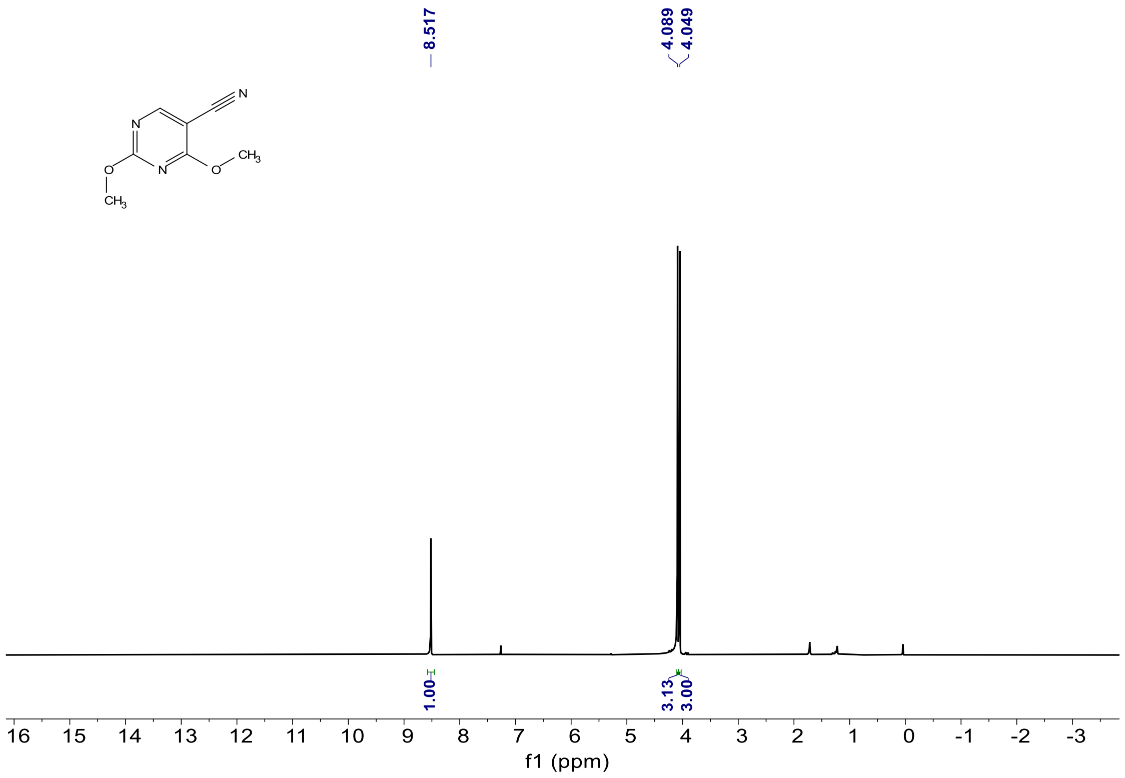

2b' ^{13}C NMR (125 MHz, $CDCl_3$)

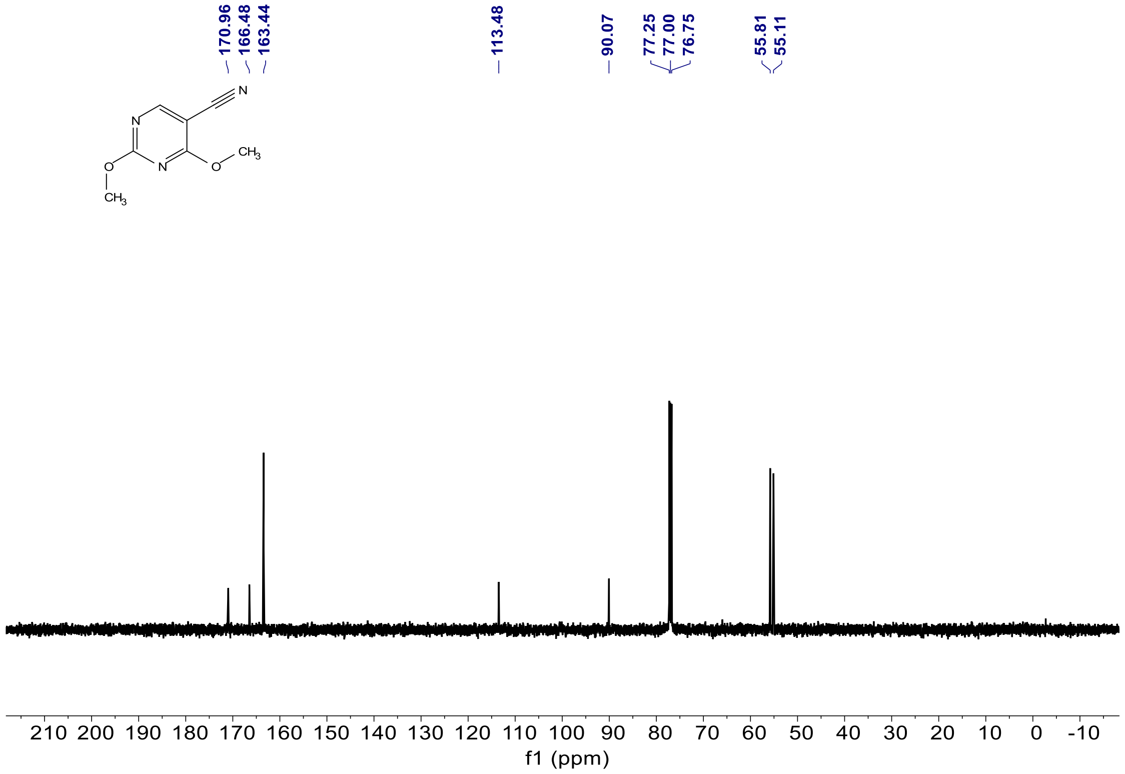

2c' (**^{1}H NMR**, 400 MHz, $CDCl_3$)

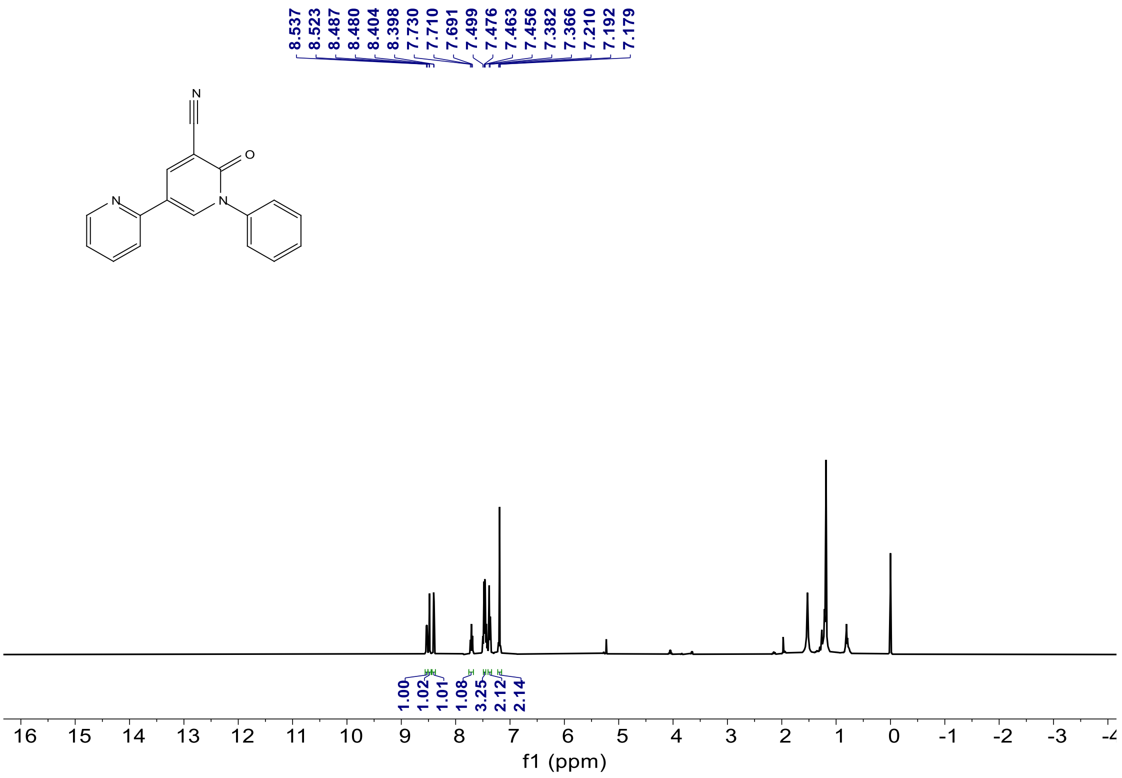

2c' ^{13}C NMR (100 MHz, $CDCl_3$)

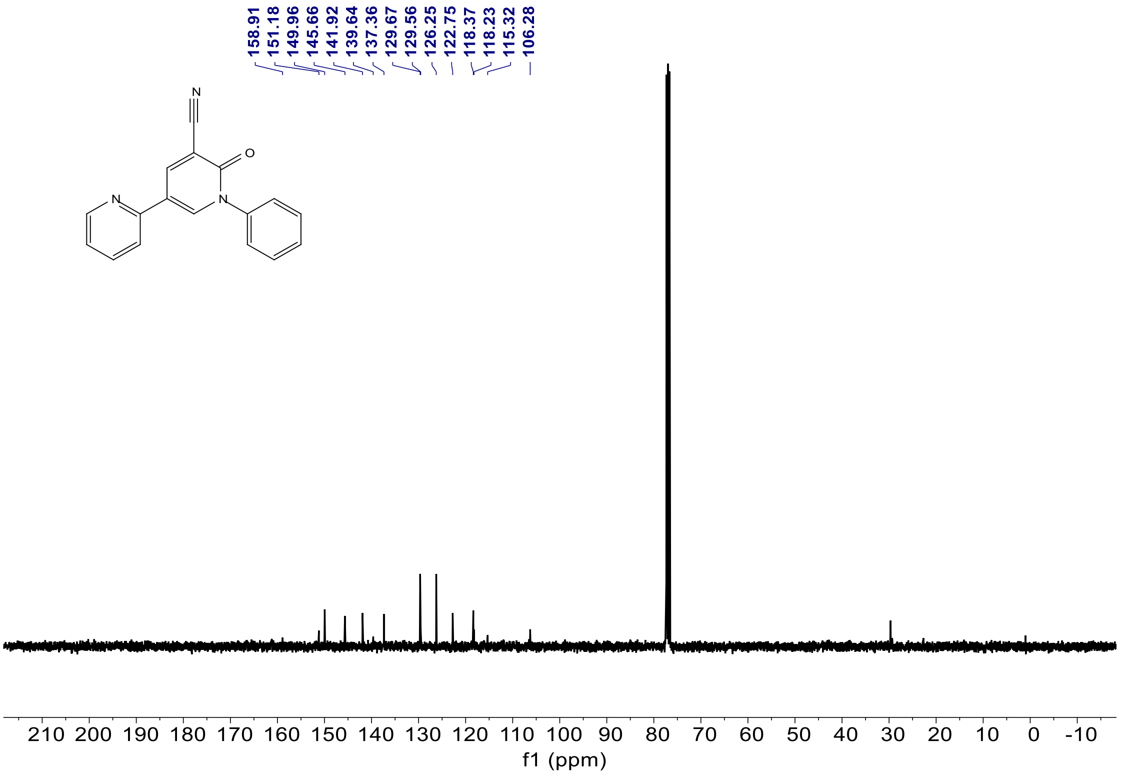

2d' (**^{1}H NMR**, 500 MHz, CDCl$_3$)

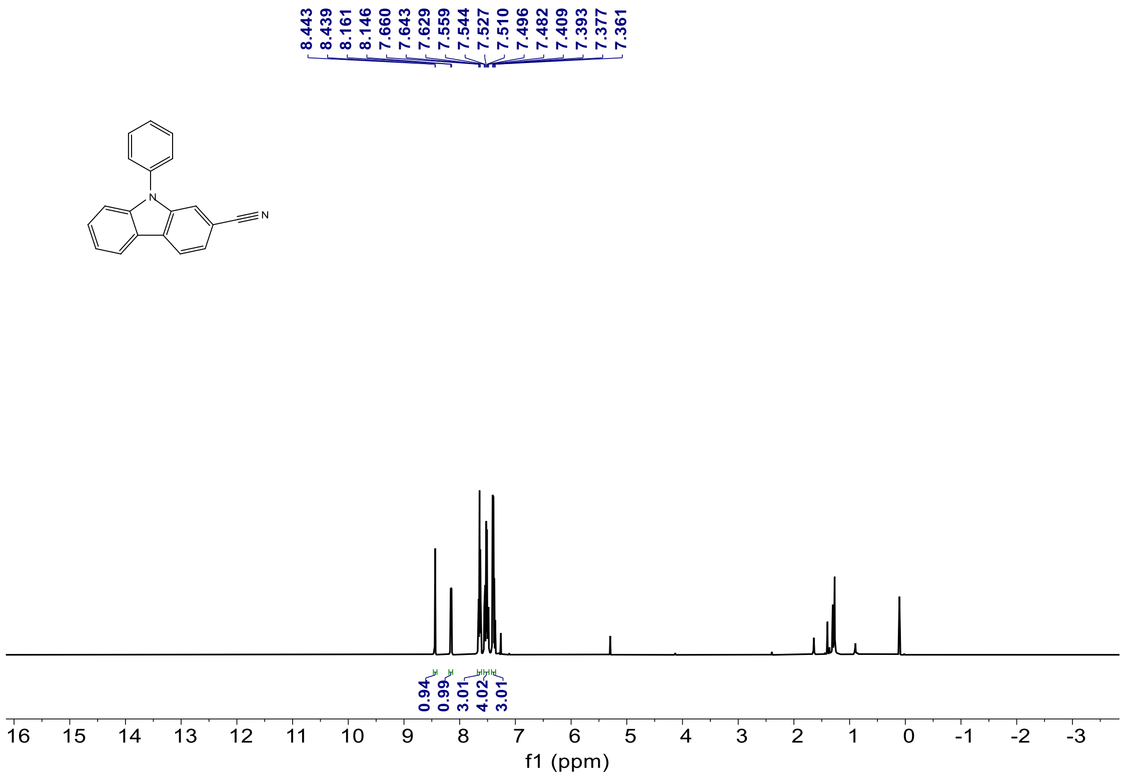

2d' ^{13}C NMR (125 MHz, CDCl$_3$)

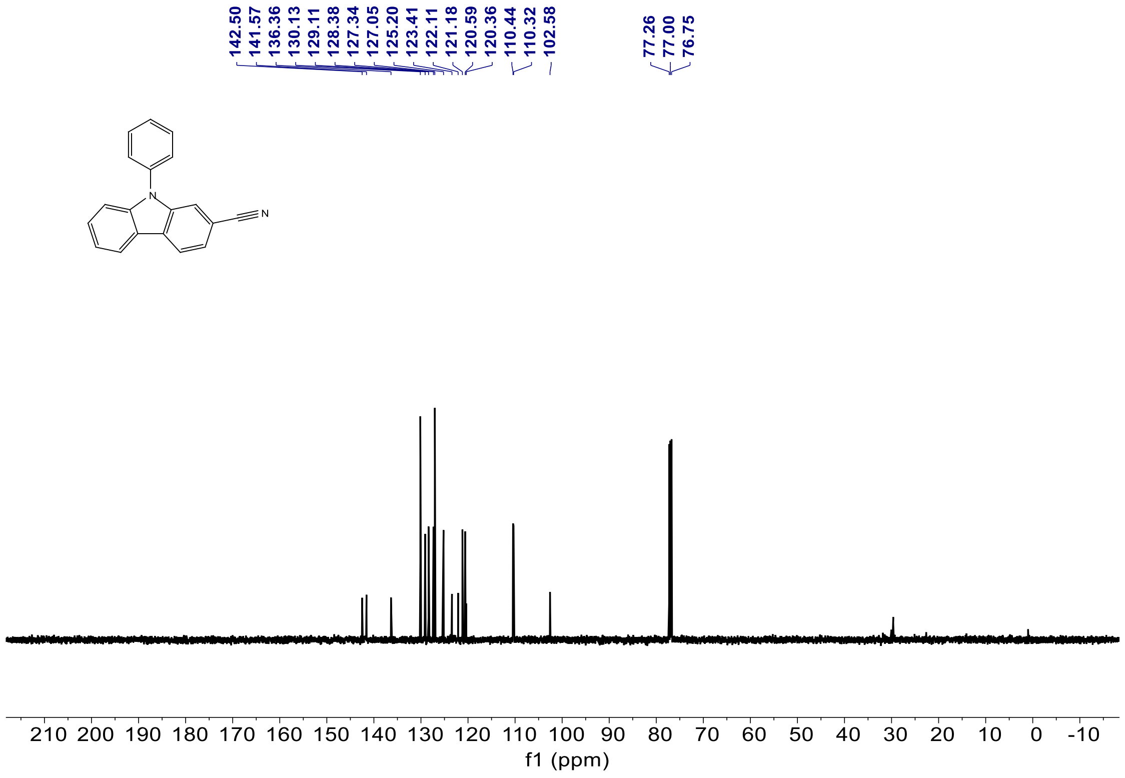

2e' ([1]**H NMR**, 500 MHz, CDCl3)

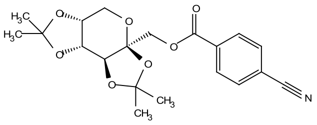

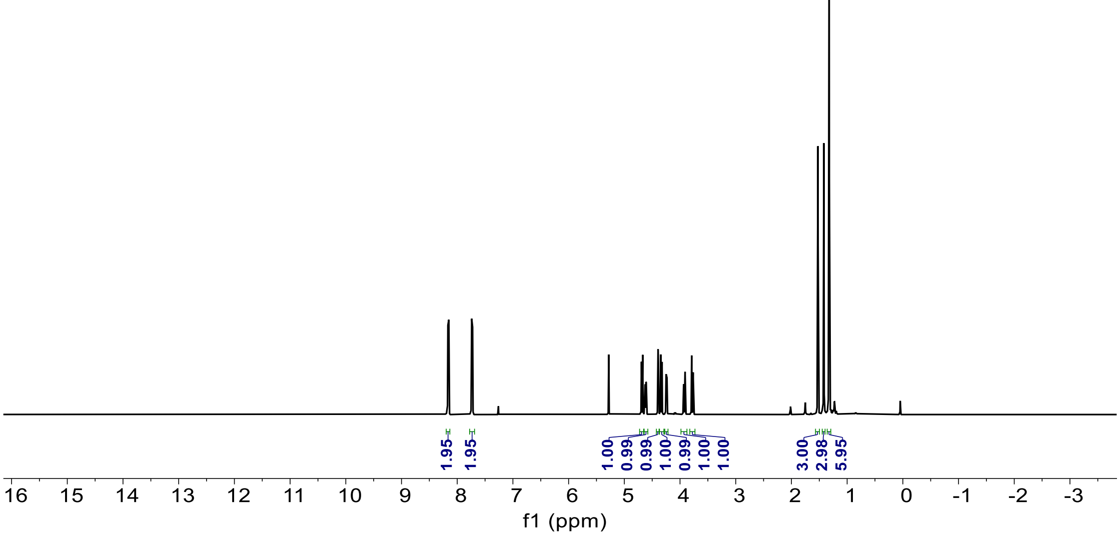

2e' [13]**C NMR** (125 MHz, CDCl3)

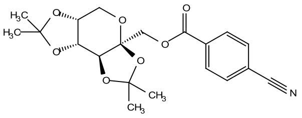

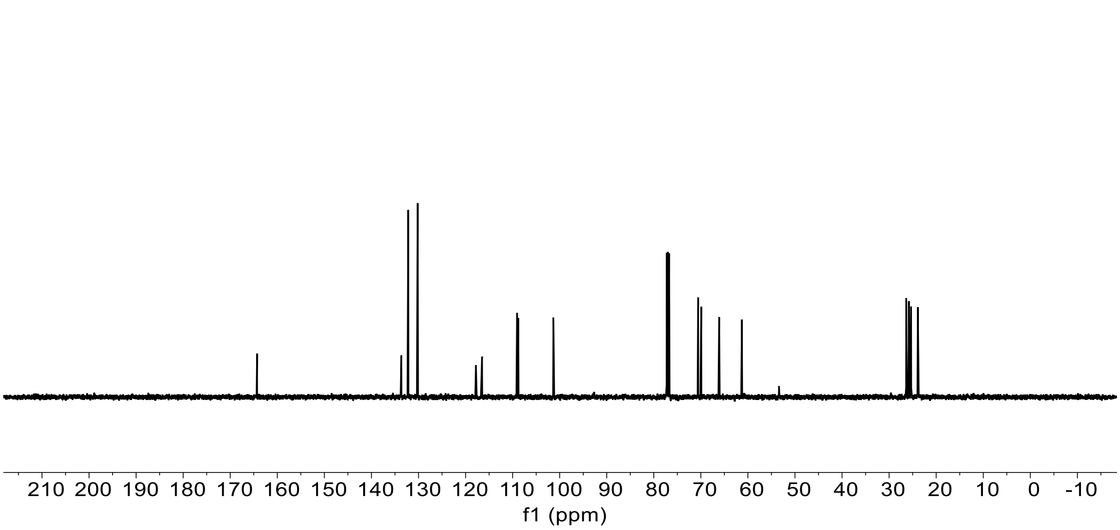

2f’ ([1]**H NMR**, 500 MHz, CDCl₃)

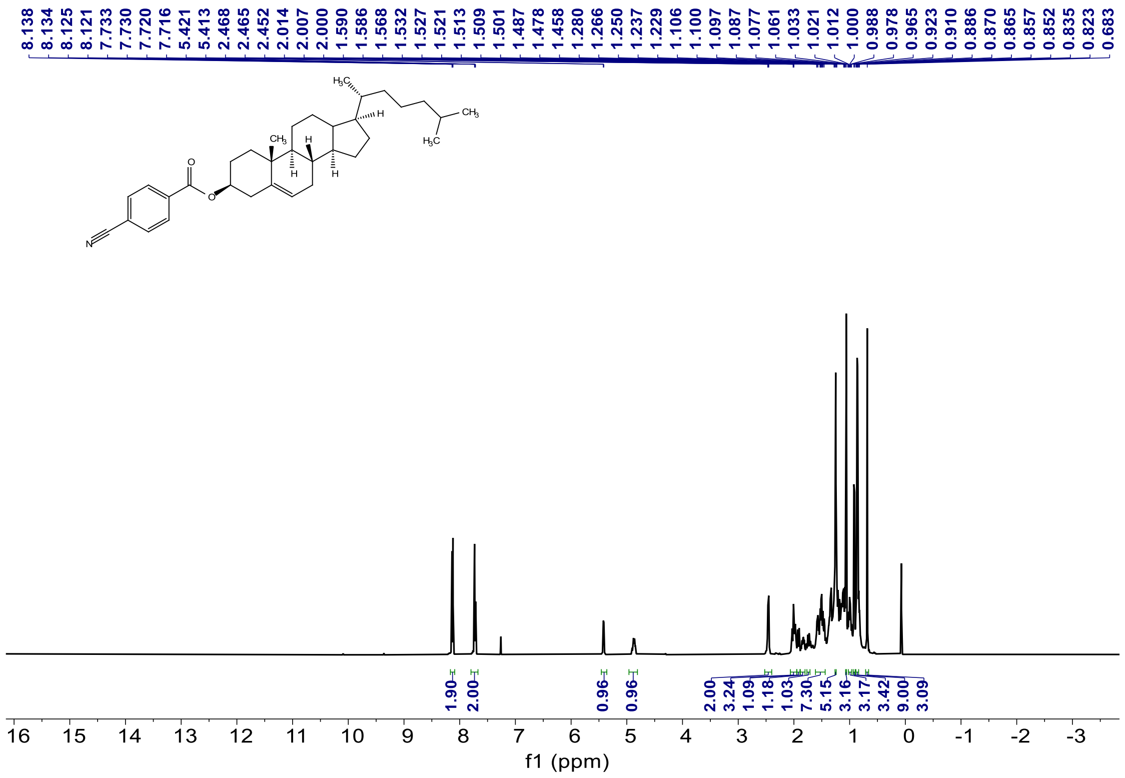

2f’ [13]**C NMR** (125 MHz, CDCl₃)

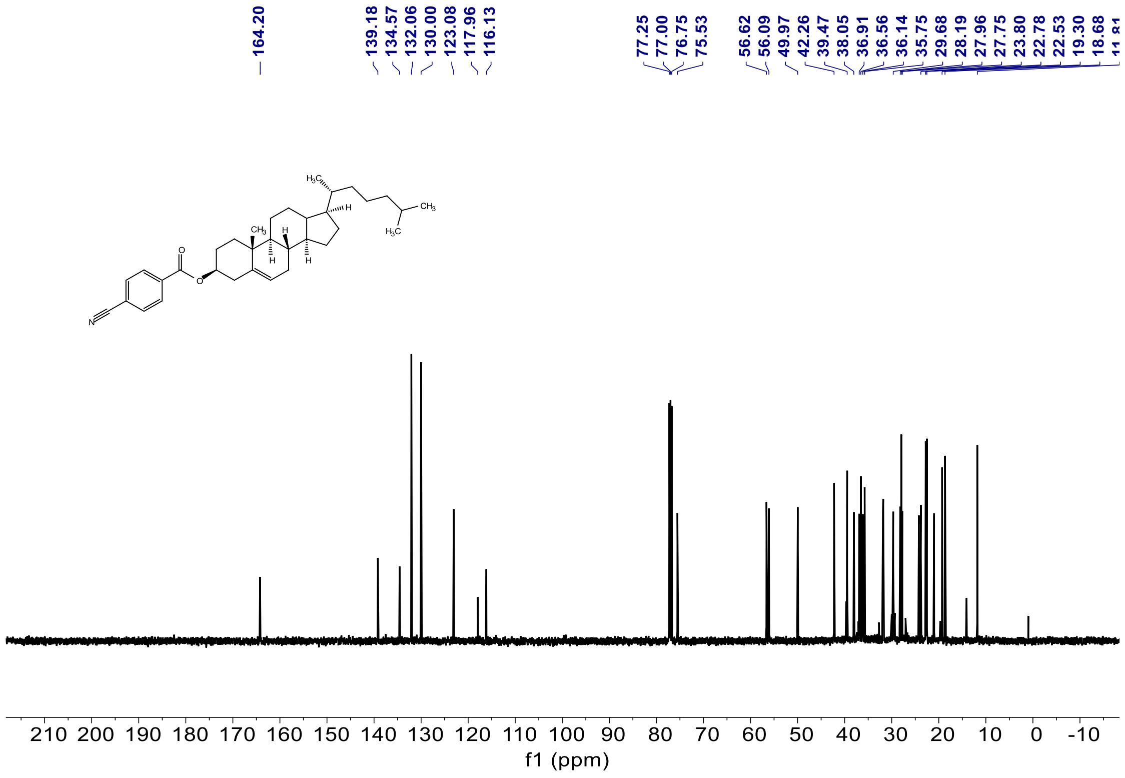

2g' (**^{1}H NMR**, 500 MHz, CDCl3)

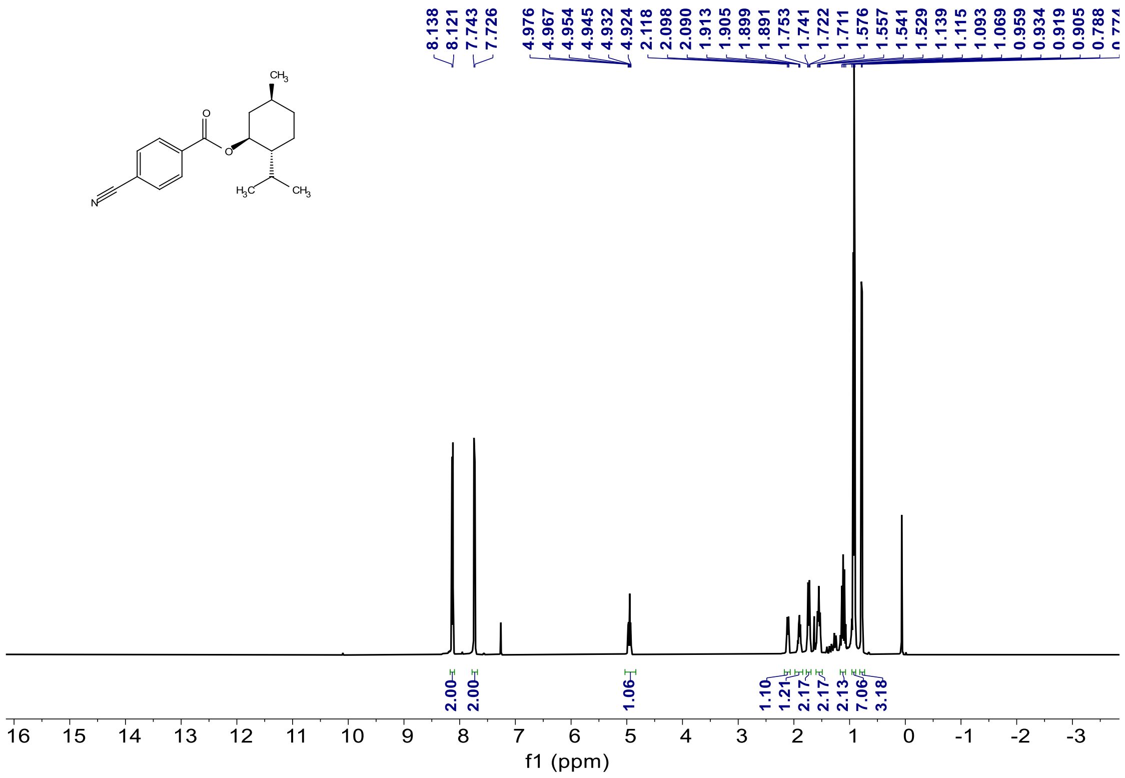

2g' **^{13}C NMR** (125 MHz, CDCl3)

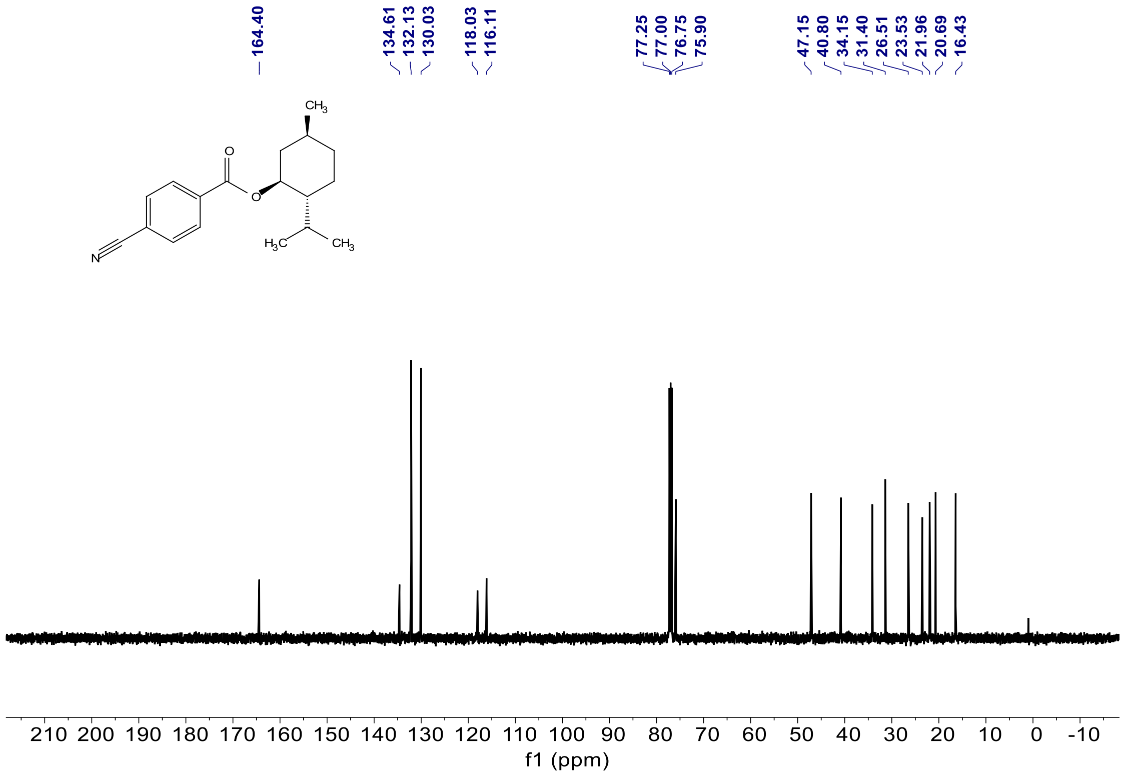

2h' (**^{1}H NMR**, 500 MHz, CDCl$_3$)

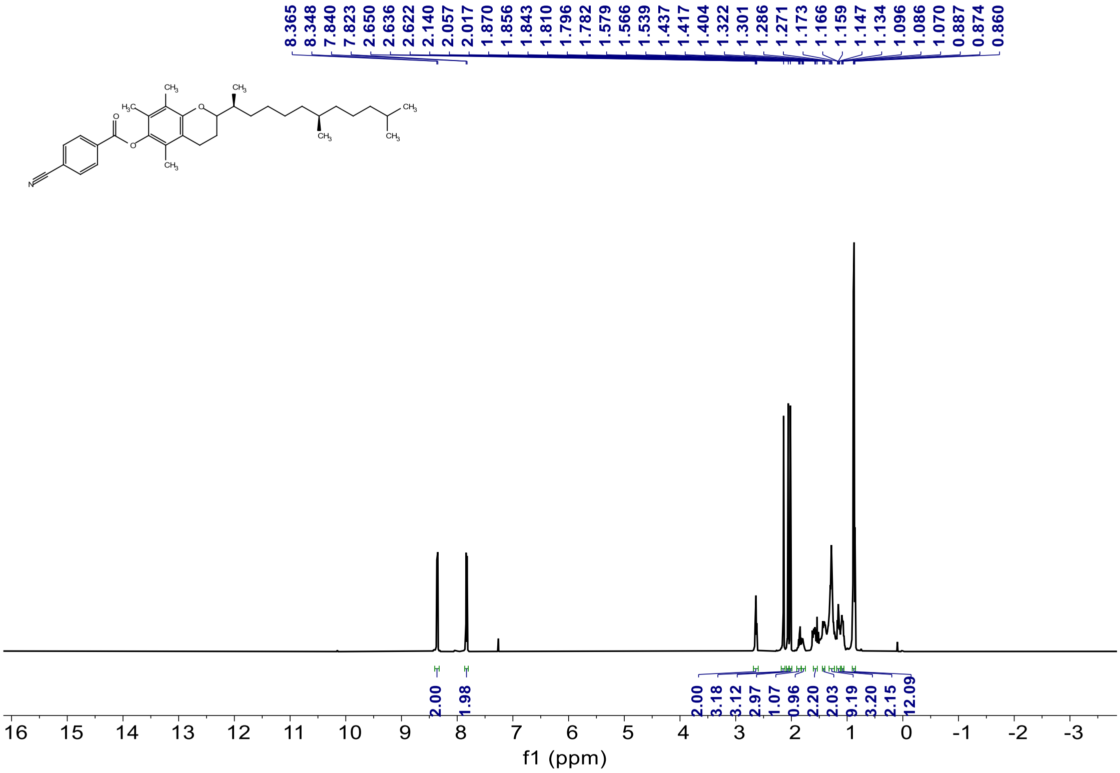

2h' **^{13}C NMR** (125 MHz, CDCl$_3$)

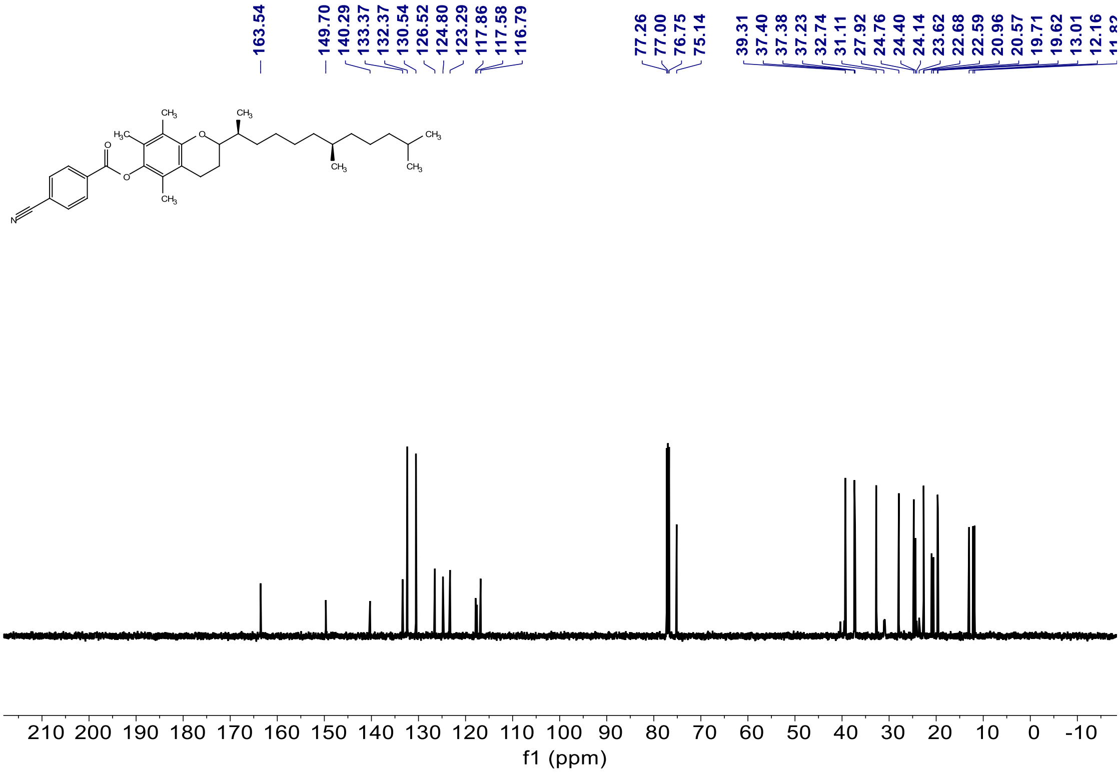

2i' (**^{1}H NMR**, 400 MHz, $CDCl_3$)

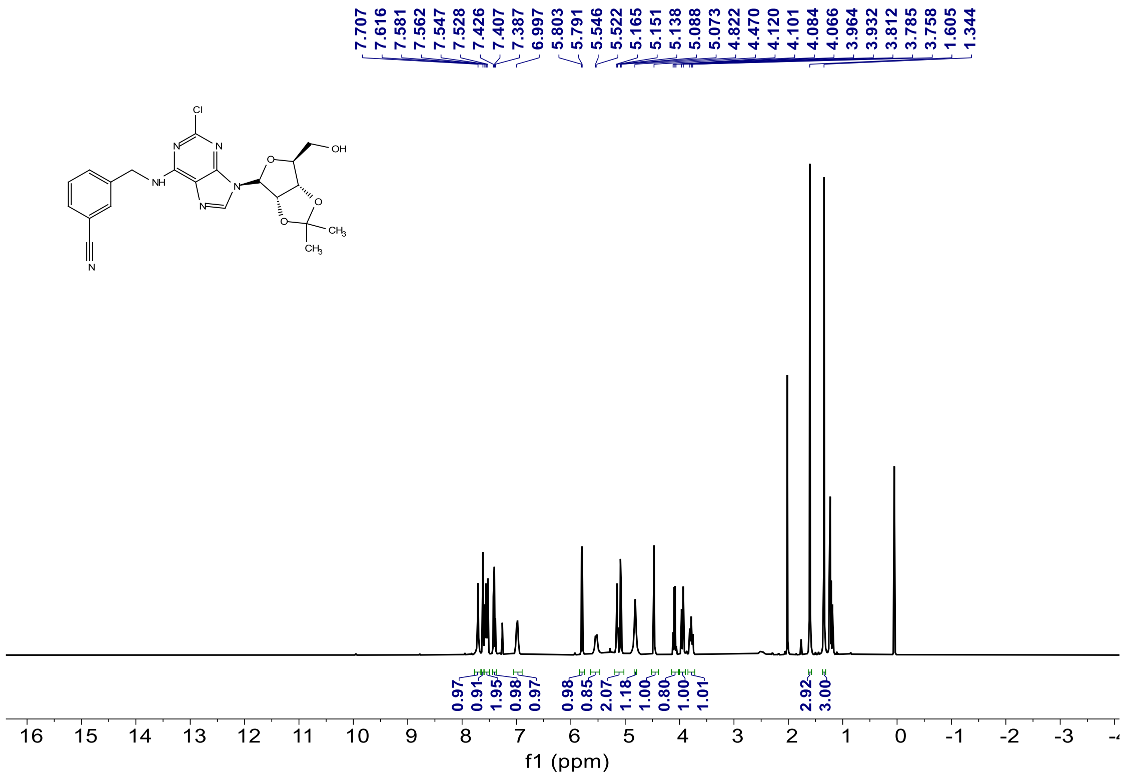

2i' ^{13}C NMR (100 MHz, $CDCl_3$)

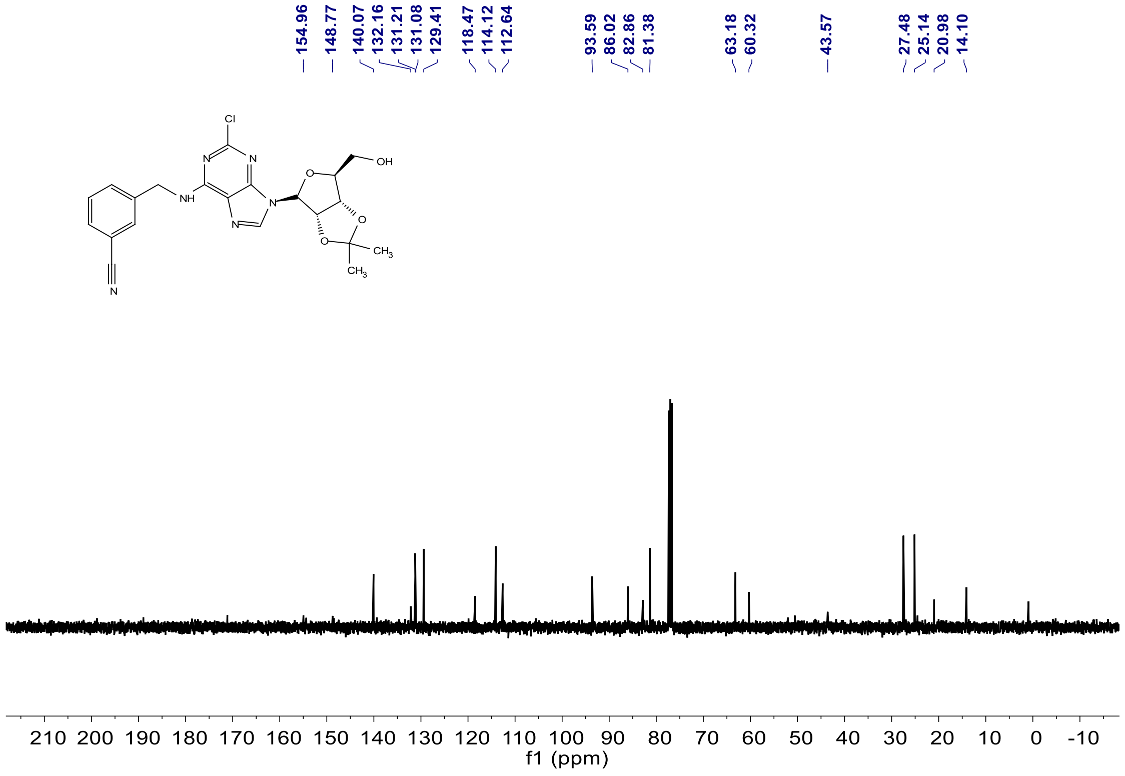

2j' (**^{1}H NMR**, 400 MHz, CDCl3)

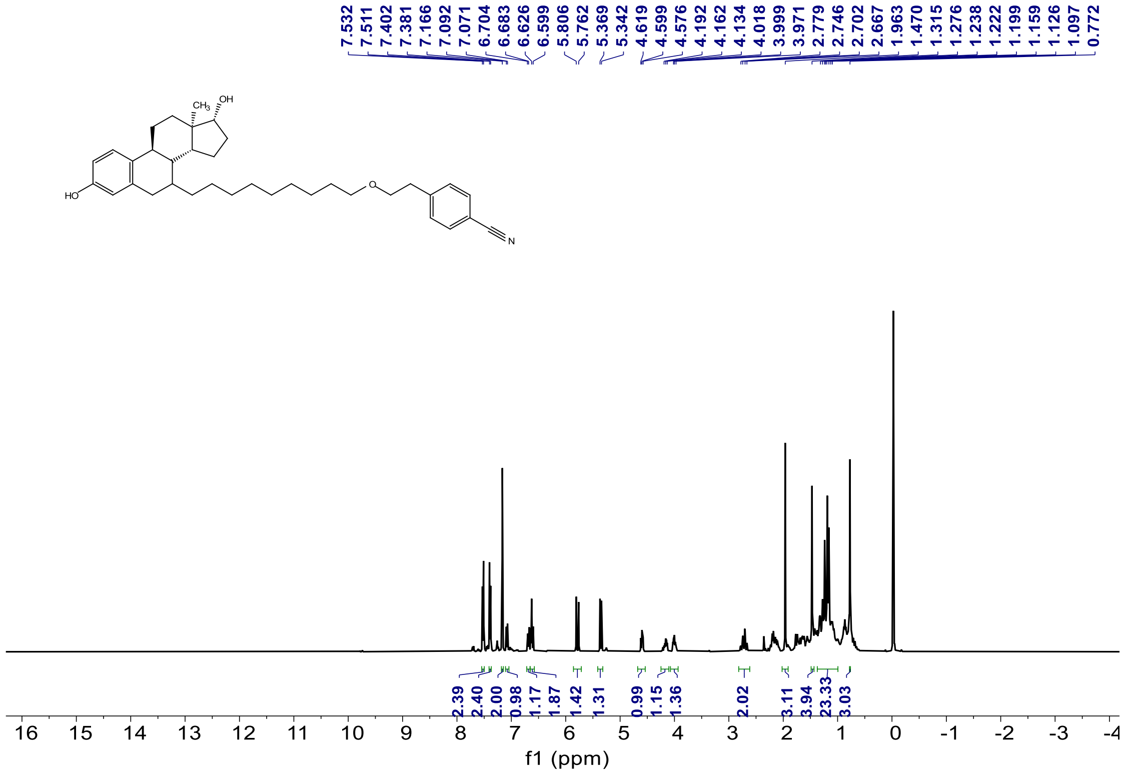

2j' ^{13}C NMR (100 MHz, CDCl3)

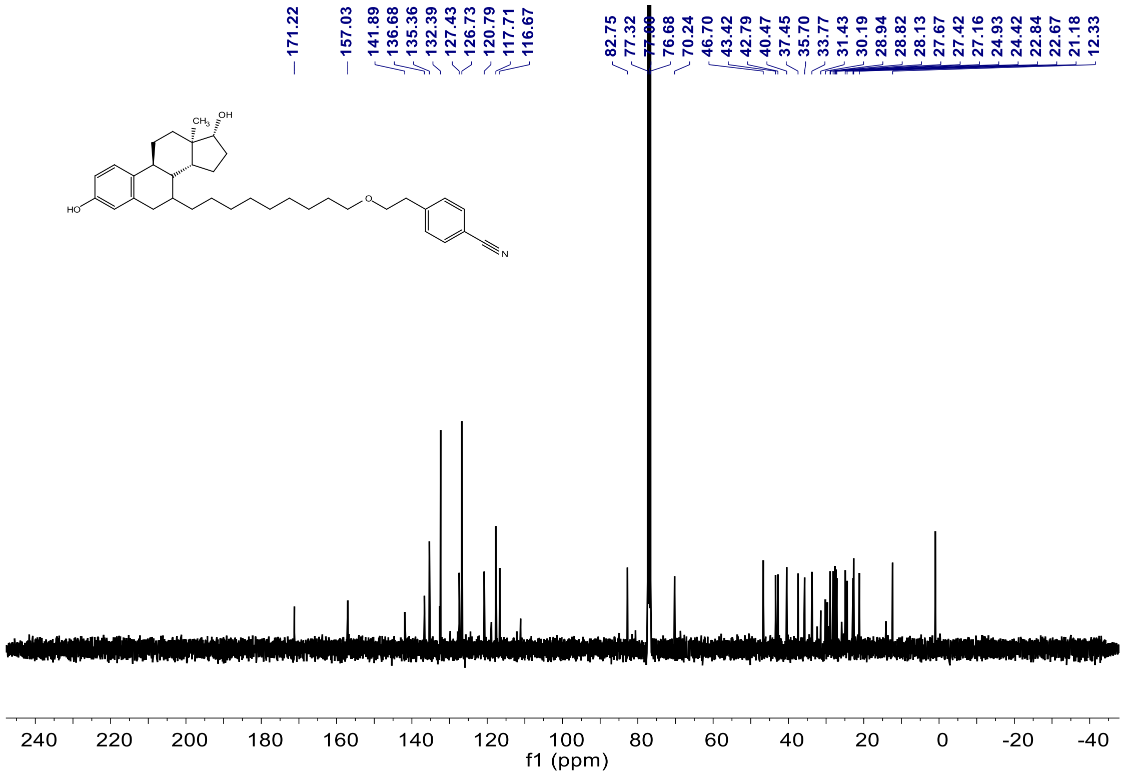

4a (**^{1}H NMR**, 500 MHz, $CDCl_3$)

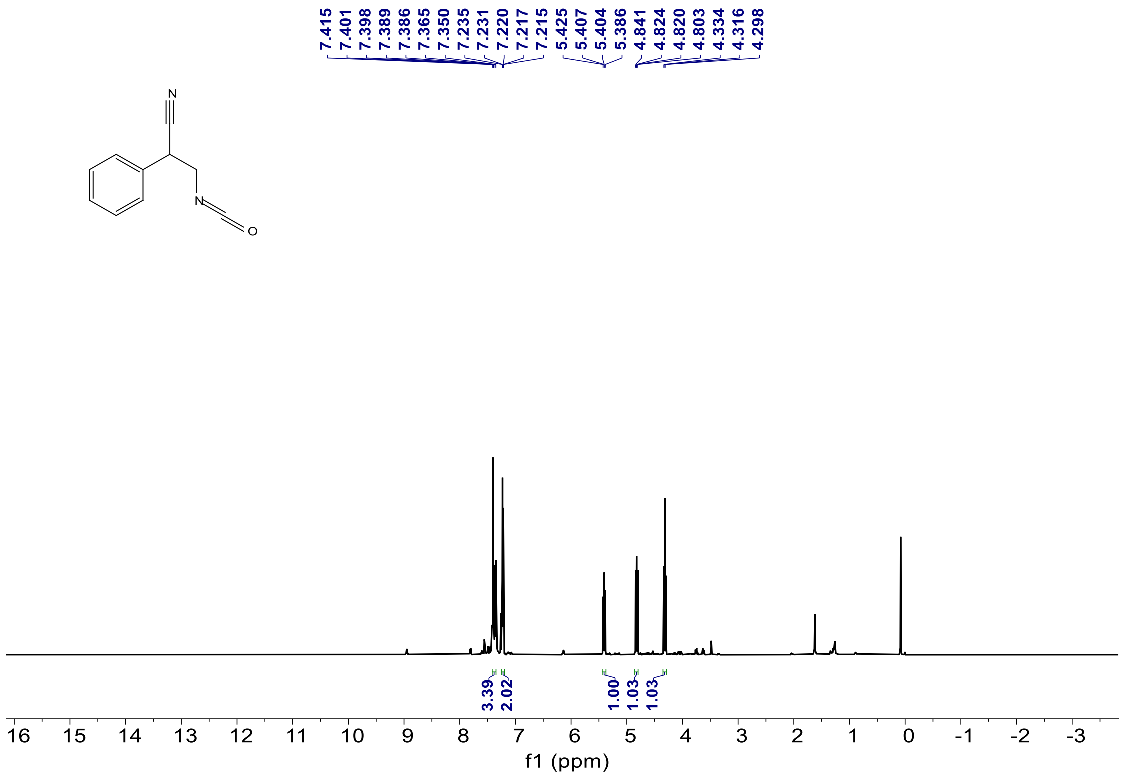

4a ^{13}C NMR (125 MHz, $CDCl_3$)

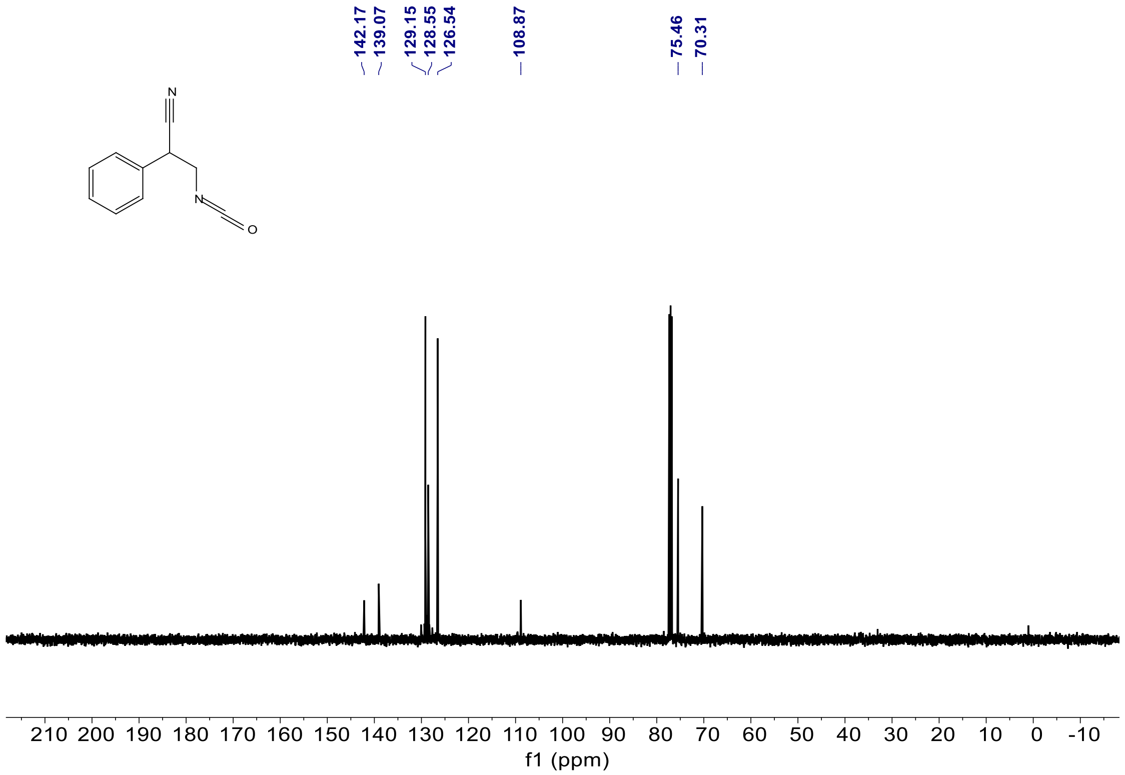

4b (**^{1}H NMR**, 500 MHz, CDCl$_3$)

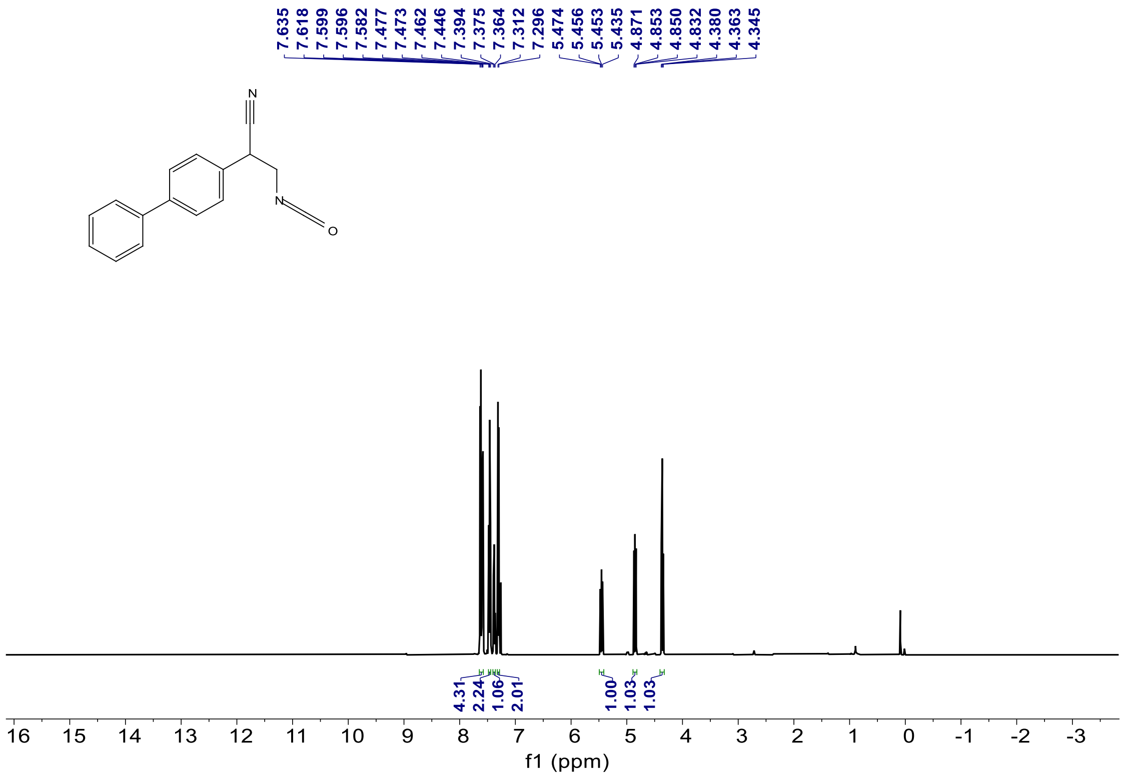

4b **^{13}C NMR** (125 MHz, CDCl$_3$)

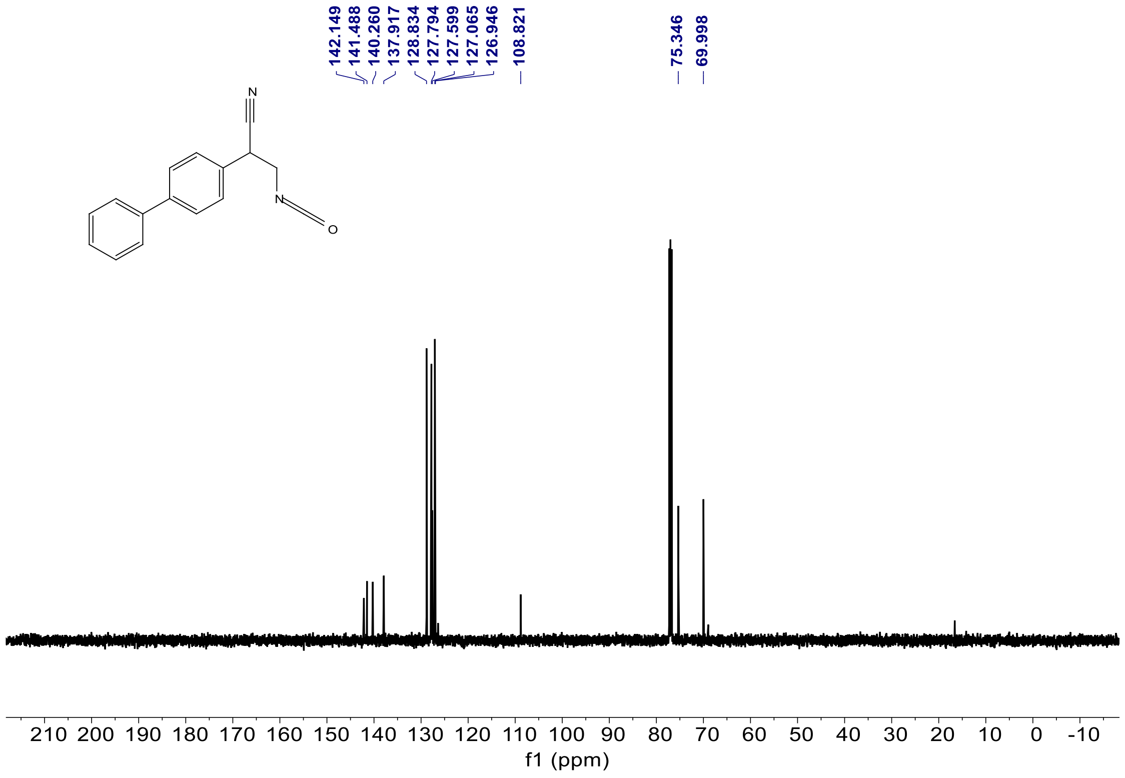

4c (**^{1}H NMR**, 500 MHz, $CDCl_3$)

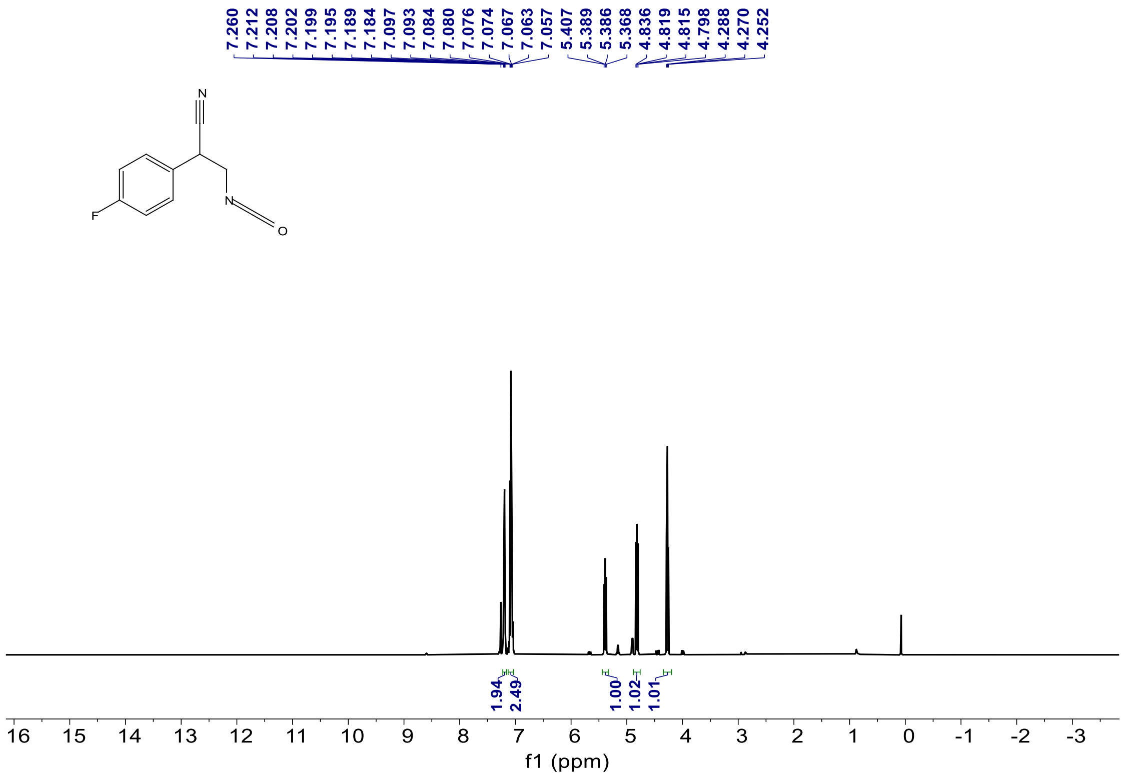

4c ^{13}C NMR (125 MHz, $CDCl_3$)

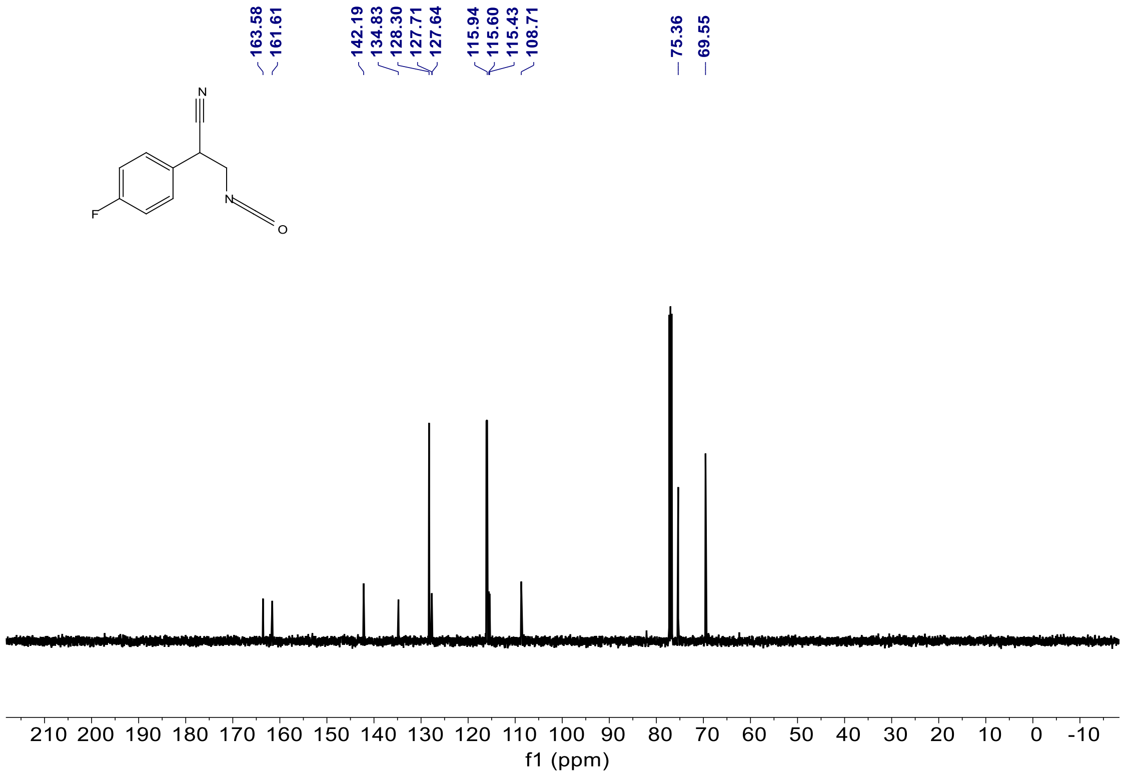

4d (**^{1}H NMR**, 500 MHz, $CDCl_3$)

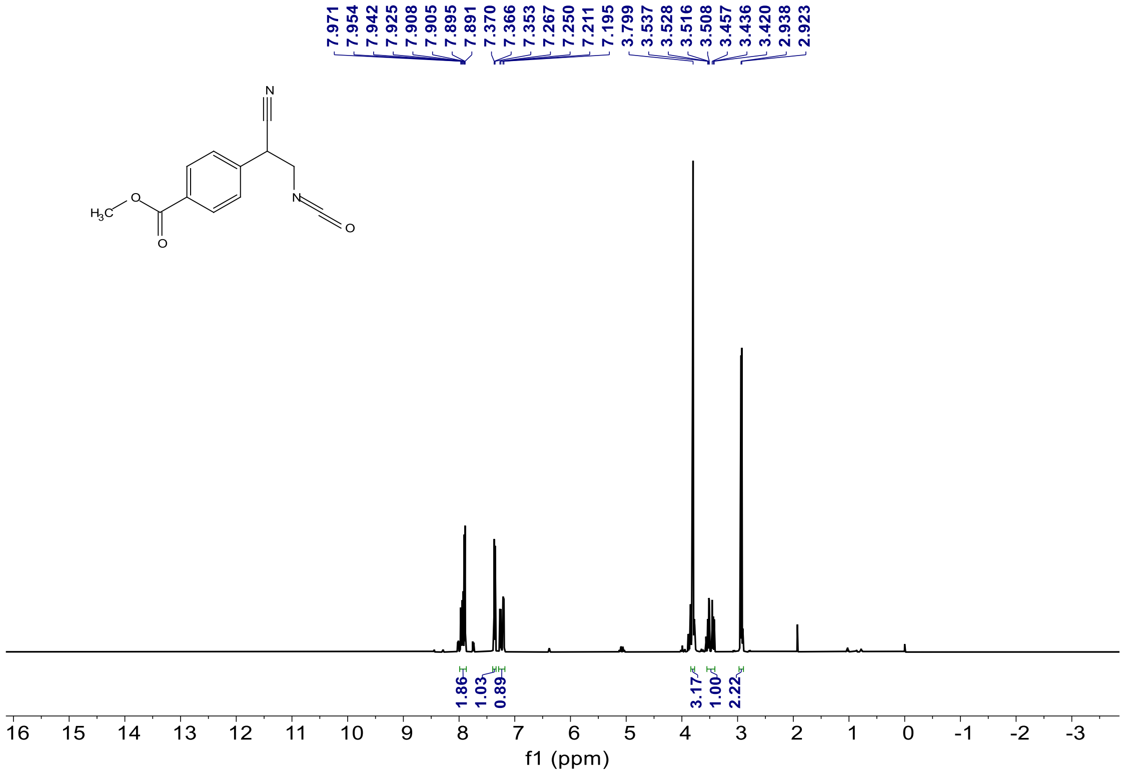

4d **^{13}C NMR** (125 MHz, $CDCl_3$)

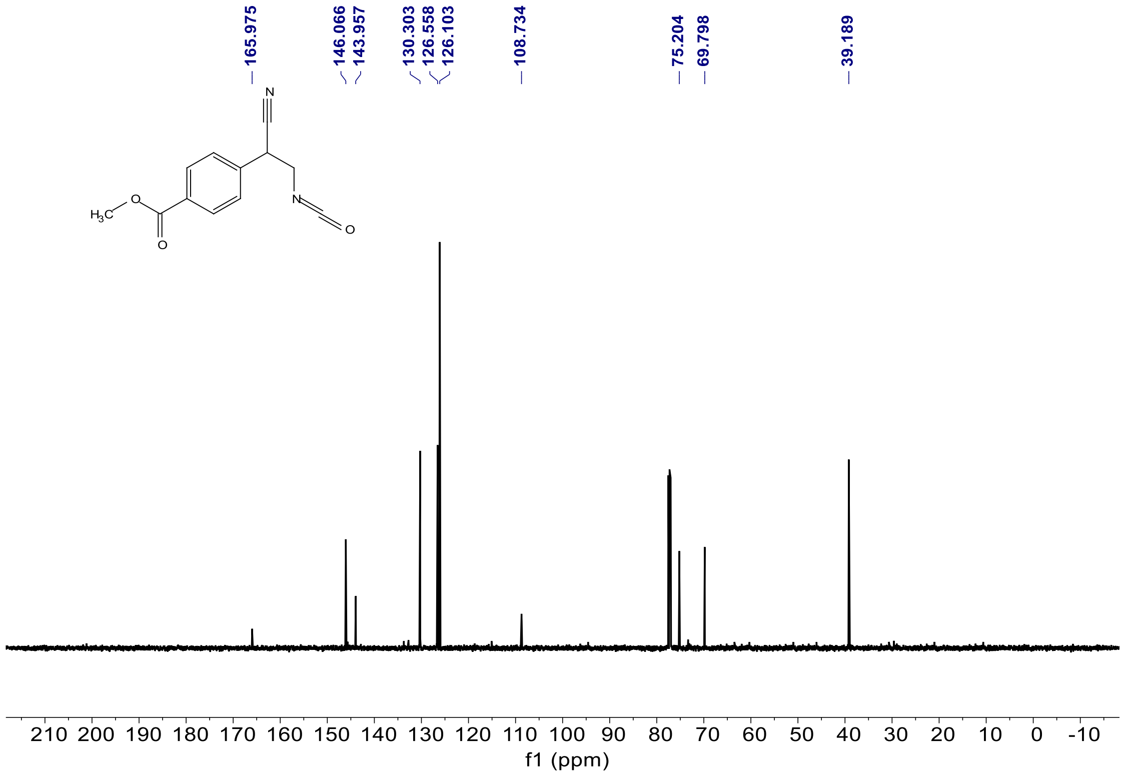

4e ([1]**H NMR**, 500 MHz, $CDCl_3$)

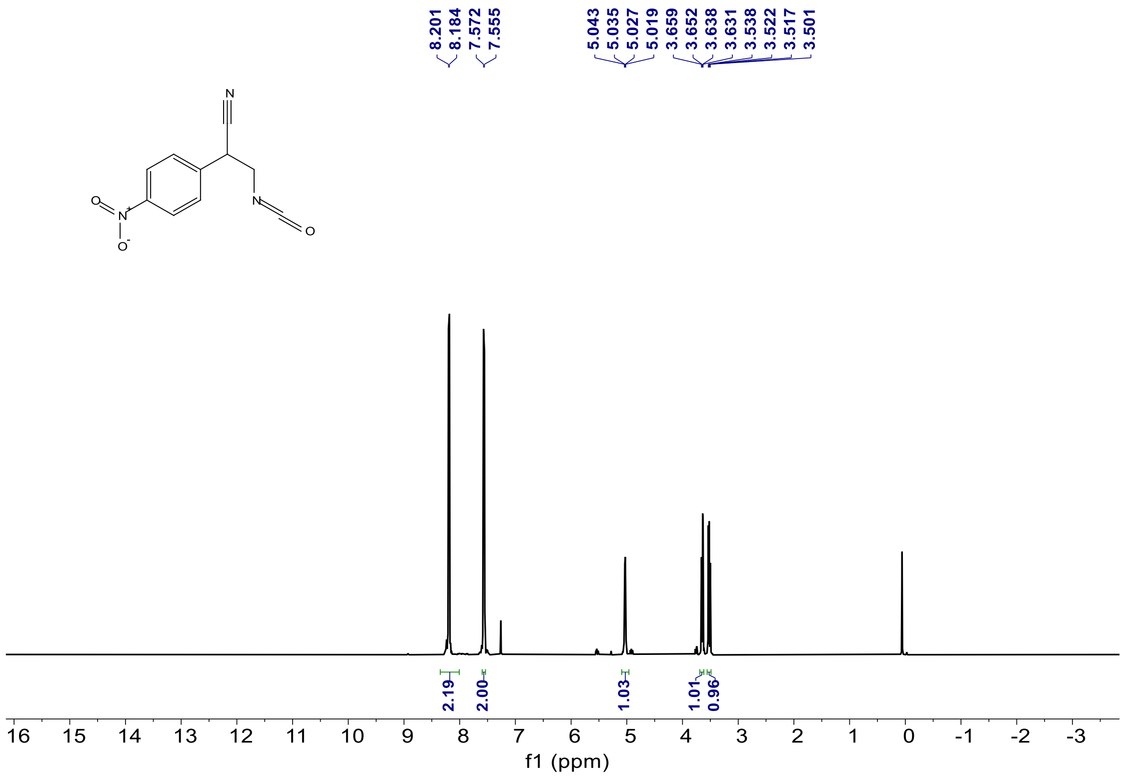

4e [13]**C NMR** (125 MHz, $CDCl_3$)

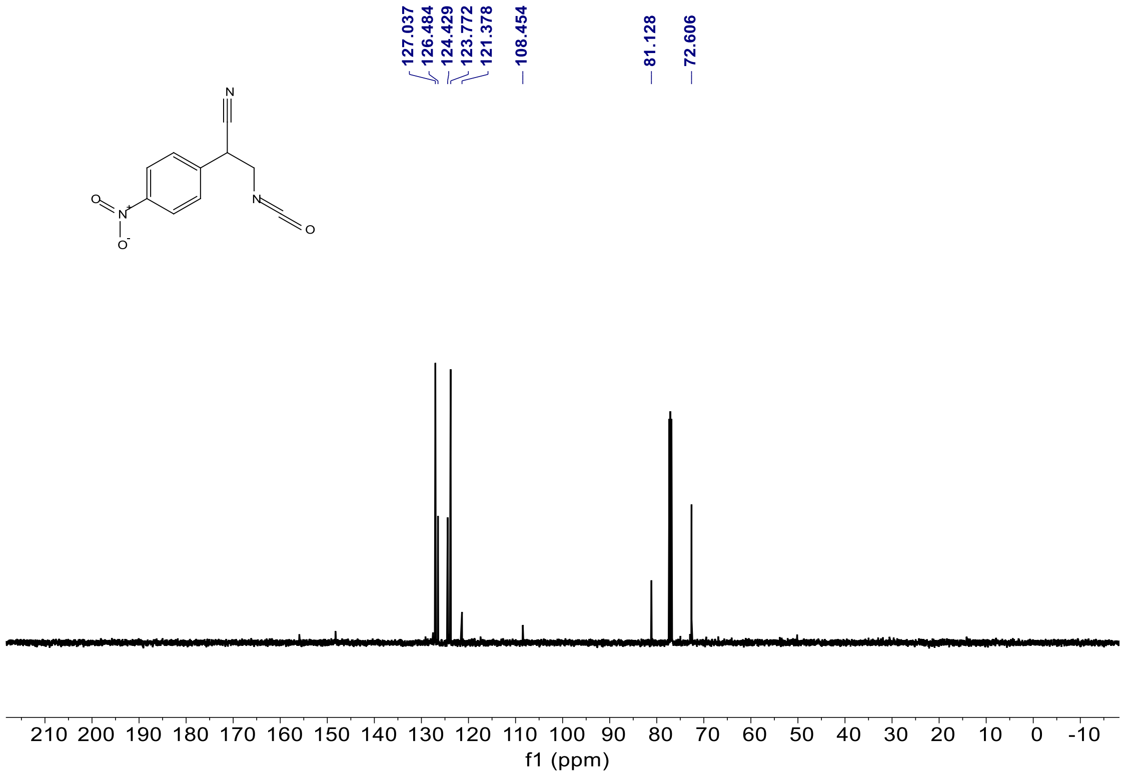

4f ([1]**H NMR**, 500 MHz, $CDCl_3$)

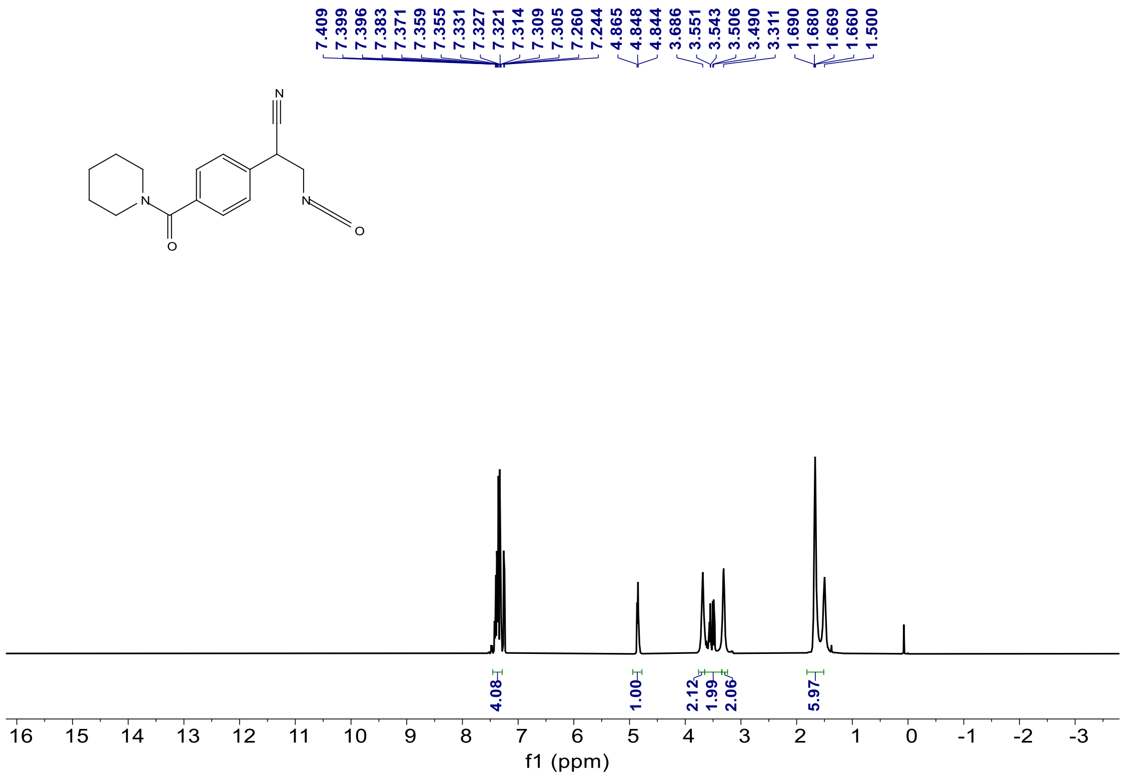

4f [13]**C NMR** (125 MHz, $CDCl_3$)

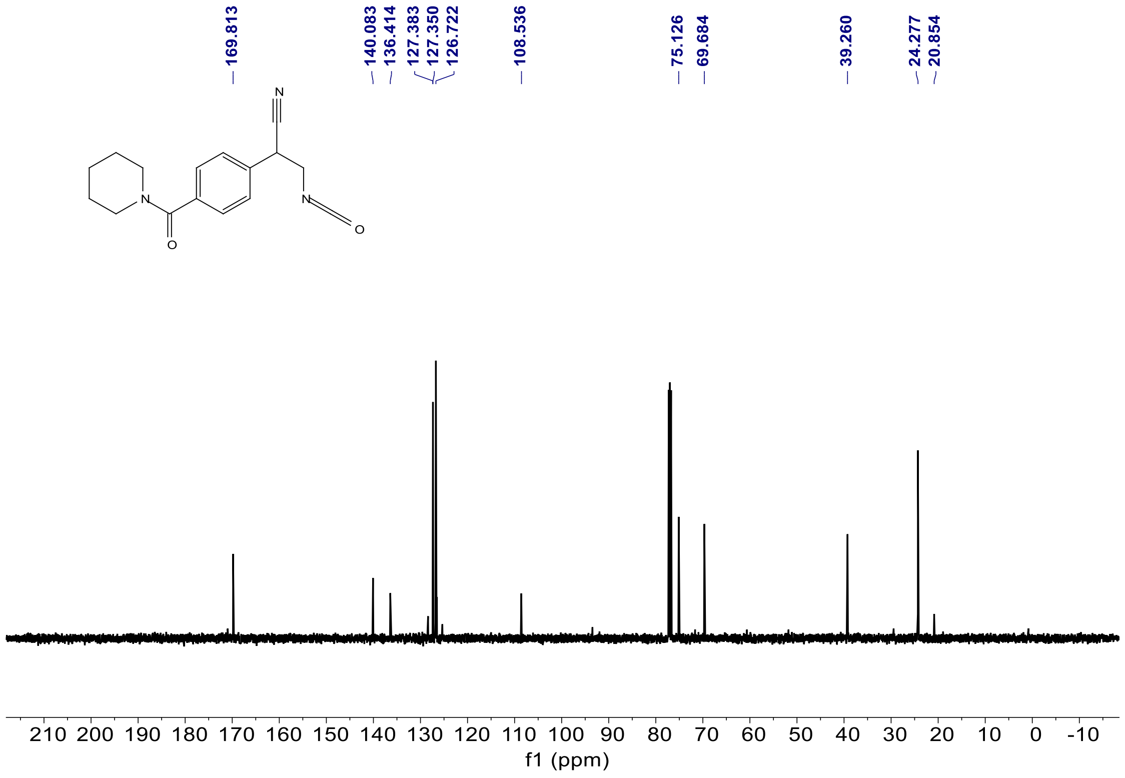

4g ([1]**H NMR**, 500 MHz, CDCl3)

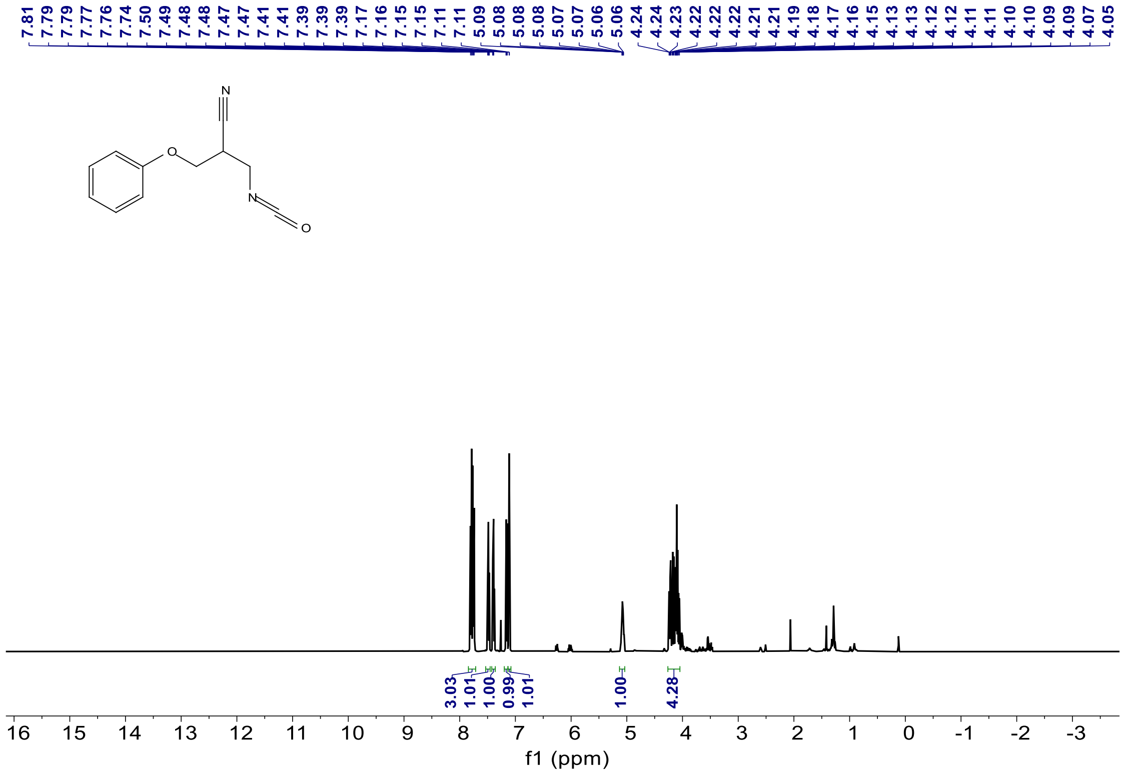

4g [13]**C NMR** (125 MHz, CDCl3)

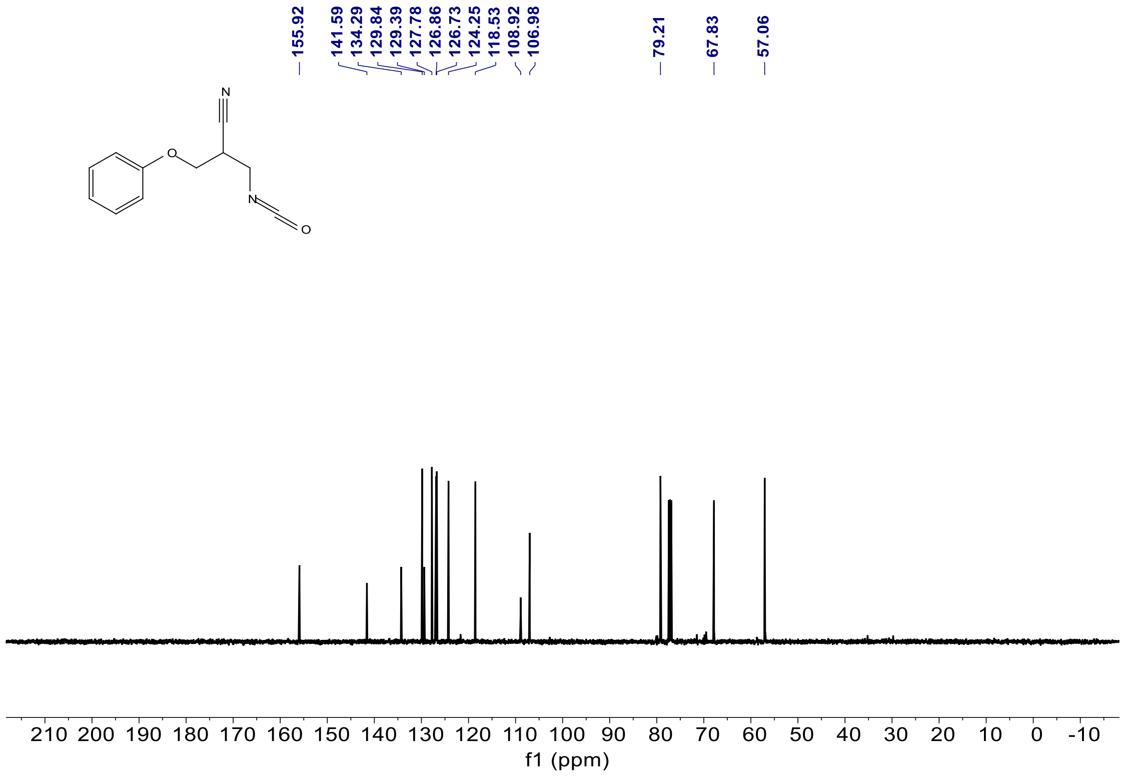

4h (^{1}**H NMR**, 500 MHz, $CDCl_3$)

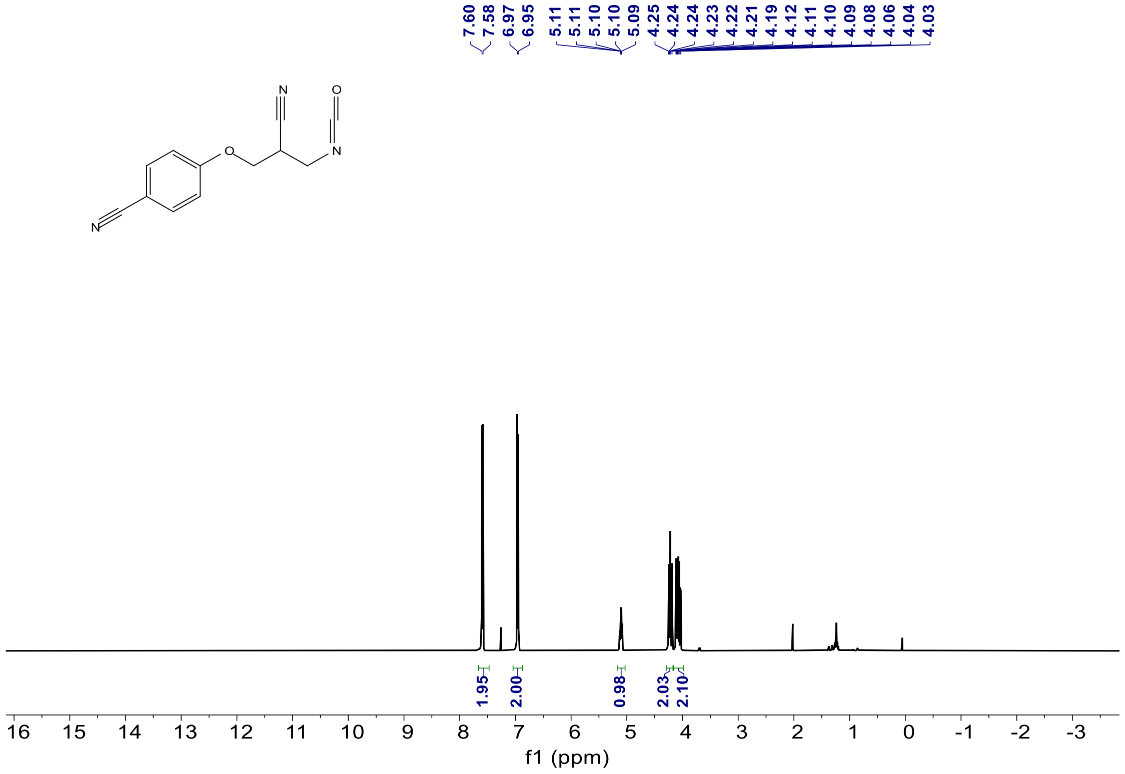

4h ^{13}**C NMR** (125 MHz, $CDCl_3$)

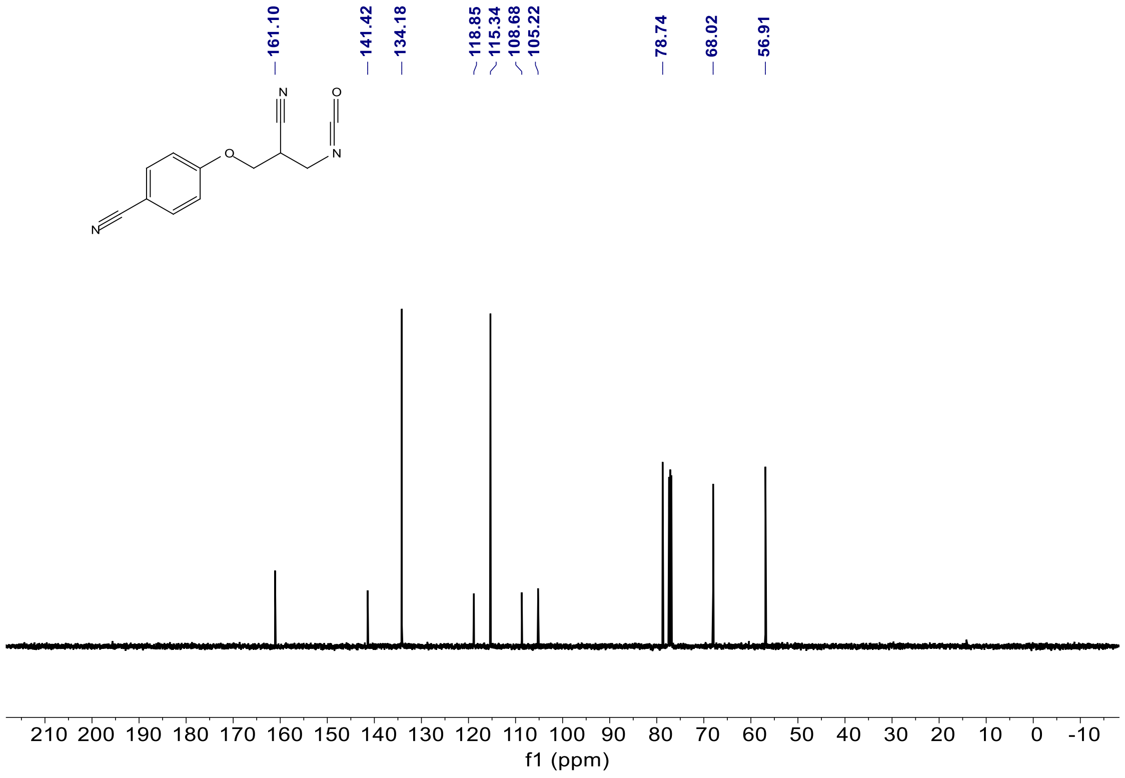

4i (^{1}**H NMR**, 500 MHz, $CDCl_3$)

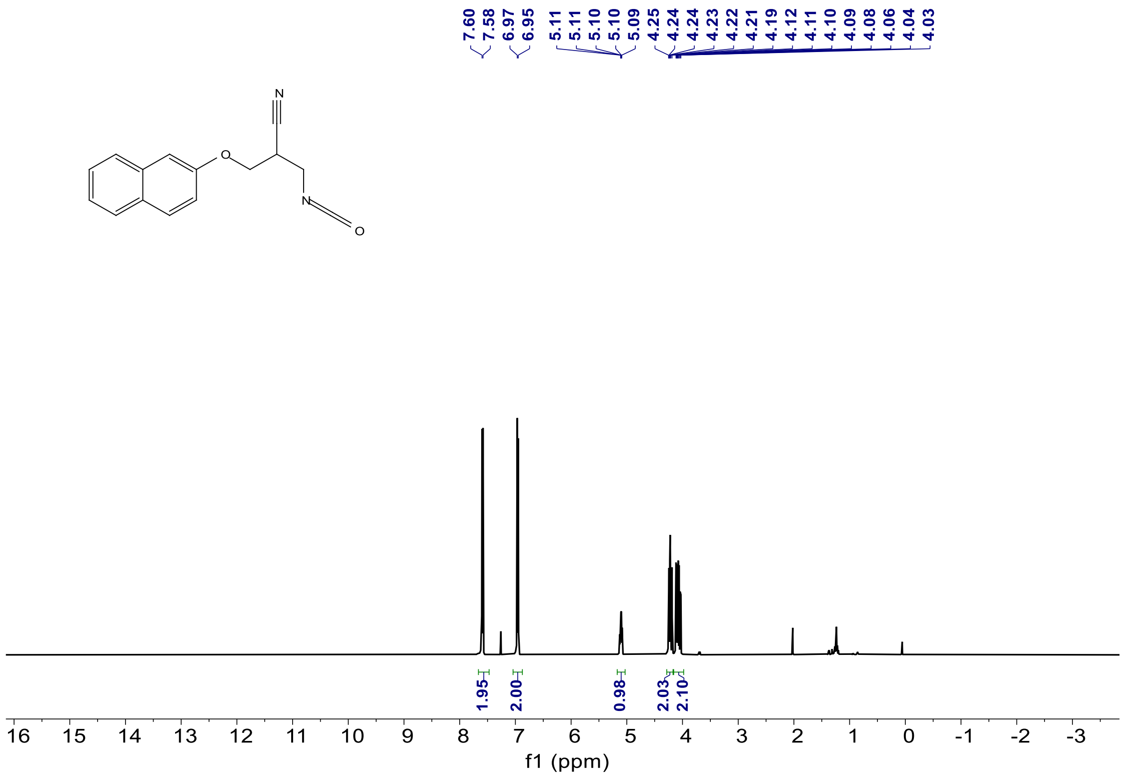

4i ^{13}**C NMR** (125 MHz, $CDCl_3$)

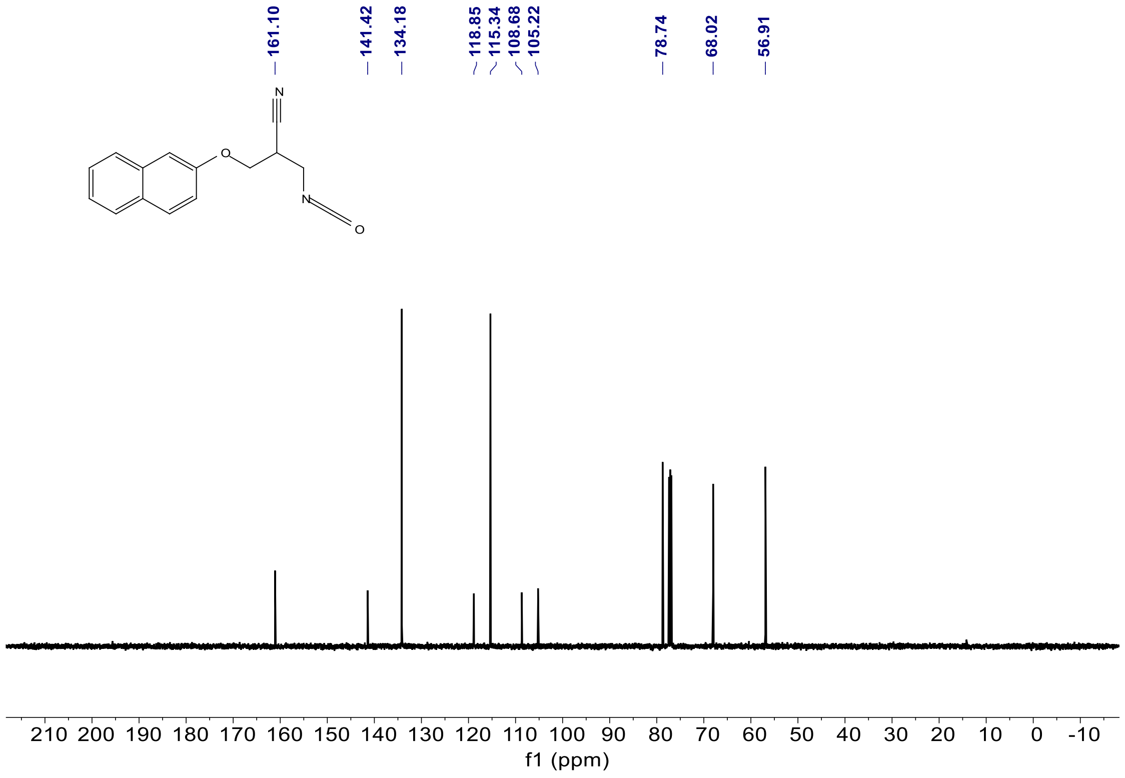

4j (**^{1}H NMR**, 500 MHz, $CDCl_3$)

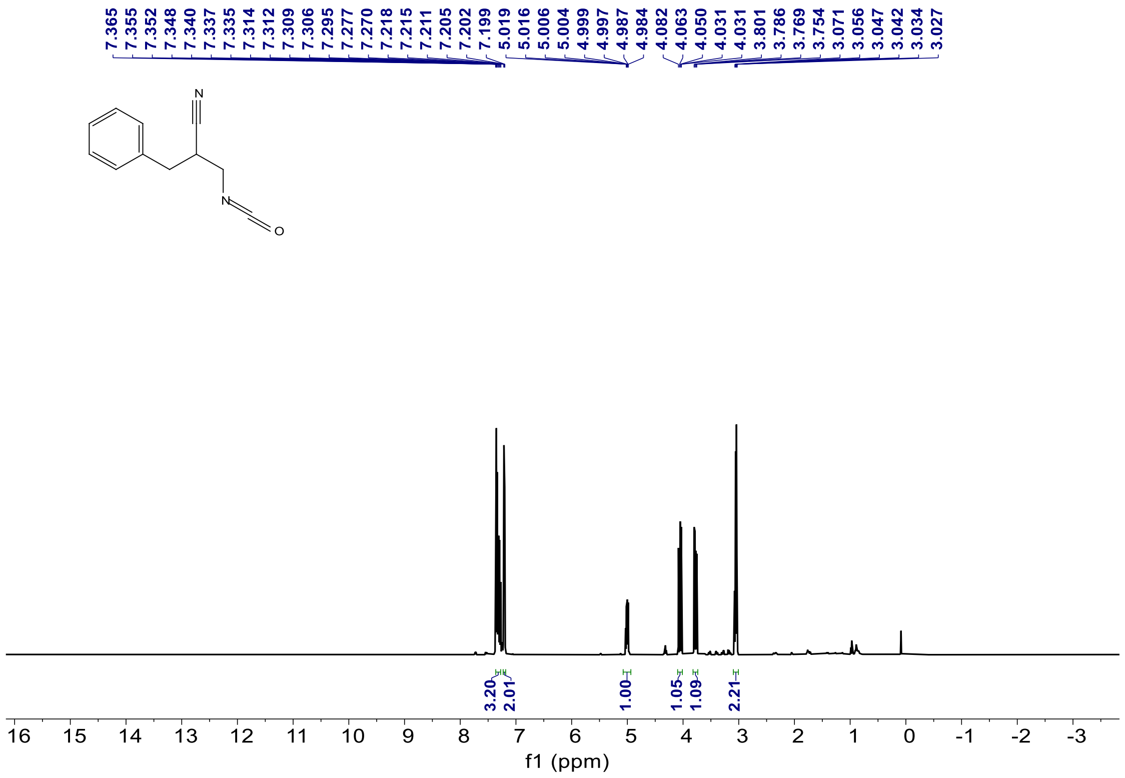

4j ^{13}**C NMR** (125 MHz, $CDCl_3$)

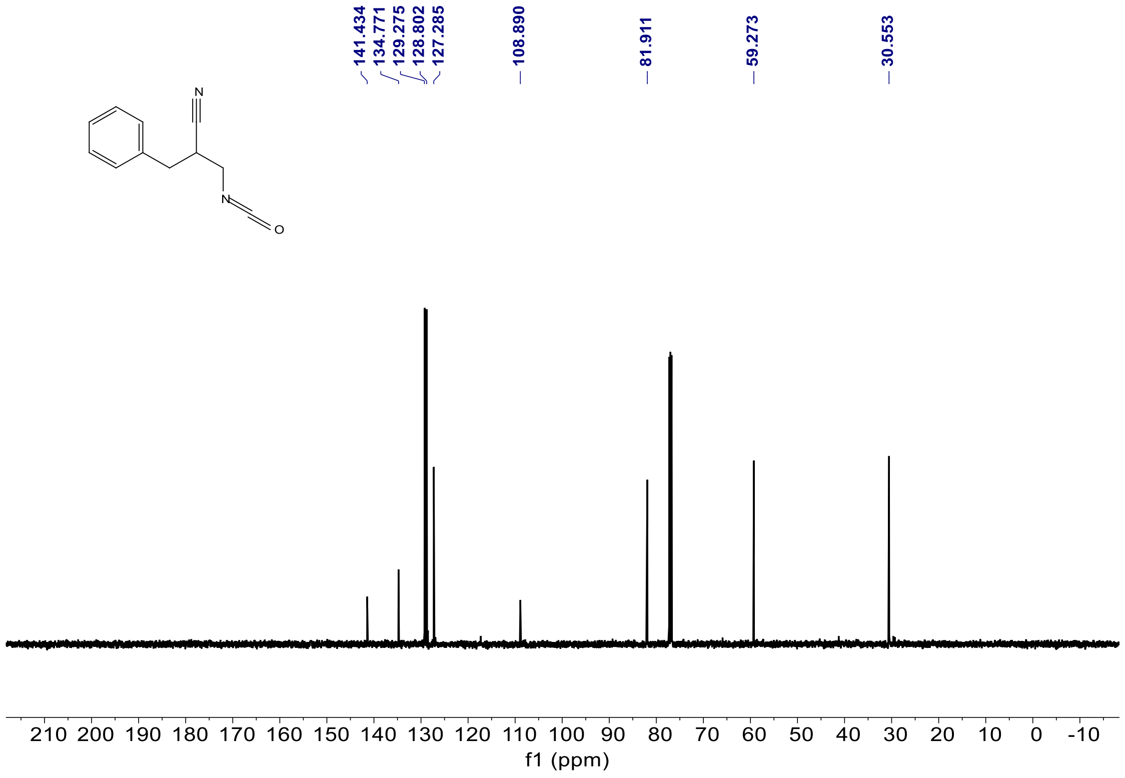

4k ([1]**H NMR**, 500 MHz, $CDCl_3$)

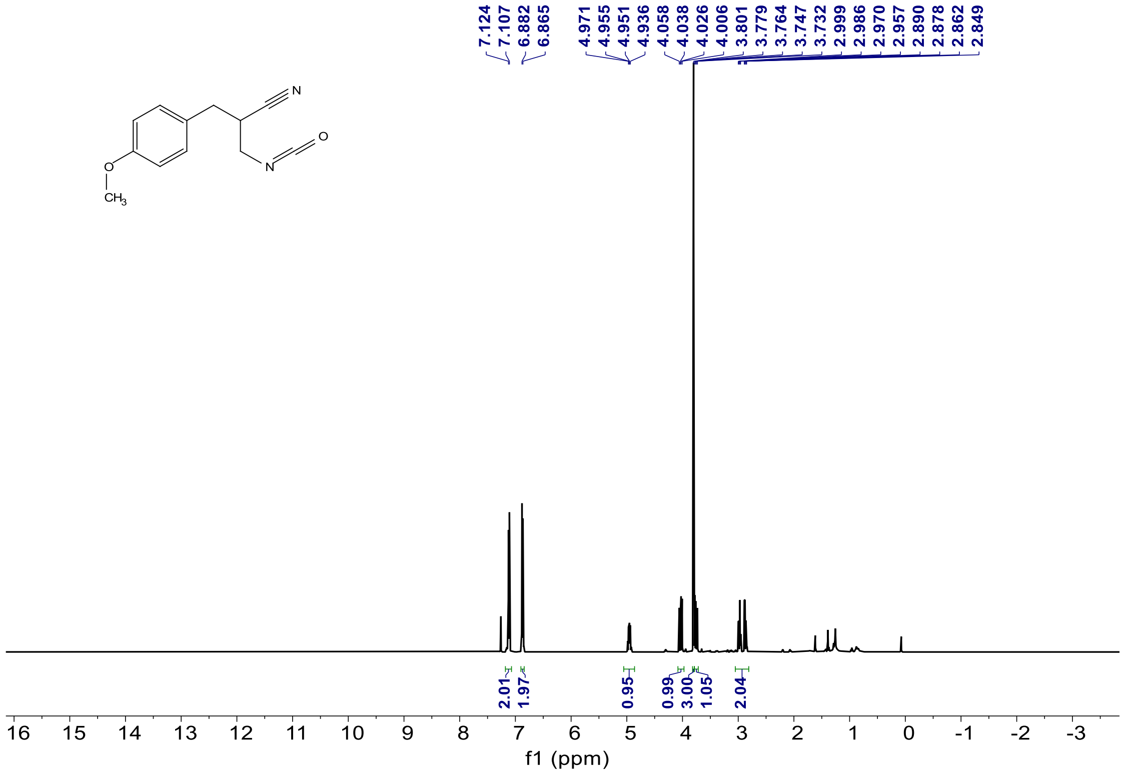

4k [13]**C NMR** (125 MHz, $CDCl_3$)

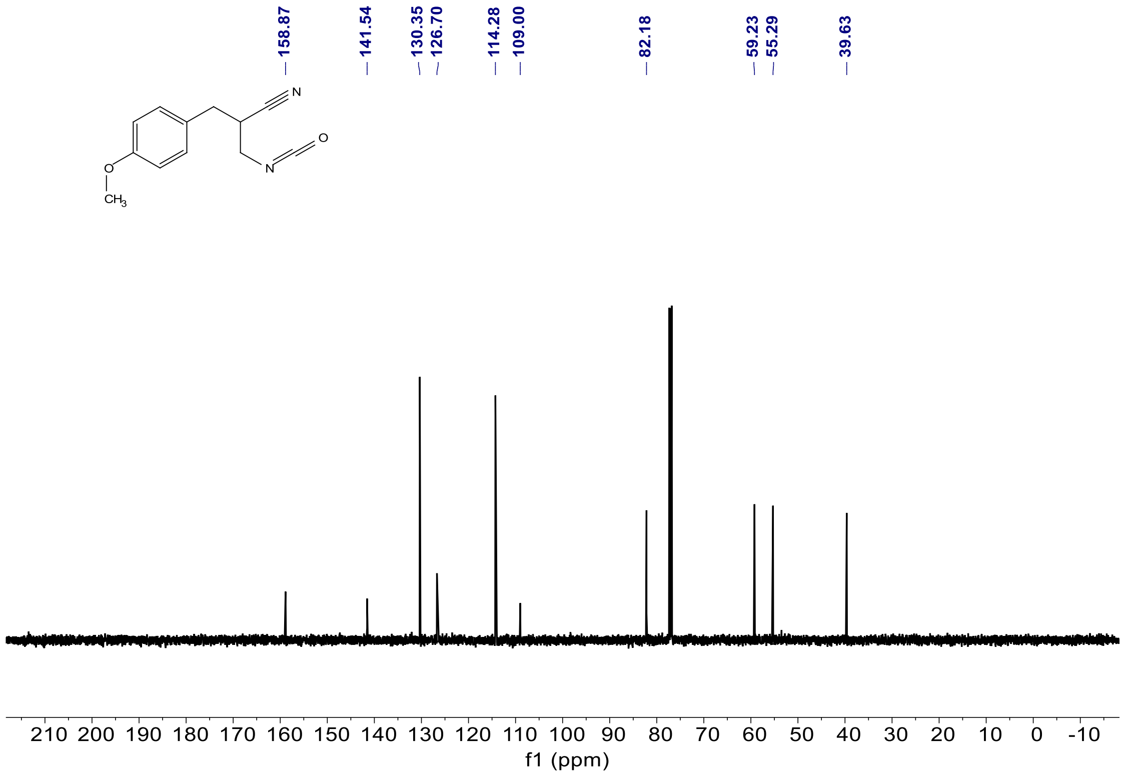

4l (^{1}**H NMR**, 500 MHz, $CDCl_3$)

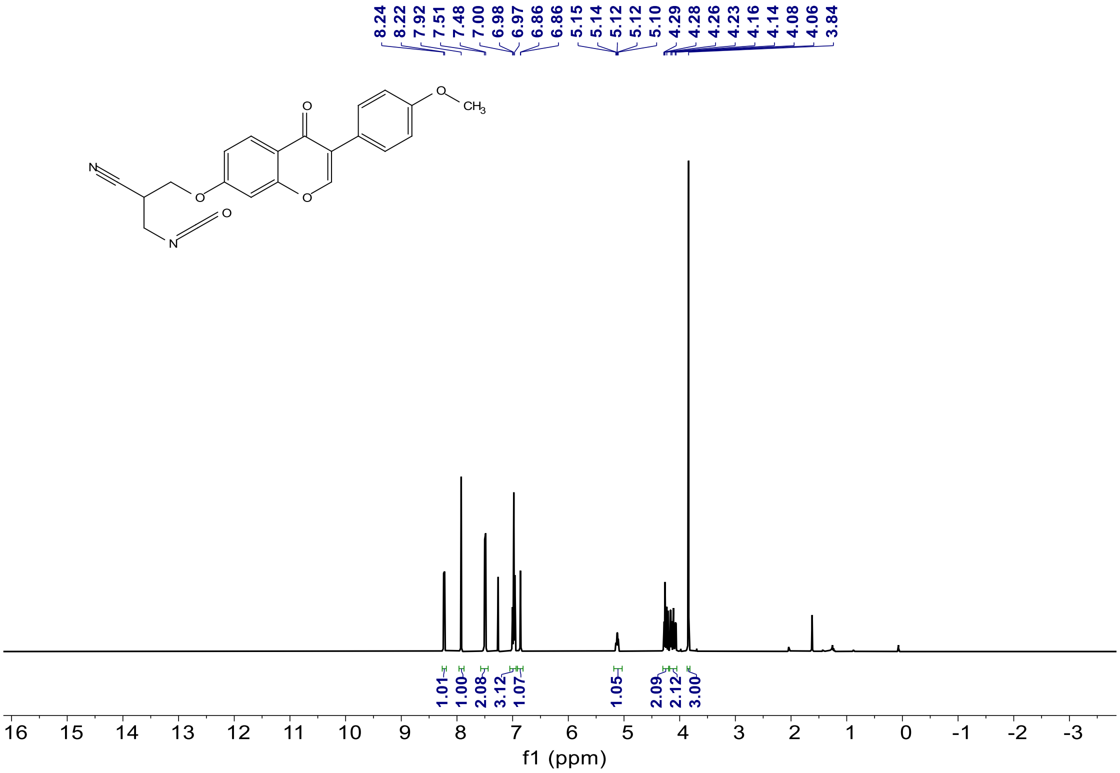

4l ^{13}**C NMR** (125 MHz, $CDCl_3$)

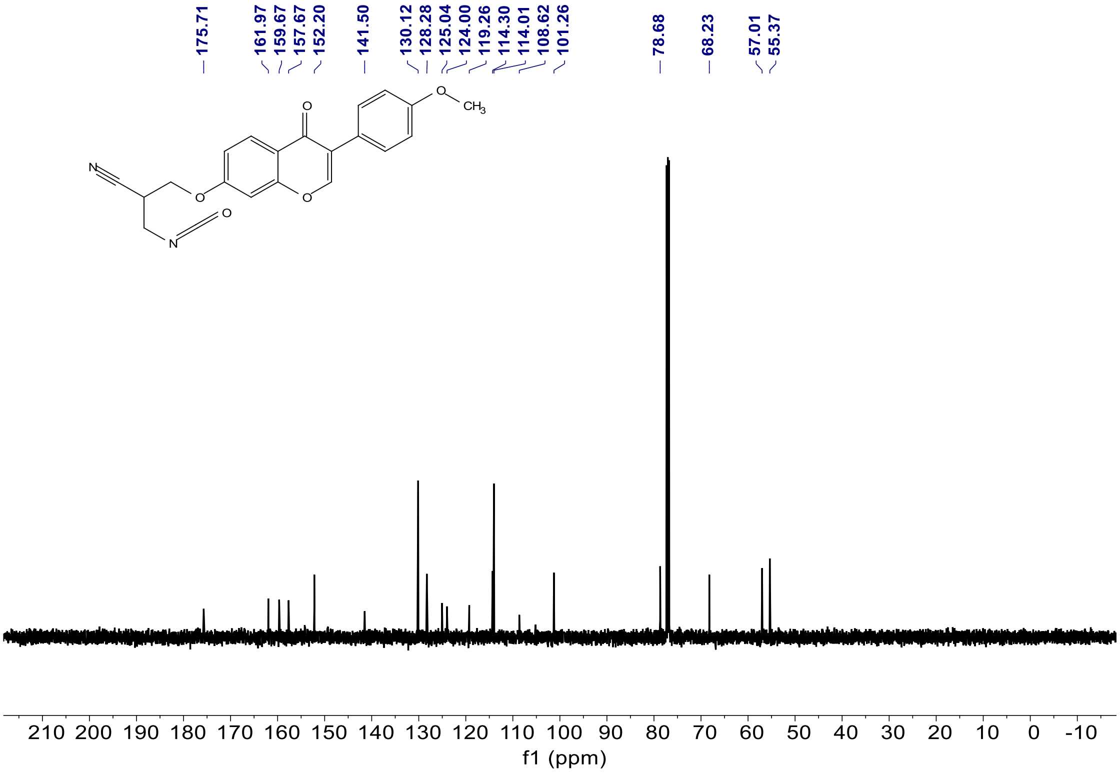

Supplement: Supplementary file 1 [file molecules-29-06016-s001.zip › molecules-3361290-supplementary.pdf]
